# Mapping Asia Plants: Plant Diversity and a Checklist of Vascular Plants in Indonesia

**DOI:** 10.3390/plants13162281

**Published:** 2024-08-16

**Authors:** Jing Sun, Bo Liu, Himmah Rustiami, Huiyun Xiao, Xiaoli Shen, Keping Ma

**Affiliations:** 1 State Key Laboratory of Vegetation and Environmental Change, Institute of Botany, Chinese Academy of Sciences, Beijing 100093, China; sunjing@ibcas.ac.cn (J.S.); xiaohuiyun@ibcas.ac.cn (H.X.); 2 University of Chinese Academy of Sciences, Beijing 100049, China; 3 College of Life and Environmental Sciences, Minzu University of China, Beijing 100081, China; boliu@muc.edu.cn; 4 Herbarium Bogoriense, Research Center for Biosystematics and Evolution, National Research and Innovation Agency (BRIN), Cibinong, Bogor 16911, Indonesia; himmah.rustiami@brin.go.id

**Keywords:** Indonesia, tropical island, flora, checklist, biodiversity conservation

## Abstract

Indonesia, located in Southeast Asia, is the world’s largest tropical island country. It is globally recognized as a unique center of biodiversity in the Asian–Australian transitional zone. To date, however, no national plant checklist of Indonesia has been published. Here, we collected botanical information on the flora of Indonesia and presented for the first time a checklist of known native species of vascular plants in the country. Our checklist included 30,466 species belonging to 2968 genera and 317 families, representing 8.7% of the 351,180 vascular plant species worldwide. Among the seven regions, Sumatra had the highest number of species at 10,902, followed by Indonesian New Guinea (9935), Java (9289), Kalimantan (9191), Sulawesi (7048), Maluku (5294), and Lesser Sunda Islands (4514). In contrast, Indonesian New Guinea had a much higher proportion of locally endemic species than other regions (59%). The seven regions were divided into three phytogeographical areas: the Sunda Shelf, the Sahul Shelf, and the Wallacea, based on similarities in floristic composition. Our checklist for Indonesia provides basic information for biodiversity conservation and associated research.

## 1. Introduction

Asia is lagging behind in plant inventories compared to other continents in the world [1,2,3], and even in the Global Biodiversity Information Facility, the world’s largest biodiversity database, there are relatively few data records. Asia has a larger area but more severe loss of biodiversity [4,5]. The Mapping Asia Plants (MAP) project was initiated in 2015 by the Asian Biodiversity Conservation and Database Network (ABCDNet), administered by the Biodiversity Committee of the Chinese Academy of Sciences, and aimed to integrate plant diversity data to establish a plant checklist for Asia and each of its 48 countries, providing baseline data for biodiversity conservation and research [4]. The project divided Asia into six sub-regions: North Asia, Northeast Asia, Southeast Asia, Southwest Asia, South Asia, and Central Asia [6]. Southeast Asia is experiencing significant loss of biodiversity, with rich but poorly documented plant diversity, and faces great challenges in developing plant inventories [7,8,9]. Indonesia is the largest country in Southeast Asia, known as a megadiverse country with unusual species richness and endemism [10,11], but there is still no comprehensive plant checklist at a national scale.

**Figure 1:**
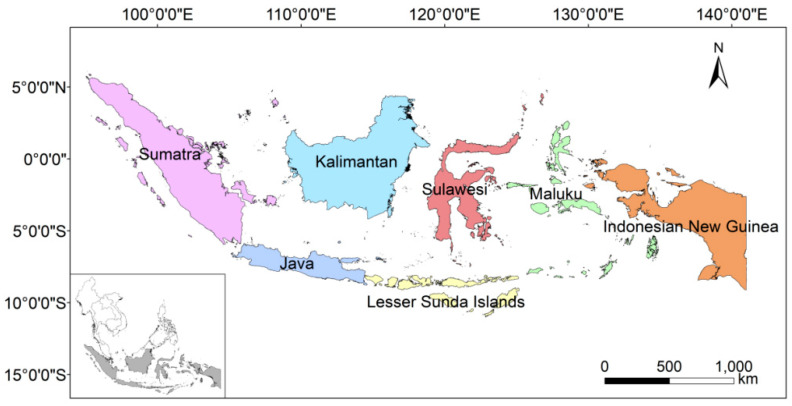
Map of Indonesia with seven geographical regions. The inset shows the location of Indonesia in Southeast Asia.

Indonesia is located between the continents of Asia and Australia, stretching across the equator region from 6° N to 11° S and from 95° E to 141° E, with a land area of 1.91 million km^2^ [12,13]. It is the largest archipelago in the world, consisting of more than 17,000 islands that are divided into seven geographical regions: Java, Sumatra, Kalimantan, Lesser Sunda Islands, Sulawesi, Maluku, and Indonesian New Guinea [14,15] (Figure 1). The Indonesian archipelago is mainly originated from the Gondwanaland, which is located at the intersection of three plates, namely the Indian, Eurasian, and Pacific plates, making Indonesia one of the most geologically complex regions in the world due to frequent geological activities [16,17]. Three significant collision events had a major impact on the biota in the region [18]. During the Eocene (ca. 45 Ma), the Indian plate collided with Eurasia and plant taxa of Indian origin dispersed to Southeast Asia, leading to the extinction of many Paleocene plants [19]. In the late Oligocene (ca. 25 Ma), the Australian–Sunda collision disrupted the Indonesian throughflow, and moisture that previously flowed between the Pacific Warm Pool and the Indian Ocean fell on the Sundaland; this change in humid climate led to the development of the modern Malaysian flora [19,20]. The third collision was between Australia’s westernmost cape and Sulawesi during the Miocene (ca. 15 Ma), resulting in the first fusion of continental debris from the Sunda and Australian continents [21,22]. Climate-induced sea level changes between the Oligocene and Pleistocene resulted in repeated inundation of the Sunda and Sahul shelves during interglacials [23]. During the early Quaternary, as the magnitude of sea level fluctuations increased, previously submerged continental shelves were exposed during glacials and submerged again during interglacials, a cycle that occurred at least 20 times, with “isolation–convergence” of species in the region, which facilitated species differentiation [23,24,25]. Through his observations of the Malay Archipelago fauna, the British naturalist Wallace [26] discovered a distinct boundary between Southeast Asia and New Guinea–Australia, also known as the “Wallace Line” [27].

Indonesia has a variety of ecosystem types, such as tropical forests, karst, mangroves, and peatlands [11]. Indonesia has 120 million hectares of tropical forest, the third largest area in the world [28]. Tropical rainforests, an important part of Indonesia’s forest ecosystems, are humid, hot, and rich in plant life, mainly located in lowland regions near the equator, like Sumatra, Kalimantan, Papua, and Sulawesi [17,29,30,31]. Tropical monsoon rainforests have markedly seasonal variations throughout the year, with alternating wet and dry seasons, such as seasonal droughts in eastern Java and Lesser Sunda Islands due to the influence of southeast monsoons from Australia [32]. Karst belongs to a unique ecosystem with both aboveground and cave habitats; Indonesia is home to a vast area of karst landscapes, covering 15.4 million hectares [33], with famous karst locations such as at Maros (Sulawesi), Bukit Barisan (Sumatra), Gunungsewu (Java), Sangkulirang (Kalimantan), and Lorentz (Indonesian New Guinea). Mangroves are highly productive and support biodiversity, playing an important role in biodiversity maintenance [34,35]. Mangrove ecosystems in Indonesia are distributed along the coast from Sumatra to New Guinea, covering a very large area of 3.36 million hectares [36]. Peatlands are one of the most important ecosystems on Earth for capturing and storing carbon and are also important habitats for some wildlife [37,38]. The area of peatlands in Indonesia is the fourth largest in the world, at over 20 million hectares [38]. These diverse ecosystems provide Indonesia with a wealth of natural resources as well as important habitats for species. Although Indonesia occupies only 1.3% of the world’s land area, it has an estimated 30,000–40,000 plant species and is home to two biodiversity hotspots, the Sundaland and the Wallacea [39].

Botanical studies in Indonesia date back to the colonial period, when European botanists and naturalists began to explore the flora of the archipelago. The earliest documented plant collection was made by the Dutch botanist Georg Everhard Rumphius, who was employed by the Dutch East India Company (Verenigde Oost-Indische Compagnie, VOC) and arrived in Maluku in 1653 [40]. He wrote *Herbarium Amboinense*, a collection of plant specimens and descriptions that he compiled over several decades [40]. Later, Carl Peter Thunberg, a Swedish botanist and student of Linnaeus, was the first to undertake botanical exploration from Europe. He spent half a year in Java in 1777 collecting plants and published *Florula Javanica* in 1825 [41]. Other notable collectors include Claes Frederic Hornstedt (in 1783–1784), Francisco de Norona (in 1786), Louis Auguste Deschamps (in 1793–1802), Louis Theodore Leschenault de La Tour (in 1803–1806), and Thomas Horsfield (in 1802–1818) [42].

More systematic plant collection emerged during the 19th and 20th centuries. Caspar Georg Carl Reinwardt founded the Bogor Botanical Garden in 1817, marking the beginning of a new era of exploration [43]. Carl Ludwig Blume, Reinwardt’s successor, published *Catalogus van’s Lands Plantentuin* in 1823, listing more than 900 plant species, and described hundreds of new species in his *Bijdrage tot de kennis onzer Javaansche eiken* [44]. Friedrich Anton Wilhelm Miquel published *Flora Indiae Batavae* (1855–1859), which described the flora of the Malay Archipelago with illustrations, written partly in Latin and partly in Dutch [44]. Blume, Miquel, Zollinger, and Koorders attempted to write the Flora of the Netherlands Indies or the Flora of Java, and each of them published works for the region, but they were not comprehensive [12]. The Dutch botanist Cornelis Andries Backer completed the *Flora of Java*, which included over 6000 species of the spermatophytes belonging to 238 families and 2067 genera [45,46,47]. The Japanese botanist Genkei Masamune published a checklist of Bornean seed plants and ferns in 1942 and 1945, respectively, including 167 families, 1428 genera, and 8164 species [48,49]. The Dutch botanist Cornelis Gijsbert Gerrit Jan Steenis wrote the *Mountain Flora of Java* in 1972, with color drawings of 456 flowering plant species [50]. Airy Shaw listed the species of Euphorbiaceae in Borneo and New Guinea as 80 genera and 340 species and 48 genera and 500 species, respectively [51,52]. Whitmore et al. [53,54,55,56,57,58,59] published a series of books on the tree flora of Indonesia, including six checklists for the six regions of Indonesia except for Java, because the flora of Java already existed. Levang and de Foresta published 728 economic plants of Indonesia in Latin, Indonesian, French, and English [60].

In more recent decades, Indonesian botanists and researchers have taken a more active role in botanical studies. The Flora Malesiana (FM) project was initiated in the 1940s by van Steenis in Bogor, Indonesia, to undertake the cataloging of plants in the Malay Archipelago; the board of the foundation was chaired by an Indonesian representative from the Indonesian Institute of Sciences (Lembaga Ilmu Pengetahuan Indonesia, LIPI) [12]. Plant Resources of South East Asia (PROSEA), hosted by LIPI and funded by the government of the Netherlands, consists of 19 volumes on economic plants of Southeast Asia [12]. LIPI developed multiple biodiversity information systems, such as the Indonesian Biodiversity Information System (IBIS), the Indonesian National Biodiversity Information Network (NBIN), and the Indonesian Biodiversity Information Facility (InaBIF) [61]. Indonesian scientists published important work on botanical exploration and discovered many new species and taxa [62,63,64,65,66,67,68,69]. Priyadi et al. [70] published a guide to the plants in Gunung Halimun Salak National Park, which includes 500 species. *Flora of Bali: An Annotated Checklist* published by Girmansyah et al. [71] comprises a total of 1338 species of spermatophytes, 165 species of pteridophytes, 71 species of bryophytes and hepatics, and 75 species of fungi. Researchers from the LIPI Biological Research Center conducted several surveys on Wawonii Island in Southeast Sulawesi between 2003 and 2006, identifying that the island is home to no less than 1000 plant species [72]. LIPI researchers visited Enggano Island in the Sumatra region in 2015 to explore the island’s biodiversity in terms of ecosystems, flora and fauna, and microbial diversity and published the book *Ekspedisi Pulau Enggano* in 2017 [73]. *Ekspedisi Tambrauw: Sepotong Surga di Tanah Papua* [74] is a publication by LIPI researchers following an expedition to Tambrauw, West Papua, in 2016 and provides preliminary information on the potential of Tambrauw’s terrestrial ecosystems and biological resources. Mahyuni et al. [75] collected information about specimens from the Herbarium Bogoriense (BO) as well as many relevant publications to provide the first checklist of the flora of Lombok, which currently contains 1309 taxa. *Eksplorasi Flora: 25 Tahun Menjelajah Rimba Nusantara* published by Hidayat et al. [76] marks the 200th anniversary of the Bogor Botanical Garden and describes the explorations and contributions by LIPI scientists in Indonesia from 1991 to 2016. Mustaqim et al. [77] established a preliminary checklist website of Indonesian vascular plants, which includes 22,653 species. While this website may contain errors and incomplete information, it is an important step forward in botanical research in Indonesia. Important publications have also been published recently by international botanists, demonstrating their efforts on behalf of the region [78,79,80,81,82,83,84,85,86,87,88,89,90]. A checklist of the vascular flora of the Sunda–Sahul Convergence Zone, an area geographically larger than the Malay Archipelago, was published by Joyce et al. [89]. Ninety-nine botanists collaborated to provide the first checklist of the vascular plants of New Guinea, including 13,634 species, 1742 genera, and 264 families [90]. These works provide important references for carrying out research on a checklist of plants in Indonesia.

The Global Strategy for Plant Conservation (GSPC) 2011–2020 stated that a flora of all known plants is a fundamental requirement for plant conservation, and its objective I is that plant diversity is well understood, documented, and recognized [91]. However, despite many botanical studies and explorations conducted in Indonesia, there is a lack of a national checklist of plant species. In this paper, we aimed to fill this gap by systematically collecting and synthesizing botanical information from available sources related to Indonesian flora. We attempted to answer two questions: (1) how many native species of vascular plants are there in Indonesia? and (2) where are they distributed? Our study will enhance the understanding of plant diversity in Indonesia and provide basic data for research in biogeography and conservation biology.

## 2. Results

### 2.1. Floristic Composition in Indonesia

**Figure 2:**
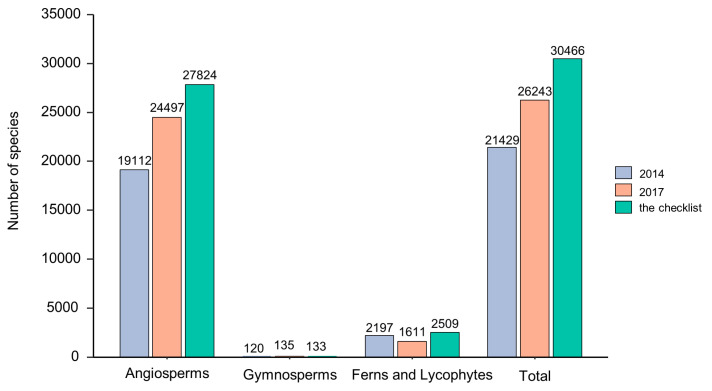
Comparison of the number of vascular plant species in this study with the number documented in 2014 and 2017, as mentioned in Indonesia’s sixth National Report to the CBD.

We identified 30,466 native species of vascular plants (including infraspecific taxa, the same below) in 317 families and 2968 genera (Data S1). The number of families, genera, and species in Indonesia accounted for 56%, 22%, and 8.7% of that of the world’s vascular plants, respectively. (See detailed numbers for angiosperms, gymnosperms, and ferns and lycophytes in Table 1.) Compared to the number of vascular plants mentioned in the sixth National Report of Indonesia to the Convention on Biological Diversity (CBD) [92], the number of angiosperm species and fern and lycophyte species is higher than documented in 2014, while the number of gymnosperm species is lower than that of 2017 (Figure 2).

**Figure 3:**
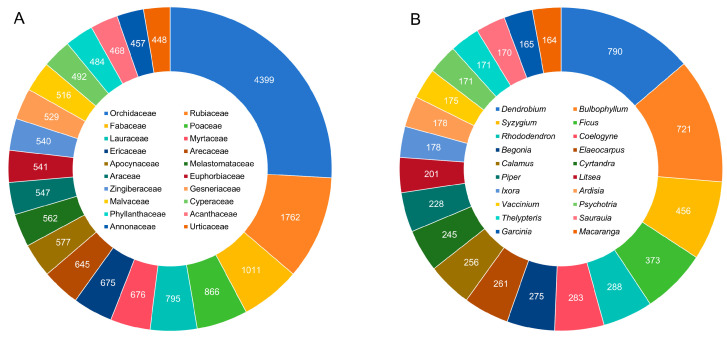
Top 20 species-rich families (**A**) and genera (**B**) of vascular plants in Indonesia.

We found that the number of species belonging to different families and genera varied considerably. Most families had fewer species, while fewer families had more species. In all 317 families, 15 families had more than 500 species, with a total of 14,641 species (48% of total flora) (Figure S1). The most diverse family is Orchidaceae, with 4399 species, accounting for 14% of the total flora, followed by Rubiaceae (1762), Fabaceae (1011), Poaceae (866), and Lauraceae (795) (Figure 3A). Of the 2968 genera, 113 genera had more than 50 species, with a total of 13,632 species (45%) (Figure S1). The five largest genera are *Dendrobium* (790 species) of Orchidaceae, *Bulbophyllum* (721 species) of Orchidaceae, *Syzygium* (456 species) of Myrtaceae, *Ficus* (373 species) of Moraceae, and *Rhododendron* (288 species) of Ericaceae (Figure 3B).

### 2.2. Number of Species of Vascular Plants and Similarity Analysis of the Seven Regions of Indonesia

We identified 29,308 species (96% of the total) at the regional level, belonging to 314 families and 2904 genera. Sumatra had the highest number of species (10,902), followed by Indonesian New Guinea (9935), Java (9289), and Kalimantan (9191). The percentage of locally endemic species in Indonesian New Guinea was remarkably high (59%), followed by Kalimantan (38%) (Table 2). We counted the top five species-rich families in each region and found a total of 19 families were responsible for the most common species; Orchidaceae had the highest number of species in all regions (Figure S2).

**Figure 4:**
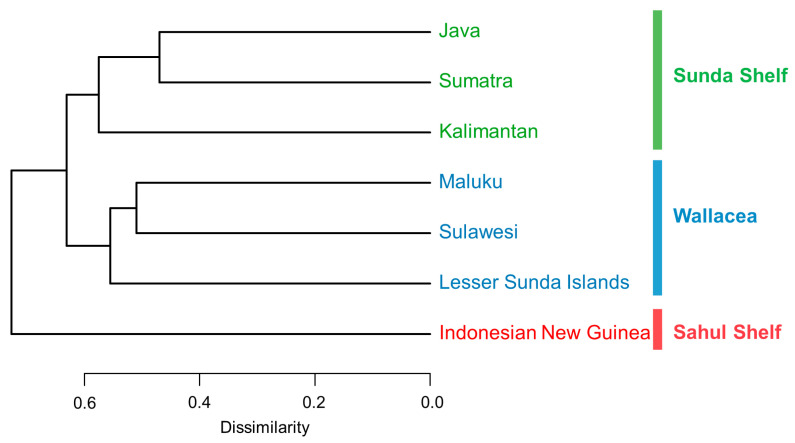
Dendrogram of the seven regions based on UPGMA hierarchical clustering.

Sumatra and Java shared 5378 species and had the highest species similarity coefficient of 0.53. Species similarity was also high between Sumatra and Kalimantan (0.48) and between Sulawesi and Maluku or Lesser Sunda Islands (0.49 and 0.45, respectively, Table S1). In the clustering tree, Sumatra and Java were clustered together and most closely related, and then merged with Kalimantan. Similarly, Sulawesi, Maluku, and Lesser Sunda Islands were grouped separately. Indonesian New Guinea had a low number of plants in common with the other six regions (species similarity coefficient of 0.3 with Sulawesi, 0.37 with Maluku, and less than 0.3 with other regions) and was classified as a separate branch. These seven regions were divided into three phytogeographical areas, which are the Sunda Shelf, the Sahul Shelf, and the Wallacea [94] (Figure 4).

## 3. Discussion

### 3.1. Number of Vascular Plant Species in Indonesia

Our study is the first attempt to synthesize botanical information about the flora of Indonesia from various available sources, presenting the first comprehensive species checklist of native vascular plants for Indonesia. Based on our synthesis, we recorded 30,466 native vascular plants in Indonesia. Our number was higher than the number of vascular plants in the sixth National Report of Indonesia to the CBD. It should be mentioned that the number of species mentioned in the report does not have a corresponding publicly available checklist, so we are unable to make a detailed comparison between the two or three checklists. We consider our result conservative because we removed undetailed distribution records in Borneo, New Guinea, and Timor, because these records might have been recorded in other countries on these islands rather than in Indonesia. However, we have to recognize that due to Indonesia’s unique geographic location, it is necessary to cooperate with botanists from neighboring countries (e.g., Malaysia, Papua New Guinea, Brunei, and Timor-Leste) to obtain a more authoritative checklist of Indonesia. Because species ranges may cross political borders, there is a strong need for cross-border cooperation in both species surveys and conservation [95,96,97]. The total number of species is a temporary figure because some species will be added or removed as new species are discovered and taxa are revised. Nevertheless, the first checklist we presented here will be valuable in understanding Indonesia’s plant diversity and assisting its conservation planning.

Completing the documentation is the most fundamental step for conservation and research, and it is a long-term process requiring persistent updating and revision. Indonesia has a land area of 1.9 million km^2^, but the number of vascular plants is among the highest in the world, perhaps ranking third in the world, after Brazil and China [98,99]. The number of new species of vascular plants published annually in China and Brazil is averaged to about 200 per year [100,101]. During the past 20 years, an average of 76 new species have been discovered per year in Indonesia, according to the International Plant Names Index (http://www.ipni.org, accessed on 14 July 2023) (Figure S3). There is great potential to discover new species in Indonesia.

### 3.2. Distribution Patterns of Vascular Plants in Indonesia

Among the seven regions or whole islands, the plant checklists for Java, *Flora of Java* [45,46,47], and for Borneo, *Enumeratio Phanerogamarum Bornearum* and *Enumeratio Pteridophytarum Bornearum* [48,49], are over 50 years old. Only New Guinea has the latest published checklist of vascular plants [90], and we included it in our database. The number of species predicted for the islands is available in earlier studies; Borneo has the highest species richness, followed by New Guinea and Sumatra [94,102], with these three islands having greater land surface area. Similar to previous predictions, we found larger regions had more plants, with Sumatra having the highest number of species, followed by Indonesian New Guinea and Kalimantan. The high number of plant species in Java is possibly due to the long period of plant research on the island, both during the Dutch colonial period and nowadays [92]. We found that Indonesian New Guinea and Kalimantan had the highest percentage of locally endemic species, consistent with previous studies that show New Guinea has the highest proportion of endemic species, followed by Borneo [94,102]. The very high percentage of locally endemic species (59%) resulted in low similarity between the flora of Indonesian New Guinea and other regions. The uniqueness of New Guinea may be due to its Australian origin, great land area, and habitat diversity [90]. Borneo was a hotspot of rapid divergence of angiosperms and ferns in the early Miocene (ca. 20 Ma) [18], resulting in high endemism.

Our study grouped the seven regions into three phytogeographical areas (the Sunda Shelf, the Sahul Shelf, and the Wallacea) based on similarities in floristic composition as shown in Figure 4, similar to the phytogeographical areas identified in the flora of the Malay Archipelago [103]. There is an ongoing debate about which phytogeographical area Java belongs to [104]. Since Wallace’s time, Java has been part of the Sunda region along with Sumatra and Kalimantan, according to zoological data [104]. However, van Welzen et al. [103] examined 7340 plant species from the Malay Archipelago and concluded that Java should be east of the Wallace Line and part of the Wallacea. Our study supported Java belonging to the Sunda Shelf. The unique climate of Java, with the humid west similar to the Sunda Shelf and the dry east closer to the Wallacea, may lead to different species compositions in the east and west [105]. Our analysis treated Java as a whole, and differences in the number of species in the east and west may affect the delineation of phytogeographical areas, especially if the collection was uneven between the east and west. The similarity coefficient between Java and Lesser Sunda Islands is also high (0.5), possibly due to the historical connection between Java and Bali of Lesser Sunda Islands during glacial maxima, facilitating species movement between Java and Lesser Sunda Islands [103]. We recorded the least species in Lesser Sunda Islands and Maluku. In the middle of the Miocene Epoch (ca. 25 Ma), the Australian plate collided with the Sunda plate, raising a number of islands to sea level and forming what is now known as the “Wallacea” [106]. These small islands serve as stepping stones for species dispersal [103] and are also small in size with fewer plants on them.

## 4. Future Directions

We have recorded 30,466 native vascular plants in Indonesia, and we expect a higher number of species when more information from local areas is integrated and more new species are discovered. We suggest that work on the taxonomy of Indonesian plants should be strengthened, such as through specimen identification and more collections and taxonomic revision of important families and genera. Despite the increasing involvement of Indonesian botanists, their input is very limited due to lack of sufficient funding for taxonomy [107,108]. The number of taxonomists is declining [109] and investing in the development and training of taxonomists is crucial. Without sufficient financial backing, it becomes challenging for researchers to carry out the long-term and basic work like taxonomy.

In recent decades, Indonesia’s biodiversity has been seriously threatened due to the destruction of large tracts of primary forests and peatlands, resulting in many species being at risk due to habitat loss [110,111]. Checklist data is the basis for conservation and has proven to be an important source of baseline information for both scientists and policymakers [3,112]. Undiscovered species will have a higher risk of extinction than known species [113]. It will be necessary to figure out what species are already present and to carry out more botanical exploration to discover new species in Indonesia. International cooperation is very important to promote the study of Indonesian plants; for example, Flora Malesiana was initiated by the Dutch [12], Mapping Asia Plants was initiated by the Chinese Academy of Sciences [4], and the newly published plant checklists of New Guinea and Sunda–Sahul were conducted by foreign researchers [89,90]. Indonesia’s largest herbarium, the Bogor Herbarium, has a collection of more than one million specimens, but the digitization process is slow and not yet complete. There is an urgent need to strengthen international collaboration and increase financial support to promote the digitization of specimens in Indonesia. This would not only preserve the invaluable knowledge these specimens hold but also facilitate crucial basic research, such as species documentation and threat assessments.

## 5. Materials and Methods

### 5.1. Data Compilation

We collected vascular plant data for Indonesia from online specimens, online checklists, and the literature (Data S2), obtaining a total of 1.62 million data entries. We collected names of species, names of countries and regions (i.e., Java, Sumatra, Kalimantan, Maluku, Lesser Sunda Islands, Sulawesi, and Indonesian New Guinea) (Figure 1), and information on the distribution of the species. We removed records with less detailed information from Borneo, Timor, and New Guinea because it was not possible to determine whether the species belonged to Indonesia. We mapped records with coordinate points to the administrative division shapefiles (http://www.gadm.org/, accessed on 14 July 2023) to obtain the region information for these points. All collection data were current as of March 2022.

We excluded fungi, lichens, bryophytes, and doubtful species names (e.g., ‘cf.’, ‘sp.’, ‘aff.’, ‘spp.’, ‘indet.’). The taxonomic status and accepted names of species from all data sources were standardized following the Leipzig Catalogue of Vascular Plants (LCVP, version 1.0.3) [93], World Checklist of Vascular Plants (WCVP, http://wcvp.science.kew.org/, accessed on 30 March 2024), World Plants (WP, https://www.worldplants.de/, accessed on 26 June 2023), and World Flora Online (WFO, http://www.worldfloraonline.org/, accessed on 24 March 2024) in U. Taxonstand with fuzzy matching [114]. We considered the highest level of confidence when three or four databases agreed on the species, so we kept these species. Synonyms were replaced with the accepted names. We used the family classification system of the APG IV [115] for angiosperms, that of Christenhusz et al. [116] for gymnosperms, and that of the PPG I [117] for ferns and lycophytes. We focused on native species and referred to *A dataset on catalogue of alien plants in China* [118], *A Guide Book to Invasive Alien Plant Species in Indonesia* [119], *The naturalized vascular flora of Malesia* [120], *Global Register of Introduced and Invasive Species-Indonesia* [121], and Query World Economic Plants in GRIN-Global (https://grin.devlab.cz/gringlobal/taxon/taxonomysearchwep.aspx, accessed on 21 May 2023) to exclude species cultivated, introduced, and naturalized in Indonesia. We also used keywords (e.g., ‘cultivated’, ‘botanical garden’, ‘campus’, ‘planting’) in distribution information to exclude non-native species. After completing the above steps, there were 1.18 million data entries in the database.

### 5.2. Data Analysis

We generated the vascular plant species list of Indonesia based on our database and compared the number of species with the global number of vascular plants based on the LCVP database [93] and the number obtained in 2014 and 2017 mentioned in the sixth National Report of Indonesia to the CBD [92]. In this study, we used the term “locally endemic” for species that only occurred in a region within Indonesia and not in a global sense. We counted the total number of species and the number of locally endemic species in each region and examined species similarities between regions using Sørensen’s similarity coefficient. The calculation formula is S=2j/(a+b), where a and b are the number of species in two regions and j is the number of shared species in the two regions [122]. The coefficient ranges from 0 to 1, with higher values indicating higher plant similarity between the two regions. Unweighted Pair Group Method with Arithmetic Mean (UPGMA) is a widely used clustering method for constructing cluster trees or evolutionary trees [123,124,125,126]. UPGMA is based on the principle of distance minimization, where the two most similar samples are merged by calculating the average distance between them and then considered as a new cluster; this process is repeated until all samples are merged into one cluster [123]. We performed a hierarchical clustering using the UPGMA in R 4.2.2 to obtain a dendrogram that identifies nested relatedness among regions.

## Figures and Tables

**Table 1:** Composition of plant diversity in Indonesia and its proportion in the world.

	Angiosperms	Gymnosperms	Ferns and Lycophytes	Total	Number of Vascular Plants Globally	Percentage
Family	269	7	41	317	564	56%
Genus	2782	17	169	2968	13,460	22%
Species	27,824	133	2509	30,466	351,180	8.7%

Note: The global plant data is based on the Leipzig Catalogue of Vascular Plants [93]. For ferns and lycophytes, the number of fern species is 2285, which is 91% of the total number of this group.

**Table 2:** Number of vascular plants in the seven regions of Indonesia.

Region	Region Size (km^2^)	Family	Genus	Species	Locally Endemic Species	
					Number	Percentage
Sumatra	480,793	270	1862	10,902	3358	31%
Kalimantan	544,151	238	1534	9191	3498	38%
Java	129,399	274	2141	9289	2405	26%
Lesser Sunda Islands	73,070	238	1400	4514	554	12%
Sulawesi	188,522	253	1565	7048	1771	25%
Maluku	78,897	234	1437	5294	778	15%
Indonesian New Guinea	421,982	254	1601	9935	5901	59%

Note: the region size is from http://www.xzqh.org/old/waiguo/asia/1015.htm (accessed on 29 June 2023).

## Data Availability

The data presented in this study are available in Data S1.

## Supplementary Materials

The following supporting information can be downloaded at: https://www.mdpi.com/article/10.3390/plants13162281/s1, Figure S1: Number of families and genera versus number of species; Figure S2: The top five families of total number of species and number of locally endemic species in each region; Figure S3: Increase in the number of new species of vascular plants published in Indonesia between 2000 and 2022; Table S1: Similarity and statistics analysis of species in seven regions; Data S1: Checklist of the vascular plants in Indonesia; Data S2: List of data sources.
