# Supplementary material for: Mapping Asia Plants: Plant Diversity and a Checklist of Vascular Plants in Indonesia
Source: Plants (Basel). 2024 Aug 16;13(16):2281. doi: 10.3390/plants13162281 (PMC11360604; doi:10.3390/plants13162281)
Supplement: Supplementary file 1 [file plants-13-02281-s001.zip › Data S2 List of data sources.pdf]

Appendix List of data sources

Data sources online

1. Botanical Information and Ecology Network (http://bien.nceas.ucsb.edu/bien/)
2. Flora of China (http://www.efloras.org/)
3. Global Tree Search (https://tools.bgci.org/global_tree_search.php)
4. Plants of the World Online (https://powo.science.kew.org/)
5. Digital flora of Indonesia (https://www.indonesiaplants.org/)
6. IUCN (https://www.iucnredlist.org/en)
7. Catalogue of Life (https://www.catalogueoflife.org/)
8. Indonesian Biodiversity Information System (http://www.biologi.lipi.go.id)
9. Useful Tropicos Plants (https://tropical.theferns.info/)
10. Terrestrial Biozones (https://www.terrestrial-biozones.net/)
11. Ferns of Sulawesi (https://sulawesiferns.myspecies.info/)
12. Global Compositae Checklist (https://www.compositae.org/gcd/index.php)
13. ILDIS Legumes of the World (http://www.ildis.org/AliceWeb/6.00/)
14. Checklist of Sulawesi Begonia (http://portal.cybertaxonomy.org/ flora-malesiana- prospective/node/95)
15. Plants of Southeast Asia (https://www.asianplant.net/)
16. Global Biodiversity Information Facility (https://www.gbif.org/)
17. Royal Botanic Gardens, Kew (https://apps.kew.org/herbcat/navigator.do)
18. Tropicos (https://www.tropicos.org/collection/Search)
19. Singapore herbarium online (https://herbaria.plants.ox.ac.uk/bol/sing)
20. Smithsonia national museum (https://collections.nmnh.si.edu/search/botany/)
21. Royal Botanic Garden, Edinburgh (https://www.rbge.org.uk/)
22. Harvard University Herbaria (https://kiki.huh.harvard.edu/databases/)
23. New York Botanical Garden (https://www.nybg.org/)
24. Herbarium Wu (https://herbarium.univie.ac.at/)
25. Botanic Garden Meise (https://www.plantentuinmeise.be/en/)
26. IDiGBio (https://www.idigbio.org/)
27. Herbarium Berolinense (https://www.bgbm.org/en)
28. iNaturalist (https://www.inaturalist.org/)
29. Berkeley Natural History Museums (https://bnhm.berkeley.edu/our-museums/ucjeps/)
30. Genesys (https://www.genesys-pgr.org/)

31. International Plant Names Index (https://www.ipni.org)

Data sources from books and papers

1. Richard SJ, Suryadi S. A biodiversity assessment of Yongsu-Cyclops mountains and the southern Mamberamo basin, Papua, Indonesia. RAP Bulletin of Biological Assessment 25[M]. Washington: CI, 2002.
2. Gibbs LS. A contribution to the phytogeography and flora of the Arfak mountains[M]. London: Taylor and Francis, 1917.
3. Coode MJE. Manual of the Forest Trees of Papua and New Guinea[M]. Lae: Division of Botany, Department of Forests, 1969.
4. Johns RJ, Edwards PJ, Utteridge TMA, et al. A Guide to the Alpine and Subalpine Flora of Mount Jaya[M]. London: Royal Botanic Gardens, Kew, 2006.
5. Utteridge TMA, Jennings LVS. Trees of New Guinea[M]. London: Royal Botanic Gardens, Kew, 2021.
6. Nugroho H, Riyanto A, Wiantoro S, et al. Ekspedisi Tambrauw: Sepotong surga di tanah Papua[M]. Jakarta: LIPI Press, 2019.
7. Airy Shaw HK. The Euphorbiaceae of New Guinea[M]. London: Royal Botanic Gardens, Kew, 1980.
8. Womersley JS. Handbooks of the Flora of Papua New Guinea Volume I[M]. Victoria: Melbourne University Press, 1978.
9. Henty EE. Handbooks of the Flora of Papua New Guinea Volume II[M]. Victoria: Melbourne University Press, 1981.
10. Conn BJ. Handbooks of the Flora of Papua New Guinea Volume III[M]. Victoria: Melbourne University Press, 1995.
11. van Royen P. The Orchids of the High Mountains of New Guinea[M]. Germany: A.R.Gantner Verlag Kommanditgesellschaft, 1980.
12. Schuiteman A. A Guide to Dendrobium of New Guinea[M]. Kinabalu: Natural History Publications (Borneo), 2013.
13. Verdcourt B. A Manual of New Guinea Legumes[M]. Lae: Office of Forests, Division of Botany, 1979.
14. Whitmore TC, Tantra IGM, Sutisna U. Tree flora of Indonesia: check list for Sumatra[M]. Bogor: Forest Research and Development Centre, 1986.
15. Whitmore TC, Tantra IGM, Sutisna U. Tree Flora of Indonesia: check list for Maluku[M]. Bogor: Forest Research and Development Centre, 1989.
16. Whitmore TC, Tantra IGM, Sutisna U. Tree Flora of Indonesia: check list for Bali, Nusa Tenggara and Timor[M]. Bogor: Forest Research and Development Centre,

1989.
17. Whitmore TC, Tantra IGM, Sutisna U. Tree Flora of Indonesia: check list for Sulawesi[M]. Bogor: Forest Research and Development Centre, 1989.
18. Whitmore TC, Tantra IGM, Sutisna U. Tree flora of Indonesia: check list for Kalimantan Part I[M]. Bogor: Forest Research and Development Centre, 1990.
19. Whitmore TC, Tantra IGM, Sutisna U. Tree flora of Indonesia: check list for Kalimantan Part II[M]. Bogor: Forest Research and Development Centre, 1990.
20. Smith JJ. Die Orchideen von Ambon[M]. Batavia: Landsdrukkerij, 1905.
21. Backer CA, Bakhuizen van de Brink Jr., RC. Flora of Java, vol 1[M]. Groningen: Wolters and Noordhoff, 1963.
22. Backer CA, Bakhuizen van de Brink Jr., RC. Flora of Java, vol 2[M]. Groningen: Wolters and Noordhoff, 1965.
23. Backer CA, Bakhuizen van de Brink Jr., RC. Flora of Java, vol 3[M]. Groningen: Wolters and Noordhoff, 1968.
24. Priyadi H, Takao G, Rahmawati I, et al. Five hundred plant species in Gunung Halimun Salak National Park, West Java: a checklist including Sundanese names, distribution and use[M]. Bogor: Center for International Forestry Research, 2010.
25. Mahyuni R, Mansur M, Rahayu M, et al. Checklist Flora of Lombok[M]. Bogor: Herbarium Bogoriense, Research Center for Biology, Indonesian Institute of Sciences, 2020.
26. Maryanto I, Hamidy A, Keim AP, et al. Ekspedisi Pulau Enggano[M]. Jakarta: LIPI Press, 2017.
27. Masamune G. Enumeratio Phanerogamarum Bornearum[M]. Japan: Taihoku Imperial University, 1942.
28. Masamune G. Enumeratio Pteridophytarum Bornearum[M]. Japan: Taihoku Imperial University, 1945.
29. Rugayah, Sunarti S, Sulistiarini D, et al. Daftar Jenis Tumbuhan di Palau Wawonii, Sulawesi Tenggara[M]. Jakarta: LIPI Press, 2015.
30. van Steenis CGGJ. The Mountain Flora of Java[M]. Leiden: Brill, 1972.
31. Girmansyah D, Santika Y, Retnowati A, et al. Flora of Bali: An Annotated Checklist[M]. Bogor: Research Center for Biology Indonesian Institute of Sciences-LIPI, 2013.
32. Lianah. Biodiversitas Zingiberaceae Mijen Kota Semarang[M]. Yogyakarta: Deepublish, 2020.
33. Backer CA. Flora van Batavia[M]. Batavia: G. Kolff, 1907.

34. Rindyastuti R, Abywijaya IK, Rahadiantoro A, et al. Keanekaragaman Tumbuhan Pulau Sempu dan Ekosistemnya[M]. Jakarta: LIPI Press, 2018.
35. Koorders SH. Exkursionsflora von Java Umfassend die Blütenpflanzen[M]. Jena: Verlag von Gustav Fischer, 1913.
36. Koorders SH. Flora von Tjibodas umfassend die Blütenpflanzen[M]. Batavia: Verlag von N. V. Boekhandel Visser & Co, 1918.
37. Partomihardjo T, Arifiani D, Pratama BA. Jenis-Jenis Pohon Penting di Hutan Nusakambangan[M]. Jakarta: LIPI Press, 2014.
38. Comber JB. Orchids of Java[M]. London: Royal Botanic Gardens, Kew, 1990.
39. Keßler PJA, Bos MM, Sierra Daza SEC, et al. Checklist of woody plants of Sulawesi, Indonesia[J]. Blumea Supplement, 2002, 14: 1-160.
40. Achmadi AS, Hamidy A, Maryanto I. Ekspedisi Sulawesi Barat: Flora, Fauna, dan Mikroorganisme Gandangdewata[M]. Jakarta: LIPI Press, 2018.
41. Rugayah, Sunarti S, Sulistiarini D, et al. Daftar Jenis Tumbuhan di Palau Wawonii, Sulawesi Tenggara[M]. Jakarta: LIPI Press, 2015.
42. Ardiyani M, Clayton L, Hidayat A. A Picture Guide to the Plants of the Nantu Wildlife Sanctuary[M]. Jakarta: LIPI Press, 2020.
43. Vermeulen J, O'Byrne P. Bulbophyllum of Sulawesi[M]. Kinabalu: Natural History Publications (Borneo), 2011.
44. Poulsen AD. Etlingera of Sulawesi[M]. Kinabalu: Natural History Publications (Borneo), 2012.
45. Pitopang R, Lapandjang Ir. I, Burhanuddin IF. Profil Herbarium Celebense Universitas Tadulako dan Deskripsi 100 Jenis Pohon Khas Sulawesi[M]. Kota Palu: Herbarium Celebense UNTAD, 2011.
46. Rugayah, Rahayu M, Mulyadi, et al. Pulau Wawonii: Keanekaragaman Ekosistem, Flora, dan Fauna[M]. Jakarta: LIPI Press, 2019.
47. Handoyo F, Prasetya R. Orchids of Sulawesi[M]. Jakarta: Indonesian Orchid Society, 2012.
48. Merrill ED. An enumeration of plants collected in Sumatra by W.N. and C.M.Bangham[M]. Boston: The Arnold Arboretum of Harvard University, 1934.
49. Clarke C. Nepenthes of Sumatra and Peninsular Malaysia[M]. Kinabalu: Natural History Publications (Borneo), 2001.
50. Partomihardjo T, Hermawan E, Pradana EW, et al. Flora Riparian dan Hutan Rawa Gambut untuk Restorasi Area dengan Nilai Konservasi Tinggi (NKT) Terdegradasi[M]. Indonesia: Zoological Society of London (ZSL), 2020.

51. Munawaroh E, Yuzammi, Solihah SM, et al. Koleksi Kebun Raya Liwa, Lampung: Tumbuhan Berpotensi sebagai Tanaman Hias[M]. Jakarta: LIPI Press, 2017.
52. Miquel FAW. Sumatra, zijne plantenwereld en hare voortbrengselen[M]. Amsterdam: C.G. van der Post, 1862.
53. Comber JB. 2001. Orchids of Sumatra[M]. London: Royal Botanic Gardens, Kew, 2001.
54. Merrill ED. A bibliographic enumeration of Bornean plants[M]. Singapore: Fraser & Neave, ltd, 1921.
55. Wood JJ, Cribb PJ. A Check-list of the Orchids of Borneo[M]. London: Royal Botanic Gardens, Kew, 1994.
56. Kiew R, Sang J, Repin R, et al. A Guide to Begonias of Borneo[M]. Kinabalu: Natural History Publications (Borneo), 2015.
57. Vermeulen J, O'Byrne P. Bulbophyllum of Borneo[M]. Kinabalu: Natural History Publications (Borneo), 2015.
58. Witono R, Joko, Rustiami H, et al. 2013. Panduan Lapangan Pengenalan Jenis Rotan Katingan[M]. Palangka Raya: WWF-Indonesia Program Kalimantan Tengah, 2013.
59. Argent G, Saridan A, Campbell EJF, et al. Manuals of Larger and More Important Non Dipterocarp Trees of Central Kalimantan, Indonesia[M]. Samarinda: Forest Research Institute, 1997.
60. Clarke C. Nepenthes of Borneo[M]. Kinabalu: Natural History Publications (Borneo), 1997.
61. Cribb P. Slipper Orchids of Borneo[M]. Kinabalu: Natural History Publications (Borneo), 1997.
62. Keßler PJA, Sidiyasa K, Ambriansyah, et al. Checklist for a tree flora of the Balikpapan-Samarinda area, East Kalimantan, Indonesia[M]. Wageningen: The Tropenbos Foundation, 1992.
63. Airy Shaw HK. The Euphorbiaceae of Borneo[M]. London: Royal Botanic Gardens, Kew, 1975.
64. Keßler PJA, Sidiyasa K. Trees of the Balikpapan-Samarinda Area, East Kalimantan, Indonesia. A manual to 280 selected species[M]. Wageningen: The Tropenbos Foundation, 1994.
65. Keßler PJA, Sidiyasa K, Ambriansyah, et al. Checklist of Secondary Forest Trees in East and South Kalimantan, Indonesia[M]. Wageningen: The Tropenbos

Foundation, 1995.
66. Lamb A, Gobilik J, Ardiyani M, et al. A guide to Gingers of Borneo[M]. Kinabalu: Natural History Publications (Borneo), 2013.
67. Lamb A, Rodda M. A guide to Hoyas of Borneo[M]. Kinabalu: Natural History Publications (Borneo), 2016.
68. Wood JJ. Dendrobium of Borneo[M]. Kinabalu: Natural History Publications (Borneo), 2014.
69. Wood JJ. Dendrochilum of Borneo[M]. Kinabalu: Natural History Publications (Borneo), 2001.
70. Poulsen AD. Etlingera of Borneo[M]. Kinabalu: Natural History Publications (Borneo), 2006.
71. Wong KM. The Genus Melastoma in Borneo[M]. Kinabalu: Natural History Publications (Borneo), 2016.
72. Arinasa IBK, Adjie B, Putri DMS, et al. An Alphabetical List of Plant Species Cultivated in Bali Botanic Garden[M]. Jakarta: LIPI Press, 2017.
73. Sujarwo W, van der Hoeven B, Pendit IMR, et al. Traditional Balinese Medicinal Plants[M]. Jakarta: LIPI Press, 2020.
74. Arinasa IBK, Peneng IN. Jenis-Jenis Bambu di Bali dan Potensinya[M]. Jakarta: LIPI Press, 2014.
75. Dharma IDP, Solihah SM, Kuswantoro F, et al. Koleksi Kebun Raya Lombok: Tumbuhan Sunda Kecil[M]. Jakarta: LIPI Press, 2017.
76. Kurniawan A, Asih NPS. Araceae di Pulau Bali[M]. Jakarta: LIPI Press, 2012.
77. Kusumanegara A, Pribadi EY, Jannah AM, et al. Menyingkap Rahasia Jenis-Jenis Tumbuhan Obat di Taman Nasional Matalawa Sumba-Nusa Tenggara Timur[M]. Nusa Tenggara Timur: Balai Taman Nasional Manupeu Tanah Daru dan Laiwangi Wanggameti, 2020.
78. Hamidy A, Witjaksono, Sihotang, VBL. Ekspedisi Sumba[M]. Jakarta: LIPI Press,2017.
79. Friedberg C. Le savoir botanique des Bunaq: percevoir et classer dans le Haut Lamaknen (Timor, Indonésie)[M]. Paris: Editions du Museum, 1990.
80. Handoyo F. Orchids of Indonesia, vol 1[M]. Jakarta: Indonesian Orchid Society, 2010.
81. Levang P, de Foresta H. Economic Plants of Indonesia: A Latin, Indonesian, French and English Dictionary of 728 species[M]. Bogor: Orstom and SEAMEO BIOTROP, Southeast Asian Regional Centre for Tropical Biology, 1991.

82. Hughes M. An Annotated Checklist of Southeast Asian Begonia[M]. Edinburgh: Royal Botanic Gardens Edinburgh, 2008.
83. Ernst A. The New Flora of the Volcanic Island of Krakatau[M]. Cambridge: Cambridge University Press, 1908.
84. Handayani T, Wawangningrum H, Wihermanto, et al. Identifikasi Semai Tumbuhan Berkayu[M]. Jakarta: LIPI Press, 2017.
85. Hidayat S, Puspitaningtyas DM, Hartini S, et al. Eksplorasi Flora: 25 Tahun Menjelajah Rimba Nusantara[M]. Jakarta: LIPI Press, 2017.
86. Heyne K. De nuttige planten van Nederlandsch-Indië, tevens synthetische catalogus der verzamelingen van het Museum voor Economische Botanie te Buitenzorg, Deel II[M]. Batavia: Ruygrok & Co, 1916.
87. Heyne K. De nuttige planten van Nederlandsch-Indië, tevens synthetische catalogus der verzamelingen van het Museum voor Economische Botanie te Buitenzorg, Deel III[M]. Batavia: Ruygrok & Co, 1917.
88. Heyne K. De nuttige planten van Nederlandsch-Indië, tevens synthetische catalogus der verzamelingen van het Museum voor Economische Botanie te Buitenzorg, Deel IV[M]. Batavia: Ruygrok & Co, 1917.
89. Heyne K. De nuttige planten van Nederlandsch-Indië, tevens synthetische catalogus der verzamelingen van het Museum voor Economische Botanie te Buitenzorg, Deel I[M]. Batavia: Ruygrok & Co, 1922.
90. Hamidi A, Yulita KS, Kalima T, et al. Strategi Konservasi 12 Spesies Pohon Prioritas Nasional 2019-2029[M]. Jakarta: LIPI Press, 2019.
91. Ardiyani M, Dwibadra D, Dewi K, et al. Temuan dan Pertelaan Jenis Baru Biota Indonesia 1967-2017: Sumbangsih LIPI untuk Sains[M]. Jakarta: LIPI Press, 2017.
92. Widyatmoko D, Zich F. The Flora of Bukit Tigapuluh National Park, Kerumutan Sanctuary and Mahato Protective Reserve, Riau, Indonesia[M]. Jakarta: Indonesian Botanic Gardens and Yayasan Sosial Chevron dan Texaco Indonesia, 1998.
93. Sutrisno H, Afriani D, Rahmadi C, et al. Penemuan Jenis Baru Lembaga Ilmu Pengetahuan Indonesia Tahun 2010-2014[M]. Jakarta: LIPI Press, 2015.
94. Suprapto A, Solihah SM, Yuzammi, et al. Koleksi Kebun Raya Pucak Tumbuhan Bernilai Ekonomi[M]. Jakarta: LIPI Press, 2016.
95. Dodo, Solihah SM, Yuzammi. Koleksi Kebun Raya Banua: Tumbuhan Berpotensi Obat[M]. Jakarta: LIPI Press, 2016.

96. Witono JR, Yuzammi. Koleksi Tumbuhan Buah Kebun Raya Katingan[M]. Jakarta: LIPI Press, 2017.
97. van Steenis GGGJ. Flora voor de scholen in Indonesie[M]. Djakarta: Noordhoff-Kolff N.V., 1951.
98. Mogea JP, Gandawidjaja D, Wiriadinata H, et al. Tumbuhan Langka Indonesia[M]. Bogor: Biologi-LIPI, 2001.
99. Rugayah, Yulita KS, Arifiani D, et al. Tumbuhan Langka Indonesia: 50 Jenis Tumbuhan Terancam Punah[M]. Jakarta: LIPI Press, 2017.
100. Latifah D, Sudarmono. Flora Anemokori Hijaukan Bumi[M]. Jakarta: LIPI Press, 2021.
101. Wijayakusuma HMH, Dalimartha S, Wirian AS, et al. Tanaman Berkhasiat Obat di Indonesia Jilid I[M]. Jakarta: Pustaka Kartini, 1992.
102. Wijayakusuma HMH, Dalimartha S, Wirian AS, et al. Tanaman Berkhasiat Obat di Indonesia Jilid II[M]. Jakarta: Pustaka Kartini, 1993.
103. Wijayakusuma HMH, Dalimartha S, Wirian AS, et al. Tanaman Berkhasiat Obat di Indonesia Jilid III[M]. Jakarta: Pustaka Kartini, 1994.
104. Blume CL. Bijdragen tot de flora van Nederlandsch Indië[M]. Batavia: Ter lands drukkerij, 1825.
105. Wati RK, Mursidawati S. Orchidaceae Catalogue of Bogor Botanic Gardens[M]. Jakarta: LIPI Press, 2015.
106. Sudarmonowati E, Yulita KS, Partomihardjo T, et al. Daftar Merah Tumbuhan Indonesia 1: 50 Jenis Pohon Kayu Komersial[M]. Jakarta: LIPI Press, 2020.
107. Danser BH. The Nepenthaceae of the Netherlands Indies[M]. Kinabalu: Natural History Publications (Borneo), 2006.
108. Koster JH. The Compositae of the Malay Archipelago 1. Vernonieae and Eupatorieae[M]. Leiden: N.V. Boek-En Steendrukkerij Eduard Ijdo, 1935.
109. Lam HJ. The Verbenaceae of the Malayan Archipelago, together with those from the Malayan Peninsula,the Philippines, the Bismark-Archipelago, and the Palau, Marianne and Caroline-Islands[M]. Groningen: M. de Waal, 1919.
110. Miquel FAW. Flora van Netherlandsch Indie[M]. Amsterdam: C. G. van der Post, 1855-1860.
111. Moon HK, Ujang SI, Park SY, et al. Tropical trees of Indonesia-A Field Guide to Tropical Trees[M]. Seoul: Korea Forest Research Institute, 2011.
112. Keßler PJA. 2000. A field Guide to the important tree species of the Berau region[M]. Berau: Berau Forest Management Project, 2000.

113. Christ H. Die Farnflora von Celebes[M]. Leyde: E.J. Brill, 1898.
114. Christ H. Zur Farnflora von Celebes[M]. Leyde: E.J. Brill, 1904.
115. Koorders SH, Valeton T. Bijdragen No. 1-13 Tot De Kennis der Boomsoorten Op Java[M]. Batavia: G. Kolff & co, 1894-1914.
116. van Steenis GGGJ, van Steenis-Kruseman MJ. Flora Malesiana Series I Spermatophyta[M]. Djakarta: Noordhoff-Kolff, 1948-2016.
117. Holttum RE, van Steenis GGGJ. Flora Malesiana Series II Pteridophyta[M]. Boston: M. Nijhoff/W. Junk, 1959-2012.
118. Cosiaux A. Local uses of tree species and contribution of mixed tree gardens to livelihoods in Saleman: Village near Manusela National Park, Seram Island, Maluku (Indonesia)[M]. Bogor: Center for International Forestry Research, 2014.
119. Gillison AN, Liswanti N, Rachman IA. Rapid Ecological Assessment Kerinci Seblat National Park Buffer Zone[M]. Bogor: Center for International Forestry Research, 1996.
120. Dransfield S. Notes on Schizostachyum (Gramineae-Bambusoideae) from Borneo and Sumatra[J]. Kew Bulletin, 1938, 38: 321-332.
121. Airy Shaw HK. Notes on Malaysian and other Asiatic Euphorbiaceae[J]. Kew Bulletin, 1966, 20: 25-49+ii.
122. Airy Shaw HK. Notes on Malesian and other Asiatic Euphorbiaceae[J]. Kew Bulletin, 1969, 23: 1-131+iii.
123. Airy Shaw HK. Notes on Malesian and other Asiatic Euphorbiaceae[J]. Kew Bulletin, 1974, 29: 281-331.
124. Hind DJN. Novaguinea (Compositae: Astereae: Lagenophrinae), a new endemic genus to Papua, Indonesia. Contributions to the Flora of Mount Jaya, XIII[J]. Kew Bulletin, 2004, 59: 177-188.
125. Esser HJ. A partial revision of the Hippomaneae (Euphorbiaceae) in Malesia[J]. Blumea, 1999, 44: 149-215.
126. Adema F. Notes on Malesian Fabaceae (Leguminosae-Papilionoideae). 7. The genus Millettia[J]. Blumea, 2000, 45: 403-425.
127. Slik JWF, van Welzen PC. A taxonomic revision of Mallotus sections Hancea and Stylanthus (Euphorbiaceae)[J]. Blumea, 2001, 46: 3-66.
128. Hu CM. New species of Ardisia (Myrsinaceae) from Malesia[J]. Blumea, 2002, 47: 493-512.
129. Turner H, Veldkamp JF. Parnassia (Parnassiaceae) in North Sumatra[J]. Blumea, 2001, 46: 599-603.

130. van Steenis CGGJ. Pentastemona, a new 5-merous genus of Monocotyledons from North Sumatra (Stemonaceae)[J]. Blumea, 1982, 28: 151-163.
131. Middleton DJ. Revision of Alyxia (Apocynaceae). Part 1: Asia and Malesia[J]. Blumea, 2000, 45: 1-146.
132. Chambers TC, Farrant PA. Revision of Blechnum (Blechnaceae) in Malesia[J]. Blumea, 2001, 46: 283-350.
133. Zona S. Revision of Drymophloeus (Areceacea: Arecoideae)[J]. Blumea, 1999, 44: 1-24.
134. Veldkamp JF. Revision of Eragrostis (Gramineae, Chloridoideae) in Malesia[J]. Blumea, 2002, 47: 157-204.
135. Hendrian, Middleton D. Revision of Rauvolfia (Apocynaceae) in Malesia[J]. Blumea, 1999, 44: 449-470.
136. Stuppy W, van Welzen PC, Klinratana P, et al. Revision of the genera Aleurites, Reutealis and Vernicia (Euphorbiaceae)[J]. Blumea, 1999, 44: 73-98.
137. Bodegom S, Veldkamp JF. Revision of the pseudo-stipular species of Medinilla (Melastomataceae)[J]. Blumea, 2001, 46: 527-567.
138. Vink W. Some Malesian species of Pouteria (Sapotaceae)[J]. Blumea, 2001, 47: 95-147.
139. Slik JWF, Hovenkamp P, Iqbal M, et al. Structure, plant species diversity and plant species composition of the Gunung Lumut Protection Forest[M]. Leiden: National Herbarium Nederland, Leiden University Branch, 2007.
140. Liede S. The genera Cynanchum and Vincetoxicum (Apocynaceae-Asclepiadoideae) in Malesia[J]. Blumea, 1999, 44: 471-495.
141. Forster PI, van Welzen PC. The Malesian species of Choriceras, Fontainea, and Petalostigma (Euphorbiaceae)[J]. Blumea, 1999, 44: 99-107.
142. van Welzen PC, Tyas KN, Gaerlan E, et al. The Malesian species of Melanolepis (Euphorbiaceae)[J]. Blumea, 1999, 44: 437-446.
143. Slik JWF. Three new Malesian species of Mallotus section Hancea (Euphorbiaceae)[J]. Blumea, 1998, 43: 225-232.
144. Nurmawati S. Malesian Species of Dasymaschalon (Annonaceae)[J]. Floribunda, 2003, 2: 57-87.
145. Wiriadinata H, Girmansyah D. Tumbuhan Begonia (Begoniaceae) di Kecamatan Uluiwoi, Kabupaten Kolaka-Sulawesi Tenggara[J]. Floribunda, 2011, 2, 75-80.
146. Akhriadi P, Hernawati, Tamin R. A new species of Nepenthes (Nepenthaceae) from Sumatra[J]. Reinwardtia, 2004, 12: 141-144.

147. Adjie B, Kurniawan A, Sahashi N, et al. Dicksonia timorense (Diksoniaceae), a hemi-epiphytic new species of tree fern endemic on Timor Island, Indonesia[J]. Reinwardtia, 2012, 13: 357-362.
148. Kostermans AJGH. The genus Durio Adans. (Bombac.)[J]. Reinwardtia, 1958, 4: 357-460.
149. Sinclair J. Notes on New Guinea Annonaceae-Part I[J]. Gardens' Bulletin Singapore, 1956, 15: 4-13.
150. Ashton PS. Taxonomic Notes on Bornean Dipterocarpaceae, III[J]. Gardens' Bulletin Singapore, 1967, 22: 259-352.
151. Bremer K. A Check-list of the Memecylon Species (Melastomataceae) in Borneo, Java, Malaya and Sumatra[J]. Gardens' Bulletin Singapore, 1982, 35: 45-49.
152. Stone BC. Materials for a Monograph of Freycinetia Gaud. (Pandanaceae) VI. Species of Borneo[J]. Gardens' Bulletin Singapore, 1970, 25: 209-233.
153. Hay A. The genus Alocasia (Araceae-Colocasieae) in West Malesia and Sulawesi[J]. Gardens' Bulletin Singapore, 1998, 50: 221-334.
154. Boyce PC. The Genus Rhaphidophora Hassk. (Araceae-Monsteroideae-Monstereae) in New Guinea, Australia and the Tropical Western Pacific[J]. Gardens' Bulletin Singapore, 2001, 53: 75-183.
155. Chung RCK, Soepadmo E. A Synopsis of the Bornean Species of Microcos L.(Tiliaceae)[J]. Gardens' Bulletin Singapore, 2005, 57: 101-130.
156. Staples GW. A Checklist of Merremia (Convolvulaceae) in Australasia and the Pacific[J]. Gardens' Bulletin Singapore, 2010, 61: 483-522.
157. Arifiani D. Newly recorded Endiandra R. Br. (Lauraceae) from Waigeo Island, Raja Ampat, Papua, Indonesia[J]. Gardens' Bulletin Singapore, 2010, 62: 23-30.
158. Corner EJH. Check-list of Ficus in Asia and Australasia with keys to identification[J]. Gardens' Bulletin Singapore, 1965, 21: 1-186.
159. Purwaningsih. Diversitas Flora Di Kawasan Koridor Taman Nasional Gunung Halimun-Salak[J]. Edisi Khusus "Hari Lingkungan Hidup", 2012, 41-56.
160. Rohman F, Juma Y, Sulisetijono, et al. Plants diversity as a medicinal plants by the Tengger Tribe, Bromo Tengger Semeru National Park, East Java, Indonesia[J]. EurAsian Journal of BioSciences, 2019, 13: 2293-2298.
161. Garsetiasih R, Heriyanto NM. Karakteristik habitat banteng (Bos javanicus d'Alton 1832) di Taman Nasional Meru Betiri, Jawa Timur[J]. Jurnal Penelitian Hutan dan Konservasi Alam, 2014, 11: 77-89.
162. Syarif LI, Junita AR, Hatta M, et al. A mini review: medicinal plants for typhoid

fever in Indonesia[J]. Systematic Reviews in Pharmacy, 2020, 11: 1171-1180.
163. Batubara I, Prastya ME. Potential Use of Indonesian Medicinal Plants for Cosmetic and Oral Health: A Review[J]. Jurnal Kimia Valensi, 2020, 6: 118-132.
164. Merrill ED. New or Noteworthy Bornean Plants. (PART I.)[J]. Journal of the Straits Branch of the Royal Asiatic Society, 1922, 85: 151-201.
165. Coode MJE. Elaeocarpus for Flora Malesiana: the Coilopetalum group in Sulawesi & Maluku[J]. Kew Bulletin, 2001, 56: 837-874.
166. Coode MJE. Elaeocarpus in the Flora Malesiana area-E. kraengensis and ten new species from Sulawesi[J]. Kew Bulletin, 1995, 50: 267-294.
167. Hadiah JT. Establishment of Enrekang Botanic Garden, South Sulawesi: an effort to conserve plant diversity in the Wallacea region[J]. Gardens' Bulletin Singapore, 2011, 63: 465-470.
168. Nurainas. Artabotrys (Annonaceae) in Sumatra[J]. Floribunda, 2004, 2: 117-144.
169. Leksikowati SS, Oktaviani I, Ariyanti Y, et al. Medicial plant ethnobotany in local communities of Lampung tribe in West Lampung Regency[J]. Biologica Samudra, 2020, 2: 34-53.
170. Takano A, Okada H. Taxonomy of Globba (Zingiberaceae) in Sumatra, Indonesia[J]. Systematic Botany, 2003, 28: 524-546.
171. Fici S. A new species of Capparis L. (Capparaceae) from Sumatra (Indonesia)[J]. Adansonia, 2021, 43: 61-66.
172. Bean AR. A revision of Baeckea (Myrtaceae) in eastern Australia, Malesia and South-East Asia[J]. Telopea, 1997, 7: 245-268.
173. Boyce PC, Hay A. A taxonomic revision of Araceae tribe Potheae (Pothos, Pothoidium and Pedicellarum) for Malesia, Australia and the tropical Western Pacific[J]. Telopea, 2001, 9: 449-571.
174. Hay A, Yuzammi. Schismatoglottideae (Araceae) in Malesia I-Schismatoglottis[J]. Telopea, 2000, 9: 1-177.
175. Bogner J, Hay A. Schismatoglottideae (Araceae) in Malesia II-Aridarum, Bucephalandra, Phymatarum and Piptospatha[J]. Telopea, 2000, 9: 179-222.
176. Agustini V, Suharyanto S, Suharno S, et al. The Diversity of Tropical Orchids of South Papua[J]. Jurnal Biologi Papua, 2018, 5: 1-9.
177. Bakhuizen van den Brink R.C. A contribution to the knowledge of the Melastomataceae occurring in the Malay Archipelago especially in the Netherlands East Indies[J]. Recueil des travaux botaniques néerlandais, 1943, 40: 1-391.

178. Holthuis L, Lam HJ. A first contribution to our knowledge of the flora of the Talaud Islands and Morotai[J]. Blumea, 1942, 5: 93-256.
179. Grey-Wilson C. A revision of Sumatran Impatiens: Studies in Balsaminaceae: VIII[J]. Kew Bulletin, 1989, 44: 67-106.
180. Mustaqim WA, Putra HF, Fakhrurrozi Y, et al. A new record of Euphorbia atoto (Euphorbiaceae) in Bangka Belitung and notes of Coptosapelta hammii (Rubiaceae) for Borneo[J]. Journal of Tropical Biology and Conservation, 2019, 16: 151-159.
181. Koizumi M, Nagamasu H. A New Record of the Monotypic Harmandia (Olacaceae) from East Kalimantan, Indonesia[J]. Acta Phytotaxonomica et Geobotanica, 2005, 56: 257-259.
182. Okada H, Tsukaya H. A new species of Aridarum (Araceae: Schismatoglottideae) from West Kalimantan, Indonesian Borneo[J]. Acta Phytotaxonomica et Geobotanica, 2013, 63: 71-75.
183. Okada H, Tsukaya H. A new species of Piptospatha (Araceae: Schismatoglottideae) from West Kalimantan, Indonesian Borneo[J]. Acta Phytotaxonomica et Geobotanica, 2010, 61: 87-92.
184. Takano A, Nagamasu H. A new species, Etlingera palangkensis (Zingiberaceae) from Borneo[J]. Acta Phytotaxonomica et Geobotanica, 2006, 57: 75-79.
185. Tsukaya H, Okada H. A new variety of Didymoplexis cornuta (Orchidaceae) from West Kalimantan, Borneo[J]. Acta Phytotaxonomica et Geobotanica, 2012, 62: 89-93.
186. Kanis A. A review of the Amaranthaceae in Papuasia[J]. Contributions from Herbarium Australiense, 1972, 1: 3-18.
187. Baker WJ, Dransfield J. Calamus longipinna (Arecaceae: Calamoideae) and its relatives in New Guinea[J]. Kew Bulletin, 2002, 57: 853-866.
188. Nugroho GD, Aditya A, Dewi K. Keanekaragaman anggrek (Orchidaceae) di Taman Nasional Gunung Merbabu (TNGMb), Jawa Tengah[J]. Prosiding Seminar Nasional Masyarakat Biodiversitas Indonesia, 2018, 4: 195-201.
189. Sugau JB. Enumeration and Notes on Adinandra (Pentaphylacaceae) in Borneo[J]. Sandakania, 2008, 17: 5-41.
190. Chew W. A revision of the genus Poikilospermum (Urticaceae)[J]. Gardens' Bulletin Singapore, 1963, 20: 1-103.
191. Pereira JT. Four new species of Ridsdalea (Rubiaceae: Gardenieae) from Borneo, Wallacea and New Guinea[J]. Sandakania, 2016, 22: 77-95.

192. Utami N. Impatiens talakmauensis (Balsaminaceae), a New Species from Western Sumatra, Indonesia[J]. Acta Phytotaxonomica et Geobotanica, 2012, 63: 51-54.
193. Häkkinen M, Meekiong K. Musa borneensis Becc. (Musaceae) and its intraspecific taxa in Borneo[J]. Acta Phytotaxonomica et Geobotanica, 2005, 56: 213-230.
194. Schuiteman A, Wanma JF. New and Noteworthy Orchid Species from the Arfak Mountains, West Papua Province, Indonesia[J]. Malesian Orchid Journal, 2017, 20: 75-90.
195. Zahid MS. Nine novelties in Porterandia (Rubiaceae) from Borneo and Sulawesi[J]. Sandakania, 2004, 15: 55-78.
196. Furtado CX. Notes on Some Malaysian Melastomaceae[J]. Gardens’ Bulletin Singapore, 1963, 20: 105-122.
197. Hidayat A. The Fern diversity of South East Sulawesi[D]. Bogor: Bogor Agricultural University, 2011.
198. Lasut MT. The floristic study of herbaceous grasses in Sulawesi[D]. Bogor: Bogor Agricultural University, 2009.
199. Akbarini D. The species of Polyalthia in Berau regency, East Kalimantan[D]. Bogor: Bogor Agricultural University, 2002.
200. Macklin J. A systematic revision of the Santalaceae R.Br. of Southeast Asia[D]. Ireland: University of Dublin, 2000.
201. Lubis SR. Keanekaragaman dan Pola distribusi tumbuhan Paku di Hutan Wisata Alam Taman Eden Kabupaten Toba Samosir Provinsi Sumatera Utara[D]. Medan: Universitas Sumatera Utara, 2009.
202. Saputri, A. Biodiversitas Bambu di Sumatera Utara Bagian Timur[D]. Medan: Universitas Sumatera Utara, 2013.
203. Alsahana I. Inventarisasi Jenis-Jenis Arecaceae Di Stasiun Penelitian Soraya Kawasan Ekosistem Leuser Kecamatan Sultan Daulat Kota Subulussalam[D]. Medan: Universitas Sumatera Utara, 2021.
204. Lubis A. Keanekaragaman Piperaceae dan Rubiaceae di Taman Wisata Alam Deleng Lancuk Kabupaten Karo Sumatera Utara[D]. Medan: Universitas Sumatera Utara, 2008.
205. Hutasuhut MA. Studi Tumbuhan Herba di Hutan Sibayak I[D]. Medan: Universitas Sumatera Utara, 2011.
206. Sinaga NI, Keim AP, Puradyatmika P. The unique characters and habitat of Freycinetia (Pandanaceae) with seven new species in Timika, West Papua,

Indonesia[J]. Reinwardtia, 2013, 13: 405-418.
207. Vermeulen JJ, O’byrne P. Thirty Two New Species of Bulbophyllum (Orchidaceae) from Sulawesi[J]. Gardens’ Bulletin Singapore, 2008, 60: 73-153.
208. Okada H. Three New Species of Annonaceae from West Kalimantan, Indonesian Borneo[J]. Acta Phytotaxonomica et Geobotanica, 2014, 65: 17-24.
209. HR EA, Tambaru E, Salam HMA, et al. Jenis Jenis Tumbuhan Berpotensi Obat Di Desa Bambapuang Kabupaten Enrekang[J]. Jurnal Ilmu Alam dan Lingkungan, 2018, 9: 1-7.
210. Williams LO. Orchidaceae novae guineae I[J]. Botanical Museum leaflets, Harvard University, 1946, 12: 149-177.
211. Kloss CB. Spolia Mentawiensia[J]. Bulletin of miscellaneous information, 1926, 2: 56-94.
212. Damayanto IPGP, Widjaja EA. A noteworthy Dendrocalamus (Poaceae: Bambusoideae) from Sumatra, Indonesia[J]. Gardens’ Bulletin Singapore, 2017, 69: 75-80.
213. Juhonewe NS, Rodda M. Contribution to a revision of Hoya (Apocynaceae: Asclepiadoideae) of Papuasia. Part I: ten new species, one new subspecies and one new combination[J]. Gardens’ Bulletin Singapore, 2017, 69: 97-147.
214. Ezedin Z, Weiblen GD. Additions and changes to Ficus (Moraceae) in New Guinea with comments on the world’s largest fig[J]. Gardens’ Bulletin Singapore, 2019, 71: 197-216.
215. Setiaji A, Muna A, Jati FP, et al. Keanekaragaman anggrek di Daerah Istimewa Yogyakarta[J]. Prosiding Seminar Nasional Masyarakat Biodiversitas Indonesia, 2018, 4: 63-68.
216. Batoro J, Indriyani S, Yanuwiyadi B. Survey and Utilization of Wild Plants (Extractivism) in Tengger Society East Java, Indonesia[J]. International Journal of Modern Botany, 2018, 8: 8-14.
217. Silalahi M, Nisyawati, Anggraeni R. Studi Etnobotani Tumbuhan Pangan yang Tidak Dibudidayakan oleh Masyarakat Lokal Sub-etnis Batak Toba, di Desa Peadungdung Sumatera Utara, Indonesia[J]. Jurnal Pengelolaan Sumberdaya Alam dan Lingkungan, 2018, 8: 241-250.
218. Navia ZI, Suwardi AB, Harmawan T, et al. The diversity and contribution of indigenous edible fruit plants to the rural community in the Gayo Highlands, Indonesia[J]. Journal of Agriculture and Rural Development in the Tropics and Subtropics, 2020, 121: 89-98.

219. Usmadi D, Witono JR, Siregar M, et al. Keanekaragaman dan status konservasi tumbuhan di hutan in situ Kebun Raya Tanjung Puri Tabalong, Kalimantan Selatan[J]. Prosiding Seminar Nasional Masyarakat Biodiversitas Indonesia, 2018, 4: 304-309.
220. Amianti IP, Mustika SJ, Adriyanti DT, et al. Komposisi jenis tumbuhan dan struktur hutan kota di Kabupaten Trenggalek, Jawa Timur[J]. Prosiding Seminar Nasional Masyarakat Biodiversitas Indonesia, 2019, 5: 139-144.
221. Jasni TK. Prioritas penelitian dan pengembangan jenis rotan andalan setempat[J]. Prosiding Seminar Nasional Masyarakat Biodiversitas Indonesia, 2015, 1: 1868-1876.
222. Okada H, Tsukaya H, Soejima A. A new species of Heteroblemma (Melastomataceae) from West Kalimantan, Borneo, Indonesia[J]. Acta Phytotaxonomica et Geobotanica, 2017, 68: 101-104.
223. Wang WG, Randi A, Wang CXL, et al. Begonia daunhitam, a new species of Begonia (Begoniaceae) from West Kalimantan, Indonesia[J]. Taiwania, 2020, 65: 27-32.
224. Lin CW, Peng CI. Begonia natunaensis (sect. Reichenheimia, Begoniaceae), a new species from Natuna Island, Indonesia[J]. Taiwania, 2014, 59: 368-373.
225. Ardi WH, Girmansyah D, Zulfadii, et al. Begonia willemii, a new species of Begonia from Sulawesi, Indonesia[J]. Taiwania, 2021, 66: 374-377.
226. Setiawan AW, Asdini S, Chikmawati T, et al. Claoxylon (sect. Affinia) dipulvinum (Euphorbiaceae), a new species from the Moluccas, Indonesia[J]. Taiwania, 2020, 65: 348-352.
227. Suetsugu K, Hidayat A, Tsukaya H. First Record of the Mycoheterotrophic Plant Gastrodia spathulata (Orchidaceae) from West Java, Indonesia[J]. Acta Phytotaxonomica et Geobotanica, 2018, 69: 135-137.
228. Wongso S, Asih NPS, Bastmeijer JD, et al. Four new Cryptocoryne (Araceae) from Sumatera, Indonesia: a new variety and three interspecific natural hybrids[J]. Taiwania, 2019: 64, 326-338.
229. Nurmawati S, Ariyanti NS, Chikmawati T. Monoon longipetalum (Annonaceae) - a new species from Sumatra, Indonesia[J]. Taiwania, 2019, 64: 235-239.
230. Suetsugu K, Metusala D, Yudistira YR. New Distributional Record of Lecanorchis nigricans Honda (Orchidaceae) and a New Addition for the Orchid Flora of Indonesia[J]. Acta Phytotaxonomica et Geobotanica, 2021, 72: 67-72.
231. Ormerod P. Studies of West Malesian Agrostophyllum Blume (Orchidaceae) 2[J].

Taiwania, 2014, 59: 331-339.
232. Mendum M, Atkins HJ. The Gesneriaceae of Sulawesi I: An introduction[J]. Edinburgh Journal of Botany, 2004, 60: 299-304.
233. Naive MAK, Yudistira YR, Romiyadi, et al. Tuberolabium camperenik (Orchidaceae), a new Aeridinae species with crystal white flowers from West Java, Indonesia[J]. Taiwania, 2021, 66: 273-276.
234. Koizumi M, Nagamasu H. Two New Species and Notes on Bornean Praravinia (Rubiaceae)[J]. Acta Phytotaxonomica et Geobotanica, 2016, 67: 175-184.
235. Grosvenor PW, Gothard PK, McWilliam NC, et al. Medicinal plants from Riau Province, Sumatra, Indonesia. Part 1: Uses[J]. Journal of Ethnopharmacology, 1995, 45: 75-95.
236. Grosvenor PW, Supriono A, Gray DO. Medicinal plants from Riau Province, Sumatra, Indonesia. Part 2: antibacterial and antifungal activity[J]. Journal of Ethnopharmacology, 1995, 45: 97-111.
237. Elliott S, Brimacombe J. The medicinal plants of Gunung Leuser National Park, Indonesia[J]. Journal of Ethnopharmacology, 1987, 19: 285-317.
238. Mahyar UW, Burley JS, Gyllenhaal C, et al. Medicinal plants of Seberida (Riau Province, Sumatra, Indonesia)[J]. Journal of Ethnopharmacology, 1991, 31: 217-237.
239. Phillips PD, Yasman I, Brash TE, et al. Grouping tree species for analysis of forest data in Kalimantan (Indonesian Borneo)[J]. Forest Ecology and Management, 2002, 157: 205-216.
240. Sleumer H. Florae Malesianae Praecursores XIV* A revision of the genus Diplycosia (Ericaceae)[J]. Reinwardtia, 1957, 4: 119-161.
241. Sleumer H. Florae Malesianae Praecursores XV The genus Gaultheria in Malaysia[J]. Reinwardtia, 1957, 4: 163-188.
242. van Slooten DF. Sertulum Dipterocarpacearum Malayensium-V*[J]. Reinwardtia, 1952, 2: 1-68.
243. de Wit HCD. A revision of the genus Archidendron F. Muell. (Mimosaceae)[J]. Reinwardtia, 1952, 2: 69-96.
244. Kern JH. Notes on Malaysian Cyperaceae[J]. Reinwardtia, 1952, 2: 97-130.
245. Bloembergen S. A critical study in the complex-polymorphous genus Schima (Theaceae)[J]. Reinwardtia, 1952, 2: 133-183.
246. Jansen P. Notes on Malaysian grasses-I*[J]. Reinwardtia, 1953, 2: 225-350.
247. Kostermans AJGH. New and critical Malaysian plants-I[J]. Reinwardtia, 1953, 2:

357-366.

248. Praptosuwiryo TN. The Rare Pteridophytes of Mt. Slamet with Three Species New Records for Java[J]. Floribunda, 2013, 4: 138-146.

249. Nelmes E. The genus Carex in Malaysia[J]. Reinwardtia, 1951, 1: 221-450.

250. Kostermans AJGH. The genus Teijsmanniodendron Koorders (Verbenaceae)[J]. Reinwardtia, 1951, 1: 75-106.

251. Helmi N, Kartawinata K, Samsosein I. An undescribed lowland natural forest at Bodogol, Gunung Gede Pangrango National Park, Cibodas Biosphere Reserve, West Java, Indonesia[J]. Reinwardtia, 2009, 13: 33-46.

252. Kartonegoro A. Ericaceae of Latimojong Range, South Sulawesi[J]. Floribunda, 2014, 4: 191-194.

253. Sambas EN, Takeuchi A, Maturbongs RA. Komunikasi Pendek Koleksi Tumbuhan Dari Semenanjung Bomberai[J]. Berita Biologi, 2003, 6: 719-726.

254. Pasaribu N, Widjaja EA. Notes on Freycinetia (Pandanaceae) from Jambi, Sumatra with the description of a new species[J]. Reinwardtia, 2009, 13: 87-92.

255. Liana A, Purnomo, Sumardi I, et al. Bamboo Species (Poaceae: Bambusoideae) from Selayar Island[J]. Floribunda, 2017, 5: 185-191.

256. Kartawinata K, Apandi A. Checklist of plant species on the Peucang island (Ujung Kulon Nature Reserve, West Java)[J]. Berita Biologi, 1977, 2: 13-18.

257. Dalimunthe SH, Chikmawati T, Widjaja EA. Revisi Ampelocissus (Vitaceae) di Sumatra[J]. Floribunda, 2016, 5: 165-174.

258. Uji T. Keanekaragaman Dan Potensi Flora Di Cagar Alam Pegunungan Cyclops, Papua[J]. Jurnal Teknologi Lingkungan, 2005, 6: 485-495.

259. Fujii S, Nishimura S, Yoneda T. Altitudinal distribution of Fagaceae in West Sumatra[J]. Tropics, 2006, 15: 153-163.

260. Kiew R. Chionanthus (Oleaceae) in Sulawesi, Indonesia, including three new species[J]. Reinwardtia, 2015, 14: 287-295.

261. Wong KM, Razafimandimbison SG. A new combination and a new name in Gynochthodes (Rubiaceae)[J]. Reinwardtia, 2015, 14: 297-298.

262. Ardiyani M. A new species of Zingiber (Zingiberaceae) from Enggano Island, Indonesia[J]. Reinwardtia, 2015, 14: 307-310.

263. Ardi WH, Ardiyani M. Two new species of Alpinia (Zingiberaceae) from Sulawesi, Indonesia[J]. Reinwardtia, 2015, 14: 311-316.

264. Mahyuni R, Kusuma YWC, Wihermanto, et al. Notes on Rafflesia (Rafflesiaceae) in Sumatra with a new record Rafflesia gadutensis Meijer[J]. Reinwardtia, 2015,

14: 317-322.
265. de Wilde WJJO, Duyfjes BEE, Rugayah. Gymnopetalum pectinatum (W. J. De Wilde & Duyfjes) Rugayah: rank of species for Gymnopetalum scabrum var. pectinatum (Cucurbitaceae)[J]. Reinwardtia, 2015, 14: 323-324.
266. Hartini S. Keanekaragaman Jenis Tumbuhan Paku (Pteridophyta) Di Kawasan Hutan Tumbang Manggu, Kecamatan Sanaman Mantikei, Kabupaten Katingan, Kalimantan Tengah[J]. Ekologia, 2020, 20: 1-13.
267. Mahyuni R, Chikmawati T, Ariyanti NS, et al. The Psydrax dicoccos Complex (Rubiaceae) in Malesia, with Three New Species[J]. Floribunda, 2018, 5: 322-331.
268. Susiarti S, Rahayu M, Royyani MF, et al. Pengetahuan dan Pemanfaatan Tumbuhan Obat Masyarakat Tobelo dalam di Maluku Utara[J]. Media Penelitian dan Pengembangan Kesehatan, 2015, 25: 211-218.
269. Soepadmo. A monograph op the genus Neesia* Blume (Bombacaceae)[J]. Reinwardtia, 1960, 5: 481-508.
270. Wiriadinata H, Sari R. A new species of Rafflesia (Rafflesiaceae) from North Sumatra[J]. Reinwardtia, 2010, 13: 95-100.
271. Kartonegoro A, Veldkamp JF. A revision of Dissochaeta (Melastomataceae) in Java[J]. Reinwardtia, 2010, 13: 125-145.
272. Pasaribu N. Two new species of Freycinetia (Pandanaceae) from Sumatra, Indonesia[J]. Reinwardtia, 2010, 13: 147-150.
273. Keim AP, Rahayu M. Pandanaceae of Sumbawa, West Nusa Tenggara, Indonesia[J]. Reinwardtia, 2010, 13: 151-158.
274. Mat-Salleh K, Mahyuni R, Susatya A, Veldkamp JF. Rafflesia lawangensis (Rafflesiaceae), a new species from Bukit Lawang, Gunung Leuser National Park, North Sumatra, Indonesia[J]. Reinwardtia, 2010, 13: 159-165.
275. Sinaga NI. Two new species of Freycinetia (Pandanaceae) from Manokwari, West Papua[J]. Reinwardtia, 2010, 13: 183-187.
276. Selviana, Nurtjahya E, Sulistiarini D. Jenis-jenis Anggrek (Orchidaceae) di Hutan Rawa Gambut Kabupaten Belitung[J]. Floribunda, 2019, 6: 72-80.
277. Sinaga NI, Megia R, Hartana A, et al. The ecology and distribution of Freycinetia Gaud. (Pandanaceae; Freycinetoidea) in Indonesian New Guinea[J]. Reinwardtia, 2010, 13: 189-197.
278. Utami N, Wiriadinata H. Impatiens mamasensis (Balsaminaceae), a new species from West Celebes, Indonesia[J]. Reinwardtia, 2010, 13: 211-212.

279. Ardiyani M, Poulsen AD, Suksathan P, et al. Marantaceae in Sulawesi[J]. Reinwardtia, 2010, 13: 213-220.
280. Irsyam ASD, Chikmawati T. Ikhtisar Suku Rutaceae di Madura[J]. Floribunda, 2018, 5: 277-290.
281. Gunawan, Chikmawati T, Sobir, et al. Distribution, Morphological Variation and New Variety of Baccaurea angulata Merr. (Phyllanthaceae)[J]. Floribunda, 2018, 6: 1-11.
282. Rijaya I, Fitmawati. Jenis-jenis Bambu (Bambusoideae) di Pulau Bengkalis, Provinsi Riau, Indonesia[J]. Floribunda, 2019, 6: 41-52.
283. Syam N, Chikmawati T, Rustiami H. A phenetic study of the Calamus flabellatus complex (Palmae) in West Malesia[J]. Reinwardtia, 2016, 15: 27-41.
284. Ardhaka IM, Ardi WH, Undaharta NKE, et al. A new species Begonia from Manusela National Park, Seram[J]. Reinwardtia, 2016, 15: 61-64.
285. Apal RU, Ariyanti NS, Walujo EB, et al. Pemanfaatan Tumbuhan Obat oleh Suku Togutil di Daerah Penyangga Taman Nasional Aketajawe Lolobata[J]. Jurnal Sumberdaya Hayati, 2018, 4: 21-27.
286. Mambrasar YM. Rhododendron gumineense Craven (Ericaceae, Subgenus Vireya), a new record for Indonesia[J]. Floribunda, 2018, 6: 19-21.
287. Hartley TG. Two new species of Acronychia (Rutaceae) from New Guinea[J]. Reinwardtia, 1982, 10: 93-96.
288. Rugayah, Sunarti S. Two new wild species of Averrhoa (Oxalidaceae) from Indonesia[J]. Reinwardtia, 2008, 12: 325-331.
289. Turnbull JR, Middleton AT. Three new Nepenthes from Sulawesi Tengah[J]. Reinwardtia, 1984, 10: 107-111.
290. Wibowo T, Utama P, Amzu E. Potensi dan Upaya Pelestarian Pemanfaatan Tumbuhan Obat di Taman Nasional Meru Betiri[J]. Media Konservasi, 1991, 3: 28-42.
291. Putri NH, Raksun A, Mertha IG. Identifikasi Tumbuhan Paku Sejati (Filicopytha) di Kawasan Hutan Wisata Aik Nyet sebagai Sumber Belajar Biologi[J]. Jurnal Biologi Tropis, 2018, 18: 104-108.
292. Widjaja EA. A revision of Malesian Gigantochloa (Poaceae-Bambusoideae)[J]. Reinwardtia, 1987, 10: 291-380.
293. Sulistiarini D. The orchid genus Luisia in Indonesia[J]. Reinwardtia, 1988, 10: 383-398.
294. Widjaja EA. Three new species of Dinochloa (Poaceae, Bambusoideae) with

erect culm sheath blades from Sulawesi, Indonesia[J]. Reinwardtia, 2009, 12: 435-440.

295. Maruzy A, Mujahid R. Status Konservasi Tumbuhan obat Provinsi Papua dan Papua Barat (Indonesia)[J]. Media Konservasi, 2019, 24: 114-123.

296. Izzuddin MQ, Azrianingsih R. Inventarisasi Tumbuhan Obat di Kampung Adat Urug, Desa Urug, Kecamatan Sukajaya, Kabupaten Bogor[J]. Natural B, 2015, 3: 81-92.

297. Damayanto IPGP, Arinasa IBK, Tirta IG, et al. A New Record of Chloothamnus Buse (Poaceae: Bambusoideae) from Sumbawa Island and Notes on the Genus in Malesia[J]. Floribunda, 2020, 6: 127-132.

298. Pritchett R, Phillips A, Mardiastuti A, et al. Rattan diversity and broad edaphic niches in a tropical rainforest of Buton, Sulawesi, Indonesia[J]. Reinwardtia, 2016, 15: 99-110.

299. Astuti IP, Rugayah. A new species of Murraya from Cyclops Mountain Papua[J]. Reinwardtia, 2016, 15: 111-114.

300. Girmansyah D. A new species of Begonia (Begoniaceae) from Sumbawa, Lesser Sunda Islands, Indonesia[J]. Reinwardtia, 2016, 15: 115-118.

301. Damayanto IPGP, Widjaja EA. A new species of Schizostachyum (Poaceae: Bambusoideae) from Sumba Island, Indonesia[J]. Reinwardtia, 2016, 15: 119-122.

302. Veldkamp JF. A revision of Iseilema (Gramineae) in Malesia[J]. Reinwardtia, 2016, 15: 123-127.

303. Sa'adah VS, Zuhud EAM, Siswoyo. Potensi Pemanfaatan Tumbuhan Aromatik di Resort Kembang Kuning, Taman Nasional Gunung Rinjani, Nusa Tenggara Barat[J]. Media Konservasi, 2019, 24: 1-10.

304. Irsyam ASD, Chikmawati T. Peninjauan Ulang Marga Citrus (Rutaceae) di Kawasan Madura[J]. Floribunda, 2015, 5: 82-91.

305. Wibowo ARU, Juswara LS. A new species of Appendicula section Pododesme (Orchidaceae) from Indonesia[J]. Reinwardtia, 2017, 16: 65-71.

306. Mustaqim WA, Nisyawati. Wild native plants in urban ecosystems around Jakarta: a checklist of the woody species of Universitas Indonesia[J]. Jurnal Pro-Life, 2020, 7: 49-60.

307. Rustiami H, Henderson A. A synopsis of Calamus (Arecaceae) in Sulawesi[J]. Reinwardtia, 2017, 16: 49-63.

308. Ervianti D, Widjaja EA, Sedayu A. Bamboo diversity of Sulawesi, Indonesia[J].

Biodiversitas, 2019, 20: 91-109.
309. Huang YL, Fritsch PW, Shi SH. A Revision of the Imbricate Group of Styrax Series Cyrta (Styracaceae) in Asia[J]. Annals of the Missouri Botanical Garden, 2003, 90: 491-553.
310. Rugayah. Annonaceae dari Wawonii, Sulawesi Tenggara[J]. Jurnal Biologi Indonesia, 2014, 10: 67-76.
311. Mambrasar YM, Schuiteman A. Trichotosia Gabriel-Asemiana (Orchidaceae), A new species from Tambrauw, West Papua, Indonesia[J]. Reinwardtia, 2017, 16: 107-110.
312. Rugayah, Sunarti S. The genus Lasianthus (Rubiaceae) in Wawonii Island, Southeast Sulawesi, Indonesia[J]. Reinwardtia, 2017, 16: 97-101.
313. Hashimoto Y, Yusro F, Mariani Y, et al. Ethnopharmacological Study on Traditional Knowledge of Medicinal Plant Used from Secondary Forest in Community at Sekabuk Village, Mempawah District, West Kalimantan, Indonesia[J]. Wood Research Journal, 2020, 10: 61-70.
314. Damayanto IPGP. Dinochloa malayana S.Dransf. (Poaceae: Bambusoideae), a new record for Indonesia[J]. Reinwardtia, 2018, 17: 35-37.
315. Sunarti S. Syzygium tinombalum (Myrtaceae), a new species from Central Sulawesi, Indonesia[J]. Reinwardtia, 2020, 19: 87-91.
316. Sulistiarini D, Potter D, O'byrne P. Dendrobium tinukariensis, a new species of section Calyptrochilus from the Mekongga Mountains, South East Sulawesi, Indonesia[J]. Reinwardtia, 2017, 16: 103-106.
317. Mansur M, Kartawinata K. Phytosociology of a lower montane forest on Mt. Batulanteh, Sumbawa, Indonesia[J]. Reinwardtia, 2017, 16: 77-92.
318. Veldkamp JF. Poa opinata (Gramineae), a new species from G. Binaiya, Ceram, Moluccas, Indonesia[J]. Reinwardtia, 2017, 16: 73-75.
319. Rustiami H. Palms diversity of Gunung Kerinci and Gunung Tujuh, Kerinci Seblat National Park, Sumatra[J]. Floribunda, 2002, 2: 6-8.
320. Purwaningsih, Atikah TD. Diversitas Floristik dan Struktur Vegetasi di Hutan Gunung Payung, Taman Nasional Ujung Kulon[J]. Berita Biologi, 2018, 17: 335-349.
321. Veldkamp JF. A revision of Isachne in Malesia 2: Sect. Albentes (Gramineae, Isachneae)[J]. Reinwardtia, 2018, 17: 1-33.
322. Priyanti. Jenis-Jenis Annonaceae Pemanjat di Kabupaten Berau, Kalimantan Timur[J]. Floribunda, 2016, 2: 9-15.

323. Sambas EN, Kusmana C, Prasetyo LB, et al. Vegetation analysis and population structure of plants at Mount Endut forested area, Gunung Halimun Salak National Park, Banten, Java, Indonesia[J]. Reinwardtia, 2018, 17: 39-53.
324. Suratman. The Indonesian Species of Rennellia Korth. (Rubiaceae)[J]. Biodiversitas, 2008, 9: 259-263.
325. Sadili A, Kartawinata K, Soedjito H, et al. Tree species diversity in a pristine montane forest previously untouched by human activities in Foja Mountains, Papua, Indonesia[J]. Reinwardtia, 2018, 17: 133-154.
326. Wong KM, Mahyuni R. Flora of Singapore Precursors, 2. A new species and two new combinations in Psydrax (Rubiaceae: Vanguerieae) for West Malesia[J]. Reinwardtia, 2018, 17: 77-84.
327. Rahayu S, Astuti IP. Hoya decipulae (Apocynaceae, Asclepiadoideae), a new species from Sumatra[J]. Reinwardtia, 2019, 18: 43-50.
328. Mambrasar YM, Hutabarat PWK. Rhododendron meagaii, a new species of Rhododendron subgenus Vireya (Ericaceae) from Papua, Indonesia[J]. Reinwardtia, 2018, 17: 97-100.
329. Efendi M. The Stomata Type of Thirty Two Indonesian Native Vegonia of Cibodas Botanical Garden Collection[J]. Berita Biologi, 2019, 18: 175-182.
330. Heriyanto NM, Samsoedin I, Kartawinata K. Tree species diversity, structural characteristics and carbon stock in a one-hectare plot of the protection forest area in West Lampung Regency, Indonesia[J]. Reinwardtia, 2018, 18: 1-18.
331. Hartini S. Keanekaragaman Flora di Pulau Samosir, Sumatera Utara[J]. Berk. Penel. Hayati Edisi Khusus, 2009, 3A: 7-16.
332. Ardi WH, Thomas DC. A new species of Begonia (Begoniaceae) from South Sulawesi, Indonesia, and an augmented description of Begonia bonthainensis[J]. Reinwardtia, 2018, 18: 19-26.
333. Hendra M. Notes on a new record and a new species of Pinanga (Arecaceae) from Sumatra[J]. Floribunda, 2002, 2: 29-32.
334. Argent G, Mambrasar YM. Rhododendron widjajae (Ericaceae, section Schistanthe) a new species from Sulawesi[J]. Reinwardtia, 2019, 18: 27-30.
335. Erlinawati I, Asih NPS, Kurniawan A, et al. Studies on the Araceae of the Lesser Sunda Islands II: New record for Scindapsus hederaceus Miq. in Bali[J]. Reinwardtia, 2019, 18: 51-64.
336. Pratama BA, Mirmanto E. Analisis vegetasi di Palau Bintan, Kepulauan Riau[J]. Berita Biologi, 2019, 18: 315-324.

337. Ardiyani M, Poulsen AD. An update of the genus Etlingera (Zingiberaceae) in Sulawesi including the description of a new species[J]. Reinwardtia, 2019, 18: 31-42.
338. Ervianti D, Widjaja EA, Sedayu A. New species of climbing and scrambling bamboo from Sulawesi, Indonesia[J]. Reinwardtia, 2019, 18: 115-132.
339. Bouman RW, Keßler PJA, van Welzen PC. Lectotypification and amended description of Phyllanthus (Phyllanthaceae) species described by Koorders from Sulawesi, Indonesia[J]. Reinwardtia, 2019, 18: 97-103.
340. Firdausy B, Pujiastuti, Murdiyah S. Diversity of Shrub Plants in Kapas Biru Waterfall Lumajang District of East Java[J]. Bioedukasi, 2018, XVI: 47-60.
341. Kiew R. Towards a Flora of New Guinea: Oleaceae. Part 1. Jasminum, Ligustrum, Myxopyrum and Olea[J]. Reinwardtia, 2020, 19: 1-25.
342. Kalima T, Suharti S, Sumarhani. Tree species diversity and ethnobotany of degraded peat swamp forest in Central Kalimantan[J]. Reinwardtia, 2020, 19: 27-54.
343. Hambali GG, Sulistiarini D, Rugayah. Dracaena jiewhoei (Asparagaceae), a new endemic species from Sumatra, Indonesia[J]. Reinwardtia, 2020, 19: 75-79.
344. Widjaja EA. Notes on Fimbribambusa Widjaja, with a new species from the Lesser Sunda Islands[J]. Reinwardtia, 2020, 19: 55-59.
345. Ardi WH, Thomas DC. Begonia tjiasmantoi, a new species from West Sulawesi[J]. Reinwardtia, 2020, 19: 61-65.
346. Victoriano M, Yudistira YR. Bulbophyllum trinervosum, a new species of section Macrocaulia (Orchidaceae: Bulbophyllinae) from West Java, Indonesia[J]. Reinwardtia, 2020, 19: 67-73.
347. Kartonegoro A, Potter D. The Gesneriaceae of Sulawesi VI: The species from Mekongga Mts. with a new species of Cyrtandra described[J]. Reinwardtia, 2014, 14: 1-11.
348. Ardi WH, Kusuma YWC, Lewis CE, et al. Studies on Begonia (Begoniaceae) of the Molucca Islands I: two new species from Halmahera, Indonesia and an updated description of Begonia holosericea[J]. Reinwardtia, 2014, 14: 19-26.
349. Wawangningrum H, Puspitaningtyas DM. Keanekaragaman Araliaceae di Suaka Alam Sulasih Talang, Sumatera Barat dan Aklimatisasinya[J]. Biodiversitas, 2008, 9: 123-127.
350. Sukardjo S, Pratiwi R. Plant Diversity in Pari Island Marine Research Station of Indonesia South China Sea: Rare Plants and Their Conservation[J]. Malaysian

Journal of Science, 2016, 35: 192-212.

351. Ardiyani M, Ardi WH, Santoso W, et al. Etlingera tjiasmantoi (Zingiberaceae), a new species from Central Sulawesi[J]. Reinwardtia, 2020, 19: 103-108.
352. Gay H, Hennipman E, Huxley CR, et al. The Taxonomy, Distribution and Ecology of the Epiphytic Malesian Ant-Fern Lecanopteris Reinw. (Polypodiaceae)[J]. Gardens' Bulletin Singapore, 1993, 45: 293-335.
353. Vermeulen JJ, O'byrne P. Six New Species of Bulbophyllum (Orchidaceae) from Sulawesi[J]. Gardens' Bulletin Singapore, 2003, 55: 257-270.
354. de Wilde WJJO, Duyfjes BEE. New taxa and taxonomic status in Xanthophyllum Roxb. (Polygalaceae) from Borneo[J]. Gardens' Bulletin Singapore, 2005, 57: 47-61.
355. NG FSP. Taxonomic Notes on Bornean Cryptocarya R.Br. (Lauraceae)[J]. Gardens' Bulletin Singapore, 2005, 57: 63-68.
356. Girmansyah D, Wiriadinata H, Thomas DC, et al. Two new species and one new subspecies of Begonia (Begoniaceae) from Southeast Sulawesi, Sulawesi, Indonesia[J]. Reinwardtia, 2009, 13: 69-74.
357. Ariyanti EE, Pa'i. Inventarisasi Anggrek di Kabupaten Sintang, Kalimantan Barat[J]. Biodiversitas, 2008, 9: 21-24.
358. Prawiroatmodjo S, Kartawinata K. Floristic diversity and structural characteristics of mangrove forest of Raja Ampat, West Papua, Indonesia[J]. Reinwardtia, 2014, 14: 171-180.
359. Julia S, Kiew R. Diversity of Begonia (Begoniaceae) in Borneo-how many species are there?[J]. Reinwardtia, 2014, 14: 233-236.
360. Wiriadinata H, Girmansyah D, Hunter JM, et al. Floristic Study of West Sumbawa, Indonesia[J]. Reinwardtia, 2013, 13: 391-404.
361. Kartonegoro A. A revision of Rhynchoglossum (Gesneriaceae) in Malesia[J]. Reinwardtia, 2013, 13: 421-432.
362. Keim AP. A new species of Freycinetia Gaudich. (Pandanaceae; Freycinetoideae) from Tidore Island, Moluccas, Indonesia[J]. Reinwardtia, 2013, 13: 441-444.
363. Wiriadinata H. A new species of Begonia (Begoniaceae) from South Sulawesi, Indonesia[J]. Reinwardtia, 2013, 13: 445-448.
364. Rustiami H. Two new species of Daemonorops from Sulawesi[J]. Reinwardtia, 2009, 13: 25-30.
365. Hoover WS, Girmansyah D, Wiriadinata H, et al. Exploration of high elevation liana colonies on Mt. Slamet, Central Java, Indonesia[J]. Reinwardtia, 2009, 13:

45-67.
366. Keim AP. Three new species of Freycinetia (Pandanaceae) from Kalimantan, Indonesia[J]. Reinwardtia, 2009, 13: 15-20.
367. Rahayu S. Hoya (Apocynaceae: Asclepiadoideae) diversity in Gunung Gede Pangrango National Park, West Java, Indonesia[J]. Reinwardtia, 2012, 13: 331-339.
368. Sulistiarini D. The orchids genus Dilochia in Indonesia[J]. Reinwardtia, 2012, 13: 379-387.
369. de Wilde WJJO, Duyfjes BEE. Trichosanthes (Cucurbitaceae) in Malesia: additions and corrections, including a new species and a new variety[J]. Reinwardtia, 2012, 13: 221-228.
370. Sumadijaya A, Veldkamp JF. Non Bambusoid grasses (Gramineae) from Raja Ampat Archipelago, Papua Barat Province, Indonesia[J]. Reinwardtia, 2012, 13: 241-253.
371. Puspitaningtyas DM. Inventarisasi Anggrek dan Inangnya di Taman Nasional Meru Betiri - Jawa Timur[J]. Biodiversitas, 2007, 8: 210-214.
372. Keim AP. New variety, records & discoveries of some species of Pandanus (Pandanaceae) in Sumatra & Kalimantan, Indonesia[J]. Reinwardtia, 2012, 13: 255-262.
373. Wiriadinata H. A new species of Begonia (Begoniaceae) from Sagea Lagoon, Weda Bay, Halmahera Island, North Moluccas, Indonesia[J]. Reinwardtia, 2012, 13: 263-270.
374. Keim AP. The Pandan flora of Foja-Mamberamo Game Reserve and Baliem Valley, Papua-Indonesia[J]. Reinwardtia, 2012, 13: 271-297.
375. Setyowati FM, Wardah. Keanekaragaman Tumbuhan Obat Masyarakat Talang Mamak di Sekitar Taman Nasional Bukit Tigapuluh, Riau[J]. Biodiversitas, 2007, 8: 228-232.
376. Girmansyah D. Two new species of Begonia (Begoniaceae) from Bukit Tigapuluh National Park, Sumatra, Indonesia[J]. Reinwardtia, 2012, 13: 229-233.
377. Sulistiarini D, Sunarti S, Wiriadinata H. Rekaman Baru Anggrek dari Pulau Wawonii[J]. Biodiversitas, 2007, 8: 83-87.
378. Munawaroh E, Rahayu S. Keragaman jenis Hoya (Apocynaceae) di Taman Nasional Bukit Barisan Selatan (TNBBS) dan konservasinya di Kebun Raya Liwa, Lampung Barat, Lampung[J]. Prosiding Seminar Nasional Masyarakat Biodiversitas Indonesia, 2020, 6: 635-642.

379. Suhardjono, Rugayah. Keanekaragaman Tumbuhan Mangrove di Pulau Sepanjang, Jawa Timur[J]. Biodiversitas, 2007, 8: 130-134.
380. Puspitaningtyas DM, Wawangningrum H. Keanekaragaman Nepenthes di Suaka Alam Sulasih Talang - Sumatera Barat[J]. Biodiversitas, 2007, 8: 152-156.
381. Priatna D, Kartawinata K, Abdulhadi R. Recovery of a lowland dipterocarp forest twenty two years after selective logging at Sekundur, Gunung Leuser National Park, North Sumatra, Indonesia[J]. Reinwardtia, 2004, 12: 237-255.
382. Hartini S. Keragaman Flora dari Monumen Alam Kersik Luway, Kalimantan Timur[J]. Biodiversitas, 2007, 8: 67-72.
383. Rahayu M, Susiarti S, Purwanto Y. Kajian Pemanfaatan Tumbuhan Hutan Non Kayu oleh Masyarakat Lokal di Kawasan Konservasi PT. Wira Karya Sakti Sungai Tapa-Jambi[J]. Biodiversitas, 2006, 8: 73-78.
384. Sulistiarini D. An enumeration of species of Adenoncos (Orchidaceae) from Malesia[J]. Floribunda, 2003, 2: 102-107.
385. Rozak AH, Astutik S, Mutaqien Z, et al. Kekayaan Jenis Pohon di Hutan Taman Nasional Gunung Gede Pangrango, Jawa Barat[J]. Jurnal Penelitian Sosial dan Ekonomi Kehutanan, 2016, 13: 1-14.
386. Hartini S. Tumbuhan Paku di Cagar Alam Sago Malintang, Sumatera Barat dan Aklimatisasinya di Kebun Raya Bogor[J]. Biodiversitas, 2006, 7: 230-236.
387. Slik JWF, Priyono, van Welzen PC. Key to the Macaranga Thou. and Mallotus Lour. Species (Euphorbiaceae) of East Kalimantan, Indonesia[J]. Gardens' Bulletin Singapore, 2000, 52: 11-87.
388. Boyce PC, Bogner J. An Account of Neotenic Species of Rhuphidophora Hassk. (Araceae-Monsteroideae-Monstereae) in New Guinea and Australia[J]. Gardens' Bulletin Singapore, 2000, 52: 89-100.
389. Rahayu S. Keanekaragaman Jenis Hoya (Asclepiadaceae) di Hutan Lindung Bukit Batikap, Kalimantan Tengah[J]. Biodiversitas, 2006, 7: 139-142.
390. Tsukaya H, Okada H. Thismia mullerensis (Burmanniaceae), a new species from Muller Range, Central Kalimantan[J]. Acta Phytotaxonomica et Geobotanica, 2005, 56: 129-133.
391. Tsukaya H, Nakajima M, Okada H. Didymoplexiella cinnabarina (Orchidaceae): a new species from Muller Range, Central Kalimantan, Indonesia[J]. Acta Phytotaxonomica et Geobotanica, 2005, 56: 207-212.
392. Ashton PS. New Tristaniopsis Peter G.Wilson & J.T.Waterh. (Myrtaceae) From Borneo[J]. Gardens' Bulletin Singapore, 2005, 57: 269-278.

393. Okada H. A New Species of Aridarum, Schismatoglottideae, Araceae from the Muller Range, Central Kalimantan, Indonesia[J]. Acta Phytotaxonomica et Geobotanica, 2006, 57: 61-64.
394. Yusuf R, Purwaningsih, Gusman. Komposisi dan Struktur Vegetasi Hutan Alam Rimbo Panti, Sumatera Barat[J]. Biodiversitas, 2005, 6: 266-271.
395. Widjaja EA. New taxa in Indonesian Bamboos[J]. Reinwardtia, 1997, 2: 57-152.
396. Julius A, Suleiman M, Takano A. Five New Species of Plagiostachys (Zingiberaceae) from Borneo[J]. Acta Phytotaxonomica et Geobotanica, 2007, 58: 1-17.
397. Kusumarini N, Ariyanti NS. Keanekaragaman Kemukus di Jawa[J]. Floribunda, 2015, 5: 92-105.
398. Kartawinata K, Samsoedin I, Heriyanto M, et al. A tree species inventory in a one-hectare plot at the Batang Gadis National Park, North Sumatra, Indonesia[J]. Reinwardtia, 2004, 12: 145-157.
399. Arifiani D, Mahyuni R. Keanekaragaman Flora Di Taman Nasional Bukit Barisan Selatan, Provinsi Lampung[J]. Berita Biologi, 2012, 11: 149-160.
400. Puspitaningtyas DM. Studi Keragaman Anggrek di Cagar Alam Gunung Simpang, Jawa Barat[J]. Biodiversitas, 2004, 6: 103-107.
401. Setiadi D. Keanekaragaman Spesies Tingkat Pohon di Taman Wisata Alam Ruteng, Nusa Tenggara Timur[J]. Biodiversitas, 2004, 6: 118-122.
402. Purwaningsih, Yusuf R. Komposisi Jenis dan Struktur Vegetasi Hutan di Kawasan Pakuli, Taman Nasional Lore Lindu, Sulawesi Tengah[J]. Biodiversitas, 2004, 6: 123-128.
403. Witono JR. Keanekaragaman Palem (Palmae) di Gunung Lumut, Kalimantan Tengah[J]. Biodiversitas, 2004, 6: 22-30.
404. Tsukaya H, Okada H. A Color Variation of Epirixanthes Species (Polygalaceae) Found in West Kalimantan, Borneo, Indonesia[J]. Acta Phytotaxonomica et Geobotanica, 2012, 62: 95-97.
405. Andayaningsih D, Chikmawati T, Sulistijorini, et al. Keanekaragaman Tumbuhan Paku Terestrial Di Hutan Kota DKI Jakarta[J]. Berita Biologi, 2013, 12: 297-305.
406. Sihotang VBL. Ethnomedicinal study of the Sundanese people at the Bodogol area, Gede Pangrango Mountain National Park, West Java[J]. Gardens' Bulletin Singapore, 2011, 63: 519-526.
407. Larashati I. Keanekaragaman Tumbuhan dan Populasinya di Gunung Kelud, Jawa Timur[J]. Biodiversitas, 2004, 5: 71-76.

408. Purwaningsih. Sebaran Ekologi Jenis-jenis Dipterocarpaceae di Indonesia[J]. Biodiversitas, 2004, 5: 89-95.
409. Duistermaat H. A taxonomic revision of Amischotolype (Commelinaceae) in Asia[J]. Gardens' Bulletin Singapore, 2012, 64: 51-131.
410. Tsukaya H, Suleiman M, Okada H. Didymoplexiella trichechus (J.J.Sm.) Garay and a New Variety of Didymoplexis cornuta J.J.Sm. (Orchidaceae) in Borneo[J]. Acta Phytotaxonomica et Geobotanica, 2014, 65: 105-110.
411. Uji T. Keanekaragaman dan Potensi Flora di Cagar Alam Muara Kendawangan, Kalimantan Barat[J]. Biodiversitas, 2003, 4: 112-117.
412. Wiriadinata H, Wawo AH. Flora Gunung Kelimutu Dan Gunung Kelibara Taman Nasional Kelimutu, Pulau Flores, Nusa Tenggara Timur[J]. Berita Biologi, 2008, 9: 185-194.
413. Mogea JP. Four new species of Arenga (Palmae) from Indonesia[J]. Reinwardtia, 2004, 12: 181-189.
414. Uji T. Keanekaragaman Dan Potensi Flora Di Gunung Halimun Dan Sekitarnya Di Taman Nasional Gunung Halimun[J]. Berita Biologi, 2002, 6: 1-12.
415. Partomihardjo T, Ismail. Keanekaragaman Flora Cagaral Am Nusabarong, Jember-Jawa Timur[J]. Berita Biologi, 2008, 9: 67-80.
416. Rodda M. Index of names and types of Hoya (Apocynaceae: Asclepiadoideae) of Borneo[J]. Gardens' Bulletin Singapore, 2017, 69: 33-65.
417. Rahayu S, Kusmana C, Abdulhadi R, et al. Distribution of Hoya multiflora Blume at Gunung Gede Pangrango National Park, Indonesia[J]. Journal of Forestry Research, 2010, 7: 42-52.
418. Lasut MT. A new species of Ischaemum from Sulawesi[J]. Reinwardtia, 2006, 12: 257-259.
419. Djufri. Penentuan Pola Distribusi, Asosiasi, dan Interaksi Spesies Tumbuhan Khususnya Padang Rumput di Taman Nasional Baluran, Jawa Timur[J]. Biodiversitas, 2002, 3: 181-188.
420. Keim AP. New species of Pandanus (Pandanaceae) from Kabaena Island, South East Sulawesi, Indonesia[J]. Reinwardtia, 2009, 13: 13-14.
421. Suwardi AB, Navia ZI, Harmawan T. The diversity of wild edible fruit plants and traditional knowledge in West Aceh region, Indonesia[J]. Journal of Medicinal Plants Studies, 2019, 7: 285-290.
422. Rosalia N, Susandarini R. Medicinal plants diversity in Bukit Rimbang Bukit Baling wildlife reserve, Riau, Indonesia[J]. International Journal of Herbal

Medicine, 2020, 8: 33-38.
423. Muzzazinah, Chikmawati T, Ariyanti NS. Indigofera longeracemosa Boiv. ex Baill. in Java[J]. Floribunda, 2015, 5: 106-110.
424. Setyawan AD, Sugiyarto. Keanekaragaman Flora Hutan Jobolarangan Gunung Lawu: 1. Cryptogamae[J]. Biodiversitas, 2001, 2: 115-122.
425. Sutarno, Setyawan AD, Irianto S, et al. Keanekaragaman Flora Hutan Jobolarangan Gunung Lawu: 2. Spermatophyta[J]. Biodiversitas, 2001, 2: 156-162.
426. Kartawinata K, Purwaningsih, Partomihardjo T, et al. Floristics and structure of a lowland dipterocarp forest at Wanariset Samboja, East Kalimantan, Indonesia[J]. Reinwardtia, 2008, 12: 301-323.
427. Widjaja EA, Astuti IP, Arinasa IBK. New species of bamboos (Poaceae-Bambusoideae) from Bali[J]. Reinwardtia, 2004, 12: 199-204.
428. Mulyaningsih T, Ridsdale CE. The Bornean genus Hypobathrum (Rubiaceae). An investigation of its characters and taxonomic status[J]. Reinwardtia, 2002, 12: 95-116.
429. Huzaemah, Mulyaningsih T, Aryanti E. Identifikasi Bambu pada Daerah Aliran Sungai Tiupupus Kabupaten Lombok Utara[J]. Jurnal Biologi Tropis, 2016, 16: 23-36.
430. Afrianto WF, Hikmat A, Widyatmoko D. Komunitas Floristik dan Suksesi Vegetasi Setelah Erupsi 2010 di Gunung Merapi Jawa Tengah[J]. Jurnal Biologi Indonesia, 2016, 12: 265-276.
431. Fathir A, Haikal M, Wahyudi D. Ethnobotanical study of medicinal plants used for maintaining stamina in Madura ethnic, East Java, Indonesia[J]. Biodiversitas, 2021, 22: 386-392.
432. Mansur M. Struktur dan Komposisi Jenis-Jenis Pohon di Taman Nasional Gunung Rinjani bagian Selatan, Lombok, Nusa Tenggara Barat[J]. Jurnal Biologi Indonesia, 2016, 12: 87-98.
433. Yusro F, Mariani Y, Diba F, et al. Inventory of Medicinal Plants for Fever Used by Four Dayak Sub Ethnic in West Kalimantan, Indonesia[J]. Kuroshio Science, 2014, 8: 33-38.
434. Yuzammi. The genus Amorphophallus Blume ex Decaisne (Araceae-Thomsonieae) in Java[J]. Reinwardtia, 2009, 13: 1-12.
435. Girmansyah D. A taxonomic study of Bali and Lombok Begonia (Begoniaceae)[J]. Reinwardtia, 2009, 12: 419-434.

436. Djarwaningsih T. Rekaman Baru Beberapa Jenis Tumbuhan di Jawa[J]. Floribunda, 2010, 4: 15-17.
437. Mirmanto E. Komposisi Floristik dan Struktur Hutan di Pulau Natuna Besar, Kepulaun Natuna[J]. Jurnal Biologi Indonesia, 2014, 10: 201-211.
438. Sunarti S, Rugayah. Keanekaragaman Jenis Gymnospermae di Pulau Wawoni, Sulawesi Tenggara[J]. Jurnal Biologi Indonesia, 2013, 9: 83-92.
439. Sedayu A. A new species of Xanthostemon (Myrtaceae) from Natuna Islands, Indonesia[J]. Reinwardtia, 2009, 12: 447-449.
440. Widjaja EA, Pasaribu N, Hidayat A. A new species Freycinetia (Pandanaceae) from Jambi, Sumatra, Indonesia[J]. Reinwardtia, 2009, 12: 441-442.
441. Rugayah, Sahroni D, Dirman. Annonaceae di Taman Nasional Bogani Nani Wartabone: Studi Pendahuluan Keanekaragamannya[J]. Floribunda, 2011, 4: 40-47.
442. Susiarti S, Purwanto Y, Walujo EB. Medicinal plant diversity in the Tesso Nilo National Park, Riau, Sumatra, Indonesia[J]. Reinwardtia, 2009, 12: 383-390.
443. Wiriadinata H. A new species of Pandanus (Pandanaceae) from Bintuni Bay, West Papua[J]. Reinwardtia, 2009, 12: 443-446.
444. Kartonegoro A. Notes on Sonerila celebica (Melastomataceae)[J]. Floribunda, 2011, 4: 63-64.
445. Sleumer H. Florae Malesianae Precursores XXIII The Genus Rhododendron in Malaysia[J]. Reinwardtia, 1958, 5: 45-231.
446. de Wilde WJJO, Duyfjes BEE. Key and checklist of Xanthophyllum (Polygalaceae) of Borneo[J]. Reinwardtia, 2009, 13: 79-86.
447. Dransfield S. Three new Malesian species of Gramineae[J]. Reinwardtia, 1980, 9: 386-392.
448. Sunarno B. New species of Labisia (Myrsinaceae) from Sumatra[J]. Reinwardtia, 2002, 12: 121-124.
449. Kessler PJA, van Heusden ECH. The Annonaceae of the Balikpapan-Samarinda area, East Kalimantan, Indonesia[J]. Rheedea, 1993, 3: 50-89.
450. Tjitrosoedirdjo SS. Four new taxa of Asteraceae in Sumatra[J]. Reinwardtia, 2002, 12: 125-128.
451. Widuri R, van Welzen P. A revision of the genus Cephalomappa (Euphorbiaceae) in Malesia[J]. Reinwardtia, 1998, 11: 153-184.
452. Rugayah, de Wilde WJJO. New taxa in Malesian Cucurbitaceae[J]. Reinwardtia, 1998, 11: 215-225.

453. Rugayah, de Wilde WJJO. Conspectus of Trichosanthes (Cucurbitaceae) in Malesia[J]. Reinwardtia, 1999, 11: 227-280.
454. Mildawati, Arbain A, Fitrah H. Aspleniaceae of Tandikek Mountain, West Sumatra[J]. Journal of Tropical Life Science, 2013, 3: 202-206.
455. Soepadmo E. Florae Malesianae Praecursores XLIX. Malesian species of Lithocarpus Bl. (Fagaceae)[J]. Reinwardtia, 1970, 8: 197-308.
456. Bremekamp CEB. A revision of the Malaysian Nelsonieae (Scrophulariaceae)[J]. Reinwardtia, 1955, 3: 157-261.
457. Kostermans AJGH. New and critical Malaysian plants-II[J]. Reinwardtia, 1954, 3: 1-25.
458. Kern JH. Notes on Malaysian Cyperaceae-II[J]. Reinwardtia, 1954, 3: 27-66.
459. Leonardo, Usman FH, Yusro F, Kajian Etnobotani Tumbuhan Obat di Desa Sekabuk Kecamatan Sadaniang Kabupaten Pontianak[J]. Jurnal Hutan Lestari, 2013, 1: 32-36.
460. Holttum RE. Tree-ferns of the genus Cyathea in Java[J]. Reinwardtia, 1965, 7: 5-8.
461. Kostermans AJGH. The genus Acioa Aublet (Rosaceae-Chrysobalanoideae) in Malesia[J]. Reinwardtia, 1965, 7: 9-18.
462. Kostermans AJGH. New and critical Malesian plants VII[J]. Reinwardtia, 1965, 7: 19-46.
463. Fosberg FR. Revision of Albizia sect. Pachysperma (Leguminosae-Mimosoideae)[J]. Reinwardtia, 1965, 7: 71-90.
464. Hartono R. A monograph of the genus Schoutenia Korth. (Tiliaceae)[J]. Reinwardtia, 1965, 7: 91-138.
465. Kostermans AJGH. A monograph of the genus Parinari Aubl. (Rosaceae-Chrysobalanoideae) in Asia and the pacific region[J]. Reinwardtia, 1965, 7: 147-213.
466. Kostermans AJGH. Materials for a revision of Lauraceae I[J]. Reinwardtia, 1965, 7: 291-356.
467. Soepadmo E, Jacobs M. Florae Malesianae Praecursores XLVII. census of Malesian Castanopsis (Fagaceae)[J]. Reinwardtia, 1968, 7: 383-410.
468. van Slooten DF. Sertulum Dipterocarpacearum Malayensium-VI[J]. Reinwardtia, 1956, 3: 315-346.
469. Kostermans AJGH. A monograph of the genus Heritiera Aiton (Stercul.) (including Argyrodendron F. v. M. and Tarrietia Bl.)[J]. Reinwardtia, 1959, 4:

465-583.
470. Kostermans AJGH. New and critical Malaysian plants IV[J]. Reinwardtia, 1956, 4: 1-40.
471. Waalkes JVB. Notes on Malaysian Malvaceae-I[J]. Reinwardtia, 1956, 4: 41-68.
472. Erlinawati I. The Diversity of Terrestrial Araceae in Mt. Watuwila Complex, South-East of Sulawesi[J]. Berkala Penelitian Hayati, 2010, 15: 131-137.
473. Syamsiah, Hiola SF, Mu'nisa A, et al. Study on Medicinal Plants Used by the Ethnic Mamuju in West Sulawesi, Indonesia[J]. Journal of Tropical Crop Science, 2016, 3: 42-48.
474. Kurniawan A, Asih NPS, Yuzammi, et al. Studies on the Araceae of the Lesser Sunda Islands I: New distribution records for Alocasia alba[J]. Gardens' Bulletin Singapore, 2013, 65: 157-162.
475. Kurniawan A, Adjie B, Boyce PC. Studies on the Araceae of Sulawesi I: New Taxa of Schismatoglottis and Homalomena, and a Preliminary Checklist and Keys for Sulawesi[J]. Acta Phytotaxonomica et Geobotanica, 2011, 61: 40-50.
476. Ardi WH, Ardhaka IM, Hughes M. Two new species of Begonia (Begoniaceae) from Bali and Lombok[J]. Gardens' Bulletin Singapore, 2013, 65: 135-142.
477. Arifiani D, Basukriadi A, Chikmawati T. Newly described species of Endiandra (Lauraceae) from New Guinea[J]. Reinwardtia, 2012, 13: 341-346.
478. Ashton PS. New Syzygium (Myrtaceae) from Northern Borneo[J]. Kew Bulletin, 2006, 61: 107-144.
479. Polak M. The botanical diversity in the Ayawasi area, Irian Jaya, Indonesia[J]. Biodiversity and Conservation, 2000, 9: 1345-1375.
480. Barfod AS, Heatubun CD. Two new species of Licuala Thunb. (Arecaceae: Coryphoideae) from North Moluccas and Western New Guinea[J]. Kew Bulletin, 2009, 64: 553-557.
481. Bellefroid E, Chaerle P, Leroux O, et al. Additions to the Pteridophyte Flora of Kalimantan, Indonesian Borneo[J]. Kew Bulletin, 2007, 97: 1-18.
482. Bennett JR, Scotland RW. A revision of Strobilanthes (Acanthaceae) in Java[J]. Kew Bulletin, 2003, 58: 1-82.
483. Arbain D, Cannon JR, Afriastini, et al. Survey of some West Sumatran plants for alkaloids[J]. Economic Botany, 1989, 43: 73-78.
484. de Wilde WJJO. The myrmecophilous species of Myristica (Myristicaceae) from New Guinea[J]. Blumea, 1998, 43: 165-182.
485. Adema F. Notes on Malesian Fabaceae (Leguminosae-Papilionoideae). 3. The

genera Dioclea, Luzonia, and Macropsychanthus[J]. Blumea, 1998, 43: 233-239.
486. Hay A. Revision of Homalomena (Araceae-Homalomeneae) in New Guinea, the Bismarck Archipelago and Solomon Islands[J]. Blumea, 1999, 44: 41-71.
487. Simmons CM, de Wilde WJJO. Zehneria subgenus Zehneria (Cucurbitaceae) in Java and Bali[J]. Blumea, 2000, 45: 235-243.
488. Arifiani D. Taxonomic revision of Endiandra (Lauraceae) in Borneo[J]. Blumea, 2001, 46: 99-124.
489. Vink W. Three Bornean species of Madhuca (Sapotaceae)[J]. Blumea, 2001, 46: 193-199.
490. Dowe JL, Ferrero MD. Revision of Calyptrocalyx and the New Guinea species of Linospadix (Linospadicinae: Arecoideae: Arecaceae)[J]. Blumea, 2001, 46: 207-251.
491. Kleijn D, van Donkelaar R. Notes on the taxonomy and ecology of the genus Hoya (Asclepiadaceae) in Central Sulawesi[J]. Blumea, 2001, 46: 457-483.
492. Barker C. Margaritaria (Euphorbiaceae) in Malesia. Blumea, 2001, 46: 505-512.
493. Huynh KL. The genus Freycinetia (Pandanaceae) in New Guinea (Part 4)[J]. Blumea, 2002, 47: 513-536.
494. Bollendorff SM, van Welzen PC, Slik JWF. A taxonomic revision of Mallotus section Polyadenii (Euphorbiaceae)[J]. Blumea, 2000, 45: 319-340.
495. Newman M, Lhuillier A, Poulsen AD. Checklist of the Zingiberaceae of Malesia[J]. Blumea Supplement, 2004, 16: 1-166.
496. Schlechter R. Neue Asclepiadaceen von Sumatra und Celebes[J]. Beihefte zum botanischen Centralblatt, 1916, 34: 1-18.
497. Bramley GLC. Three new species of Callicarpa (Lamiaceae) from Sulawesi[J]. Kew Bulletin, 2012, 67: 213-223.
498. Bramley GLC, Cronk QCB. The Cyrtandra (Gesneriaceae) Species of Mount Kerinci, Sumatra[J]. Harvard Papers in Botany, 2003, 7: 407-421.
499. Wilkie P, Argent G, Cambell E, et al. The diversity of 15 ha of lowland mixed dipterocarp forest, Central Kalimantan[J]. Biodiversity and Conservation, 2004, 13: 695-708.
500. Caniago I, Stephen FS. Medicinal plant ecology, knowledge and conservation in Kalimantan, Indonesia[J]. Economic Botany, 1998, 52: 229-250.
501. Coode MJE. Elaeocarpus for Flora Malesiana: the Coilopetalum group in the Lesser Sunda Islands[J]. Kew Bulletin, 2001, 56: 875-883.
502. Coode MJE. Elaeocarpus for Flora Malesiana: new taxa and understanding in the

Ganitrus group[J]. Kew Bulletin, 2010, 65: 355-399.
503. Widjaja EA, Karsono. Keanekaragaman Bambu di Pulau Sumba[J]. Biodiversitas, 2004, 6: 95-99.
504. Uji T. Keanekaragaman dan Potensi Flora di Suaka Margasatwa Buton Utara, Sulawesi Tenggara[J]. Biodiversitas, 2005, 6: 205-211.
505. Windadri FI, Rahayu M, Uji T, et al. Pemanfaatan Tumbuhan sebagai Bahan Obat oleh Masyarakat Lokal Suku Muna di Kecamatan Wakarumba, Kabupaten Muna, Sulawesi Tenggara[J]. Biodiversitas, 2006, 7: 333-339.
506. Puspitaningtyas DM, Wawangningrum H. Keanekaragaman Nepenthes di Suaka Alam Sulasih Talang Sumatera Barat[J]. Biodiversitas, 2007, 8: 152-156.
507. Ariyanti EE, Pa'i. Inventarisasi Anggrek di Kabupaten Sintang, Kalimantan Barat[J]. Biodiversitas, 2008, 9: 21-24.
508. Sunarti S, Hidayat A, Rugayah. Keanekaragaman Tumbuhan di Hutan Pegunungan Waworete, Kecamatan Wawonii Timur, Pulau Wawonii, Sulawesi Tenggara[J]. Biodiversitas, 2008, 9: 194-198.
509. Fici S. Capparis kebarensis, a new species of Capparaceae from Papua Barat, Indonesia[J]. Kew Bulletin, 2012, 67: 739-741.
510. Rustiami H. Daemonorops melanochaetes Blume, A New Record of Rattan for Bali[J]. Floribunda, 2016, 5: 126-128.
511. Foreman DB. New species of Helicia Lour. (Proteaceae) from the Vogelkop Peninsula, Irian Jaya[J]. Kew Bulletin, 1998, 53: 669-681.
512. Wardani W, Adjie B. Checklist of Pteridophyte Flora of Enggano Island[J]. Floribunda, 2017, 5: 209-219.
513. Girmansyah D. Begonia sinuata Meisn., A New Record for Indonesia[J]. Floribunda, 2014, 5: 27-29.
514. Mogea JP. Preliminary Study on the Palm Flora of the Lore Lindu National Park, Central Sulawesi, Indonesia[J]. Biotropia, 2002, 18: 1-20.
515. Tjitrosoedirdjo SS. Notes on the Asteraceae of Sumatera[J]. Biotropia, 2002, 19: 65-84.
516. Hartley TG, Craven LA. A Revision of the Papuasian Species of Acmena (Myrtaceae)[J]. Journal of the Arnold Arboretum, 1977, 58: 325-342.
517. Hicks D. The racemose Ilex (Aquifoliaceae) of New Guinea[J]. Kew Bulletin, 2006, 61: 537-547.
518. Hopkins HCF. The Indo-Pacific species of Parkia (Leguminosae: Mimosoideae)[J]. Kew Bulletin, 1994, 49: 181-234.

519. Hovenkamp PH, de Joncheere GJ. Additions to the fern flora of Sulawesi[J]. Blumea, 1988, 33: 395-409.
520. Hughes M, Girmansyah D, Ardi WH, et al. Seven New Species of Begonia from Sumatra[J]. Gardens' Bulletin Singapore, 2009, 61: 29-44.
521. Hughes M, Girmansyah D. A revision of Begonia sect. Sphenanthera (Hassk.) Warb. from Sumatra[J]. Gardens' Bulletin Singapore, 2011, 62: 27-39.
522. Bramley GL, Pennington RT, Zakaria R, et al. Assembly of tropical plant diversity on a local scale: Cyrtandra (Gesneriaceae) on Mount Kerinci, Sumatra[J]. Biological Journal of the Linnean Society, 2004, 81: 49-62.
523. Silveira P, Schuiteman A, Vermeulen JJ, et al. The orchids of Timor: checklist and conservation status[J]. Botanical Journal of the Linnean Society, 2008, 157: 197-215.
524. Silalahi M. Keanekaragaman dan distribusi tumbuhan bermanfaat di pekarangan kampus Universitas Kristen Indonesia (UKI) Cawang, Jakarta Timur[J]. Jurnal Biologi, 2016, 20: 75-82.
525. de Kok R, Rusea G, Latiff A. The genus Teijsmanniodendron Koord. (Lamiaceae)[J]. Kew Bulletin, 2009, 64: 587-625.
526. de Kok R. The genus Premna L. (Lamiaceae) in the Flora Malesiana area[J]. Kew Bulletin, 2013, 68: 55-84.
527. de Kok R. The genus Vitex (Labiatae) in the Flora Malesiana region, excluding New Guinea[J]. Kew Bulletin, 2008, 63: 17-40.
528. Merrill ED, Perry LM. Plantae Papuanae Archboldianae, X[J]. Journal of the Arnold Arboretum, 1942, 23: 383-416.
529. Merrill ED, Perry LM. Plantae Papuanae Archboldianae, XI[J]. Journal of the Arnold Arboretum, 1943, 24: 34-59.
530. Monro AK. Three new species, and three new names in Pilea (Urticaceae) from New Guinea. Contributions to the Flora of Mt Jaya XV[J]. Kew Bulletin, 2004, 59: 573-579.
531. Mitani M, Watanabe K, Gurmaya KJ, et al. Plant species list from the Pananjung Pangandaran Nature Reserve, west Java, Indonesia, sampled in the El Niño-Southern Oscillation year of 1997[J]. Humans and Nature, 2009, 20: 113-120.
532. Vermeulen JJ. A taxonomic revision of Bulbophyllum, sections Adelopetalum, Lepanthanthe, Macrouris, Pelma, Peltopus, and Uncifera (Orchidaceae)[J]. Orchid Monographs, 1993, 7: 1-191.

533. Ormerod P. Papuasian Orchid Studies, 2[J]. Austrobaileya, 2005, 7: 183-203.
534. Turner IM. A catalogue of the Annonaceae of Borneo[J]. Phytotaxa, 2011, 36: 1-120.
535. Pereira JT. Five new species of Payena (Sapotaceae) and notes on the genus in Borneo[J]. Kew Bulletin, 1997, 52: 903-922.
536. Perry LM. Plantae Papuanae Archboldianae, XXI* the Papuasian Species of Macaranga[J]. Journal of the Arnold Arboretum, 1953, 34: 191-257.
537. Ranlund Å. Structure and tree diversity of lowland limestone forest on Seram Island, Indonesia[D]. Uppsala: Swedish University of Agricultural Sciences, 2011.
538. Kabelen F, Warpur M. Struktur, Komposisi Jenis Pohon dan Nilai Ekologi Vegetasi Kawasan Hutan di Kampung Sewan Distrik Sarmi, Kabupaten Sarmi[J]. Jurnal Biologi Papua, 2009, 1: 72-80.
539. Boyce PC. The Genus Rhaphidophora Hassk. (Araceae-Monsteroideae-Monstereae) in the Southern and Western Indonesian Archipelago[J]. Gardens' Bulletin Singapore, 2000, 52: 101-183.
540. Milliken W, Proctor J. Montane forest in the Dumoga Bone National Park, North Sulawesi[J]. Edinburgh Journal of Botany, 1999, 56: 449-458.
541. Mendum M. Notes on Aeschynanthus (Gesneriaceae) from Seram[J]. Edinburgh Journal of Botany, 1998, 55: 359-365.
542. Saw LG. A revision of Licuala (Arecaceae, Coryphoideae) in Borneo[J]. Kew Bulletin, 2012, 67: 577-654.
543. Scott AJ. Decaspermum (Myrtaceae) in New Guinea[J]. Kew Bulletin, 1985, 40: 149-165.
544. Airy Shaw HK. The Euphorbiaceae of Central Malesia (Celebes, Moluccas. Lesser Sunda Is.)[J]. Kew Bulletin, 1982, 37: 1-40.
545. Airy Shaw HK. The Euphorbiaceae of Sumatra[J]. Kew Bulletin, 1981, 36: 239-374.
546. Airy Shaw HK. Notes on Malesian and other Asiatic Euphorbiaceae[J]. Kew Bulletin, 1972, 27: 3-93.
547. Airy Shaw HK. Notes on Malesian and other Asiatic Euphorbiaceae[J]. Kew Bulletin, 1978, 32: 361-418.
548. Shimizu T, Utami N. Three new species of Impatiens (Balsaminaceae) added to Flora Malesiana[J]. Kew Bulletin, 1997, 52: 435-442.
549. Snow N, Craven LA. Five new species of Syzygium (Myrtaceae) from New

Guinea[J]. Harvard Papers in Botany, 2010, 15: 123-136.
550. Yeng WS. Studies on Schismatoglottideae (Araceae) of Borneo XV: A Second Species of Bakoa from Indonesian Borneo[J]. Acta Phytotaxonomica et Geobotanica, 2011, 61: 127-129.
551. Kurniawan A, Boyce PC. Studies on the Alocasia Schott (Araceae-Colocasieae) of Borneo II: Alocasia baginda, a New Species from Eastern Kalimantan, Indonesian Borneo[J]. Acta Phytotaxonomica et Geobotanica, 2011, 60: 123-126.
552. Nurainas N, Arbain D. A new species and a new record of Zingiberaceae from Sumatra, Indonesia[J]. Taiwania, 2017, 62: 294-298.
553. Rodda M, Rahayu S. Two new species of Hoya (Apocynaceae, Asclepiadoideae) from Sulawesi, Indonesia[J]. Taiwania, 2020, 65: 209-215.
554. Takeuchi W. New taxa from the Mamberamo river of Papua province, Indonesia: Ardisia lammersiana (Myrsinaceae) and Psychotria leptothyrsa var. defretesiana (Rubiaceae)[J]. Harvard Papers in Botany, 2009, 14: 173-181.
555. Takeuchi W, Arifiani D. A synopsis of new plant distributional records from the Foja mountains of Papua province, Indonesia[J]. Harvard Papers in Botany, 2010, 15: 41-50.
556. Utami N. Three new species of Impatiens (Balsaminaceae) from Sumatra, Indonesia[J]. Kew Bulletin, 2012, 67: 731-737.
557. Utteridge TMA. The subalpine members of Pittosporum (Pittosporaceae) from Mt Jaya, New Guinea. Contributions to the Flora of Mt Jaya, II[J]. Kew Bulletin, 2000, 55: 699-710.
558. Heatubun CD. Palms on the Nickel Island: An expedition to Gag Island, Western New Guinea[J]. Palms, 2014, 58: 115-134.
559. Wood JRI, Scotland RW. New and little-known species of Strobilanthes (Acanthaceae) from India and South East Asia[J]. Kew Bulletin, 2009, 64: 3-47.
560. Hendrian. Revision of Ochrosia (Apocynaceae) in Malesia[J]. Blumea, 2004, 49: 101-128.
561. Adema F. Notes on Malesian Fabaceae (Leguminosae-Papilionoideae). 9. The genus Paraderris[J]. Blumea, 2003, 48: 129-144.
562. Adema F. Notes on Malesian Fabaceae (Leguminosae-Papilionoideae). 10. The genus Alysicarpus[J]. Blumea, 2003, 48: 145-152.
563. Adema F. Notes on Malesian Fabaceae (Leguminosae-Papilionoideae). 11. The genus Derris[J]. Blumea, 2003, 48: 393-419.
564. Adema F. Notes on Malesian Fabaceae (Leguminosae-Papilionoideae). 13. The

genus Inocarpus[J]. Blumea, 2007, 52: 401-407.

565. Adema F, Ohashi H, Sunarno B. Notes on Malesian Fabaceae (Leguminosae-Papilionoideae). 17. The genus Dalbergia[J]. Blumea, 2016, 61: 186-206.

566. Agustini V, Zebua LI, Wenda N. Short Communication: Inventory of native orchids in Makki Sub-District, Lanny Jaya, Papua, Indonesia[J]. Biodiversitas, 1970, 17: 301-305.

567. Ahmad KS, Jadid N, Kurniawan E, et al. An ethnobotanical study of medicinal plants used by the Tengger tribe in Ngadisari village, Indonesia[J]. PLoS One, 2020, 15: e0235886.

568. Ahmad RPP, Zulfadli Z, Rahayu SRI, et al. One new species and a new record of Hoya (Apocynaceae, Asclepiadoideae) from Sulawesi, Indonesia[J]. Phytotaxa, 2021, 502: 79-85.

569. Almulqu AA, Arpornpong N, Boonyanuphap J. Tree species composition and structure of dry forest in Mutis Timau Protected Forest Management Unit of East Nusa Tenggara, Indonesia[J]. Biodiversitas, 2018, 19: 496-503.

570. Amarullah ET, Trizelia T, Yaherwandi Y, et al. Diversity of plant species in paddy ecosystem in West Sumatra, Indonesia[J]. Biodiversitas, 2017, 18: 1218-1225.

571. Ardhana IPG, Rimbawan IMGS, Cahyo PN, et al. The distribution of vertical leaves and leaves biomass on ten mangrove species at Ngurah Rai Forest Park, Denpasar, Bali, Indonesia[J]. Biodiversitas, 2018, 19: 918-926.

572. Ardi WH, Ardaka IM, Hartutiningsih MS, et al. Two new species of Begonia (Begoniaceae) from Sulawesi, Indonesia[J]. Edinburgh Journal of Botany, 2014, 71: 259-268.

573. Ardi WH, Girmansyah D, Hughes M. Begonia Robii, a new species of Begonia from Lima Puluh Kota, West Sumatra[J]. Reinwardtia, 2021, 20: 37-41.

574. Ardi WH, Hughes M. Two new species of Begonia from Sumatra[J]. Edinburgh Journal of Botany, 2018, 75: 297-304.

575. Ardi WH, Thomas DC. Studies on Begonia (Begoniaceae) of the Moluccas II: a new species from Seram, Indonesia[J]. Gardens' Bulletin Singapore, 2015, 67: 297-303.

576. Ardiyani M, Newman MF, Poulsen AD. A new species of Zingiber (Zingiberaceae) east of Wallace's Line[J]. Gardens' Bulletin Singapore, 2017, 69: 189-199.

577. Argent G. Vaccinium nagamasu (Ericaceae), a new species (sect. Bracteata) from

Sumatra, Indonesia[J]. Edinburgh Journal of Botany, 2011, 68: 419-422.
578. Argent G. A contribution to the study of the genus Diplycosia (Ericaceae) in Sulawesi, Indonesia[J]. Edinburgh Journal of Botany, 2014, 71: 83-115.
579. Argent G. Vaccinium utteridgei (Ericaceae), a new species (sect. Bracteata) from Indonesian New Guinea[J]. Edinburgh Journal of Botany, 2014, 71: 189-192.
580. Argent G. Rigiolepis and Vaccinium (Ericaceae) in Borneo[J]. Edinburgh Journal of Botany, 2018, 76: 55-172.
581. Argent G, Widjaja EA. Diplycosia mekonggaensis (Ericaceae, Gaultherieae), a new species from Sulawesi, Indonesia[J]. Edinburgh Journal of Botany, 2015, 72: 239-242.
582. Argent G, Wilkie P. Six new species of Vaccinium (Ericaceae) from New Guinea[J]. Edinburgh Journal of Botany, 2020, 77: 439-453.
583. Armstrong KE. A revision of the Asian-Pacific species of Manilkara (Sapotaceae)[J]. Edinburgh Journal of Botany, 2013, 70: 7-56.
584. Asih NPS, Kurniawan A, Boyce PC. Studies on Homalomeneae (Araceae) of Borneo XII-Homalomena tirtae, a new species from Kalimantan Timur, Indonesian Borneo, and notes on theHomalomenaBorneensis Complex[J]. Willdenowia, 2012, 42: 241-246.
585. Atkins HJ, Heatubun CD, Galloway L, et al. Two new species, Cyrtandra bungahijau and C. vittata, and notes on Cyrtandra (Gesneriaceae) from Yapen Island, Indonesia[J]. Kew Bulletin, 2019, 74: 29.
586. Atkins HJ, Kartonegoro A. A taxonomic revision of Cyrtandra (Gesneriaceae) in Sulawesi, Indonesia[J]. Edinburgh Journal of Botany, 2021, 78: 1-122.
587. Atmaja MB, Tirta IG. Notes on the orchids of Bali, Indonesia: six new species records[J]. Gardens' Bulletin Singapore, 2019, 71: 421-427.
588. Barkman TJ, Emoi BE, Repin R. The genus Balanophora (Balanophoraceae) in Sabah, Malaysia[J]. Blumea, 2003, 48: 465-474.
589. Batoro J. An Ethnobotanical Survey for Tropical Sand Dune Support Greenbelt International Airport Yogyakarta (NYIA) Glagah Village, District Temon, Kulon Progo, Yogyakarta Indonesia[J]. Journal of Coastal Zone Management, 2017, 20: 452.
590. Berg CC. Flora Malesiana precursor for the treatment of Moraceae 2: Ficus subgenus Pharmacosycea section Oreosycea[J]. Blumea, 2003, 48: 289-301.
591. Berg CC. Flora Malesiana precursor for the treatment of Moraceae 3: Ficus subgenus Ficus[J]. Blumea, 2003, 48: 529-550.

592. Berg CC. Flora Malesiana precursor for the treatment of Moraceae 4: Ficus subgenus Synoecia[J]. Blumea, 2003, 48: 551-571.
593. Berg CC. Flora Malesiana precursor for the treatment of Moraceae 5: Ficus subgenus Sycidium[J]. Blumea, 2003, 48: 573-597.
594. Berg CC. Flora Malesiana precursor for the treatment of Moraceae 6: Ficus subgenus Sycomorus[J]. Blumea, 2004, 49: 155-200.
595. Berg CC. Flora Malesiana precursor for the treatment of Moraceae 7: Ficus subgenus Urostigma[J]. Blumea, 2004, 49: 463-480.
596. Berg CC. Flora Malesiana precursor for the treatment of Moraceae 8: Other genera than Ficus[J]. Blumea, 2005, 50: 535-550.
597. Berg CC. Two New Guinean Ficus species (Moraceae): One new and the other with an emended description[J]. Blumea, 2007, 52: 291-294.
598. Berg CC. Ficus capillipes (Moraceae), new for Malesia (Sumatra)[J]. Blumea, 2008, 53: 325-327.
599. Berg CC. Ficus glandulifera and allied species (Moraceae), including a new one from Borneo and Celebes[J]. Blumea, 2008, 53: 319-323.
600. Berg CC. Corrective notes on the Malesian members of the genus Ficus (Moraceae)[J]. Blumea, 2011, 56: 161-164.
601. Berg CC. Seven new Malesian species of Ficus (Moraceae)[J]. Blumea, 2012, 57: 147-157.
602. Berg CC, Chantarasuwan B. A study on the taxonomy of some stoloniflorous species of Ficus subsection Sycocarpus (Moraceae) in Thailand and Malesia[J]. Blumea, 2007, 52: 313-326.
603. Berg CC, Culmsee H. Ficus schwarzii redefined and two new species of Ficus (Moraceae) from Sulawesi (Indonesia) described[J]. Blumea, 2011, 56: 265-269.
604. Beukema H, Danielsen F, Vincent G, et al. Plant and bird diversity in rubber agroforests in the lowlands of Sumatra, Indonesia[J]. Agroforestry Systems, 2007, 70: 217-242.
605. Brambach F, Leuschner C, Tjoa A, et al. Diversity, endemism, and composition of tropical mountain forest communities in Sulawesi, Indonesia, in relation to elevation and soil properties[J]. Perspectives in Plant Ecology, Evolution and Systematics, 2017, 27: 68-79.
606. Brambach F, Nooteboom HP, Culmsee H. Magnolia sulawesiana described, and a key to the species of Magnolia (Magnoliaceae) occurring in Sulawesi[J]. Blumea, 2013, 58: 271-276.

607. Bramley GLC. Revision of Cyrtandra section Dissimiles (Gesneriaceae)[J]. Blumea, 2005, 50: 163-189.
608. Briggs M. A new name in New Guinea Saurauia Willd. (Actinidiaceae)[J]. Kew Bulletin, 2015, 70: 51.
609. Briggs M, Utteridge TMA. An updated and revised description of Timonius grandifolius (Rubiaceae: Guettardeae) and a new character observation for the tribe Guettardeae. Contributions to the Flora of Mt Jaya, XIX[J]. Kew Bulletin, 2014, 69: 9512.
610. Budiarti M, Maruzy A, Mujahid R, et al. The use of antimalarial plants as traditional treatment in Papua Island, Indonesia[J]. Heliyon, 2020, 6: e05562.
611. Cahen D, Stenn KS, Utteridge TMA. A revision of the genus Gouania (Rhamnaceae) in the Philippines and Sundaland[J]. Kew Bulletin, 2020, 75: 25.
612. Cahyaningsih R, Magos Brehm J, Maxted N. Setting the priority medicinal plants for conservation in Indonesia[J]. Genetic Resources and Crop Evolution, 2021, 68: 2019-2050.
613. Cahyanto T, Efendi M, Ramdan DM. Structure and composition of trees in Mount Tilu Nature Reserve, West Java, Indonesia[J]. Biodiversitas, 2020, 21: 2674-2680.
614. Cahyanto TRI, Efendi M, Shofara RM, et al. Short Communication: Floristic survey of vascular plant in the submontane forest of Mt. Burangrang Nature Reserve, West Java, Indonesia[J]. Biodiversitas, 2019, 20: 2197-2205.
615. Cámara-Leret R, Frodin DG, Adema F, et al. New Guinea has the world’s richest island flora[J]. Nature, 2020, 584: 579-583.
616. Cámara-Leret R, Ridder-Numan JWA, Veldkamp JF. Revision of Heteroblemma gen. nov. (Dissochaeteae-Melastomataceae) from Malesia and Vietnam[J]. Blumea, 2013, 58: 229-240.
617. Cellinese N. Revision of the genus Phyllagathis (Melastomataceae: Sonerileae) II. the species in Borneo and Natuna Island[J]. Blumea, 2003, 48: 69-97.
618. Chambers TC, Wilson PG. A revision of Blechnum vulcanicum (Blume) Kuhn and related taxa (Blechnaceae) in Malesia and Oceania[J]. Telopea, 2019, 22: 41-59.
619. Cheek M. Nepenthes (Nepenthaceae) of Halmahera, Indonesia[J]. Blumea, 2015, 59: 215-225.
620. Cheek M, Jebb M. A new section in Nepenthes (Nepenthaceae) and a new species from Sulawesi[J]. Blumea, 2016, 61: 59-62.

621. Cheek M, Jebb M, Murphy B, et al. Nepenthes section Insignes in Indonesia, with two new species[J]. Blumea, 2018, 62: 174-178.
622. Cheek M, Wanma J, Jitmau M, et al. Seringia (Byttneriaceae/Malvaceae-Byttnerioideae) new to Southeast Asia and S. botak endangered in Indonesian New Guinea grassland and savannah[J]. Blumea, 2018,63: 150-156.
623. Chung RCK, Soepadmo E. Diplodiscus latifii (Malvaceae-Brownlowioideae), a new species from Sabah, Malaysia[J]. PhytoKeys, 2020, 161: 99-106.
624. Coode MJE. Elaeocarpus for Flora Malesiana: the Monocera group in western Malesia[J]. Kew Bulletin, 2014, 69: 9487.
625. Coode MJE. Elaeocarpus for Flora Malesiana: five new taxa[J]. Kew Bulletin, 2019, 74: 36.
626. Coritico FP, Amoroso VB, Lehnert M. New records, names and combinations of scaly tree ferns (Cyatheaceae) in eastern Malesia[J]. Blumea, 2017, 62: 92-96.
627. Craven LA. Malesian and Australian tournefortia transferred to Heliotropium and notes on delimitation of Boraginaceae[J]. Blumea, 2005, 50: 375-381.
628. Craven LA. Studies in Papuasian Syzygium (Myrtaceae): 1. Subgenus Perikion revised[J]. Blumea, 2019, 64: 115-122.
629. Culmsee H, Pitopang R. Tree diversity in sub-montane and lower montane primary rain forests in Central Sulawesi[J]. Blumea, 2009, 54: 119-123.
630. Culmsee H, Pitopang R, Mangopo H, et al. Tree diversity and phytogeographical patterns of tropical high mountain rain forests in Central Sulawesi, Indonesia[J]. Biodiversity and Conservation, 2011, 20: 1103-1123.
631. Damayanti I, Nurbambang A, Soeprobowati TR. Plant diversity of Petungkriyono Forest of Dieng Plateau, Central Java, Indonesia[J]. Biodiversitas, 2021, 22: 3497-3507.
632. Damayanto IPGP, Rustiami H, Miftahudin, et al. A synopsis of Bambusoideae (Poaceae) in Lombok, Indonesia[J]. Biodiversitas, 2020, 21: 4489-4500.
633. Darmawati IAP, Rai IN, Dwiyani R, et al. Short Communication: The diversity of wild Dendrobium (Orchidaceae) in Central Bali, Indonesia[J]. Biodiversitas, 2018, 19: 1110-1116.
634. Davis AP, Ruhsam M. Five new combinations and one new name in Rubiaceae from South-East Asia[J]. Blumea, 2005, 50: 575-578.
635. de Wilde WJJO, Duyfjes BEE. Redefinition of Zehneria and four new related genera (Cucurbitaceae), with an Enumeration of the Australasian and Pacific

species[J]. Blumea, 2006, 51: 1-88.

636. de Wilde WJJO, Duyfjes BEE. The subtribe Thladianthinae (Cucurbitaceae) in Indochina and Malesia[J]. Blumea, 2006, 51: 493-518.

637. de Wilde WJJO, Duyfjes BEE. Gynostemma (Cucurbitaceae) in Thailand and Malesia[J]. Blumea, 2007, 52: 263-280.

638. de Wilde WJJO, Duyfjes BEE. Ammannia (Lythraceae) in Malesia[J]. Blumea, 2014, 59: 11-18.

639. de Wilde WJJO, Duyfjes BEE. Lagerstroemia (Lythraceae) in Malesia[J]. Blumea, 2014, 59: 113-122.

640. de Witte LC, Vermeulen JJ. The Bulbophyllum species attributed to section Hymenobractea (Orchidaceae)[J]. Blumea, 2010, 55: 80-85.

641. Dennehy Z, Cámara-Leret R. Quantitative ethnobotany of palms (Arecaceae) in New Guinea[J]. Gardens' Bulletin Singapore, 2019, 71: 321-364.

642. de Wilde WJJO, Duyfjes BEE. Taxonomy of Alangium section Conostigma (Alangiaceae)[J]. Blumea, 2017, 62: 29-46.

643. Djamaluddin R. The mangrove flora and their physical habitat characteristics in Bunaken National Park, North Sulawesi, Indonesia[J]. Biodiversitas, 2018, 19: 1303-1312.

644. Djarwaningsih T. Revision of Pimelodendron (Euphorbiaceae) in Malesia[J]. Blumea, 2004, 49: 407-423.

645. Djufri, Wardiah, Muchlisin ZA. Plants diversity of the deforested peat-swamp forest of Tripa, Indonesia[J]. Biodiversitas, 2016, 17: 372-376.

646. Droop AJ, Newman MF. A revision of Amomum (Zingiberaceae) in Sumatra[J]. Edinburgh Journal of Botany, 2014, 71: 193-258.

647. Eddy S, Ridho M, Iskandar I, et al. Species composition and structure of degraded mangrove vegetation in the Air Telang Protected Forest, South Sumatra, Indonesia[J]. Biodiversitas, 2019, 20: 2119-2127.

648. Efendi M, Lailaty IQ, Nudin, et al. Komposisi dan keanekaragaman flora di Gunung Pesagi, Sumatera[J]. Prosiding Seminar Nasional Masyarakat Biodiversitas Indonesia, 2016, 2: 198-207.

649. Fijridiyanto IA, Smets E, Arifiani D. Taxonomic revision of Dehaasia (Lauraceae) in Sumatra[J]. Blumea, 2020, 65: 167-175.

650. Gagul JN, Sands MJS, Gideon O, et al. A revision of Begonia sect. Symbegonia on New Guinea[J]. Edinburgh Journal of Botany, 2018, 75: 127-159.

651. Ganesan SK, Middleton DJ, Wilkie P. A revision of Pterospermum (Malvaceae:

Dombeyoideae) in Malesia[J]. Edinburgh Journal of Botany, 2019, 77: 161-241.

652. Gardner RO. Piper (Piperaceae) in New Guinea: the non-climbing species[J]. Blumea, 2003, 48: 47-68.

653. Gardner RO. Piper (Piperaceae) in New Guinea: the climbing species[J]. Blumea, 2013, 57: 275-294.

654. Girmansyah D. Three new species of Begonia (Begoniaceae) from Sumbawa Island, Indonesia[J]. Gardens' Bulletin Singapore, 2016, 68: 77-86.

655. Girmansyah D. Two new species of Begonia (Begoniaceae) from Long Duhung, Berau Regency, East Kalimantan, Borneo Island, Indonesia[J]. Kew Bulletin, 2016, 72: 3.

656. Girmansyah D, Susanti R. Two new species of Begonia (Begoniaceae) from Borneo[J]. Kew Bulletin, 2015, 70:19.

657. Goldblatt P. Systematics of Patersonia (Iridaceae, Patersonioideae) in the Malesian Archipelago[J]. Annals of the Missouri Botanical Garden, 2012, 98: 514-523.

658. Hambali GG, Sunarti S, Low YW. Syzygium jiewhoei (Myrtaceae), a new endemic tree from Western New Guinea, Indonesia[J]. Gardens' Bulletin Singapore, 2017, 69: 201-210.

659. Hartianti D, Budipramana K. Traditional antidiabetic plants from Indonesia[J]. Ethnobotany Research and Applications, 2020, 19: 34.

660. Hartini S. Orchids Diversity in the Sicikeh-Cikeh Forest, North Sumatra, Indonesia[J]. Biodiversitas, 2019, 20: 1087-1096.

661. Hastuti H, Purnomo P, Sumardi I, et al. Diversity wild banana species (Musa spp.) in Sulawesi, Indonesia[J]. Biodiversitas, 2019, 20: 824-832.

662. Heatubun CD, Barfod AS. Two new species of Licuala (Arecaceae; Coryphoideae) from western New Guinea[J]. Blumea, 2008, 53: 429-434.

663. Heatubun CD, Zona S, Baker WJ. Three new genera of arecoid palm (Arecaceae) from eastern Malesia[J]. Kew Bulletin, 2014, 69: 9525.

664. Heijkoop M, van welzen PC. A revision of the genus Actephila (Phyllanthaceae) in the Malesian region[J]. Blumea, 2017, 62: 7-25.

665. Hendrayana Y, Widodo P, Kusmana C, et al. Short Communication: Diversity and distribution of figs (Ficus spp.) across altitudes in Gunung Tilu, Kuningan, West Java, Indonesia[J]. Biodiversitas, 2019, 20: 1568-1574.

666. Hidayat RS, Cahyaningsih R. Useful plants from wolomeze protected forest, Ngada District, Florest, East Nusa Tenggara[J]. Biodiversitas, 2017, 3: 56-61.

667. Hovenkamp P, Ho B-C. A revision of the fern genus Oleandra (Oleandraceae) in Asia[J]. PhytoKeys, 2012, 11: 1-37.
668. Hsu S-C, Gravendeel B, de Vogel EF. Taxonomic revision of Geesinkorchis (Coelogyninae; Epidendroideae; Orchidaceae)[J]. Blumea, 2005, 50: 505-517.
669. Hughes M. Four new species of Begonia (Begoniaceae) from Sulawesi[J]. Edinburgh Journal of Botany, 2006, 63: 191-199.
670. Hughes M, Girmansyah D, Ardi WH. Further discoveries in the ever-expanding genus Begonia (Begoniaceae): fifteen new species from Sumatra[J]. European Journal of Taxonomy, 2015, 167: 1-40.
671. Hughes M, Girmansyah D, Randi A, et al. Eleven new records, three new species and an updated checklist of Begonia from Kalimantan, Indonesia[J]. Gardens' Bulletin Singapore, 2020, 72: 33-58.
672. Husodo T, Palabbi SD, Abdoellah OS, et al. Short communication: Seagrass diversity and carbon sequestration: Case study on Pari Island, Jakarta Bay, Indonesia[J]. Biodiversitas, 2017, 18: 1596-1601.
673. Fawzi NI, Novianto A, Supianto A, et al. Jenis Pohon Target Dan Aktivitas Pembalakan Liar Di Taman Nasional Gunung Palung[J]. Jurnal Penelitian Hutan dan Konservasi Alam, 2020, 17: 49-63.
674. Irischer E. Neue Begoniaceen, von O. Beccari in Malesien gesammelt[J]. Webbia, 1954, 9: 469-509.
675. Iskandar BS, Iskandar J, Partasasmita R, et al. Various medicinal plants traded in the village market of Karangwangi Village, Southern Cianjur, West Java, Indonesia[J]. Biodiversitas, 2020, 21: 4440-4456.
676. Ismaini L, Lailati M, Rustandi, et al. Analisis komposisi dan keanekaragaman tumbuhan di Gunung Dempo, Sumatera Selatan[J]. Prosiding Seminar Nasional Masyarakat Biodiversitas Indonesia, 2015, 1: 1397-1402.
677. Isnaini Y, Magandhi M, Sahromi. Exploration of flora diversity in Sebangka Island for Batam Botanic Gardens[J]. AIP Conference Proceedings, 2019, 2120: 040025.
678. Jacobsen N. The Cryptocoryne (Araceae) of Borneo[J]. Nordic Journal of Botany, 2008, 5: 31-50.
679. Jacobsen N, Bastmeijer JD, Edwards PJ, et al. A new variety of Cryptocoryne versteegii (Araceae) from Irian Jaya Tengah, Indonesia[J]. Willdenowia, 2014, 44: 385-391.
680. Jebb M, Prance GT. Five new species of Barringtonia (Lecythidaceae) from

Papua New Guinea[J]. Blumea, 2011, 56: 105-112.

681. Johnson DM, Murray NA. A contribution to the systematics of Xylopia (Annonaceae) in Southeast Asia[J]. Gardens' Bulletin Singapore, 2015, 67: 361-386.

682. Joyce EM, Thiele KR, Slik FJW, et al. Checklist of the vascular flora of the Sunda-Sahul Convergence Zone[J]. Biodiversity Data Journal, 2020, 8: e51094.

683. Julia S, Kiew R. Eight new Begonia (Begoniaceae) species from the Lanjak Entimau Wildlife Sanctuary and Batang Ai National Park, Sarawak, Borneo[J]. Gardens' Bulletin Singapore, 2016, 68: 257-277.

684. Junaedi DI, Mutaqien Z. Diversity of tree communities in Mount Patuha region, West Java[J]. Biodiversitas, 2009, 11: 75-81.

685. Juswara LS, Schuiteman A. Dendrobium armeniacum P.J.Cribb, a new record for Indonesian New Guinea[J]. Telopea, 2016, 19: 131-135.

686. Kadereit G. A new species of Dissochaeta Blume (Melastomataceae) from Kalimantan (Borneo, Indonesia)[J]. Edinburgh Journal of Botany, 2006, 63: 3-8.

687. Kalima, T., 2015. Keanekaragaman spesies rotan di Jawa Barat dan prospek pengembangan[J]. Prosiding Seminar Nasional Masyarakat Biodiversitas Indonesia, 2015, 1: 1802-1809.

688. Kandowangko NY, Latief M, Yusuf R. Short Communication: Inventory of traditional medicinal plants and their uses from Atinggola, North Gorontalo District, Gorontalo Province, Indonesia[J]. Biodiversitas, 2018, 19: 2294-2301.

689. Kartonegoro A. The Gesneriaceae of Sulawesi V: A new species of Rhynchoglossum and a new combination in Codonoboea[J]. Edinburgh Journal of Botany, 2012, 69: 357-361.

690. Kartonegoro A. Diplectria maxwellii (Melastomataceae), a new species from Sarawak, Borneo[J]. Kew Bulletin, 2018, 73: 23.

691. Kartonegoro A, Veldkamp JF. Revision of Creochiton (Melastomataceae)[J]. Blumea, 2013, 58: 217-227.

692. Kartonegoro A, Veldkamp JF, Hovenkamp P, et al. A revision of Dissochaeta (Melastomataceae, Dissochaeteae)[J]. PhytoKeys, 2018, 107: 1-178.

693. Keim AP. Pandanaceae of the island of Yapen, Papua (West New Guinea), Indonesia, with their nomenclature and notes on the rediscovery of Sararanga sinuosa, and several new species and records[J]. Blumea, 2009, 54: 255-266.

694. Klackenberg J. Four new species of Secamone (Apocynaceae, Secamonoideae) from Indonesia[J]. Blumea, 2006, 51: 587-597.

695. Klackenberg J. New species and combinations of Secamone (Apocynaceae, Secamonoideae) from South East Asia[J]. Blumea, 2010, 55: 231-241.
696. Kloet VSP. The Taxonomy of Vaccinium section Rigiolepis (Vaccinieae, Ericaceae)[J]. Blumea, 2005, 50: 477-497.
697. Kochaiphat P, Traiperm P, Utteridge TMA. Three new species of Erycibe (Convolvulaceae) from Malesia[J]. Phytotaxa, 2021, 494: 103-112.
698. Kulju KKM, Sierra SEC, van Welzen PC. Re-shaping Mallotus [Part 2]: inclusion of Neotrewia, Octospermum and Trewia in Mallotus s.s. (Euphorbiaceae s.s.)[J]. Blumea, 2007, 52: 115-136.
699. Kulju KKM, van Welzen PC. Revision of the genus Cleidion (Euphorbiaceae) in Malesia[J]. Blumea, 2005, 50: 197-219.
700. Kurniawan FY, Putri F, Suyoko A, et al. The diversity of wild orchids in the southern slope of Mount Merapi, Yogyakarta, Indonesia eight years after the 2010 eruption[J]. Biodiversitas, 2020, 21: 4457-4465.
701. Kusmana C, Hikmat A. The Biodiversity of Flora in Indonesia[J]. Journal of Natural Resources and Environmental Management, 2015, 5: 187-198.
702. Kusumastuti N, Suratman S, Pitoyo A. Orchids diversity on six forest types in Wasur National Park, Merauke, Papua, Indonesia[J]. Asian Journal of Forestry, 2021, 5: 101-110.
703. Lammers TG. Revision of the Malesian species of Lobelia section Rhynchopetalum (Campanulaceae: Lobelioideae)[J]. Blumea, 2011, 56: 218-224.
704. Leach GJ. Synopsis of the genus Eriocaulon (Eriocaulaceae) for New Guinea[J]. Australian Systematic Botany, 2018, 31: 420-432.
705. Lehnert M, Cámara-Leret R. New species of scaly tree ferns (Cyatheaceae) from New Guinea, and new combinations for the family for Malesia[J]. Kew Bulletin, 2019, 74: 46.
706. Lehnert M, Coritico FP. The genus Dicksonia (Dicksoniaceae-Cyatheales) in western Malesia[J]. Blumea, 2018, 63: 268-278.
707. Lense O. The wild plants used as traditional medicines by indigenous people of Manokwari, West Papua[J]. Biodiversitas, 2012, 13: 98-106.
708. Leong-Škorničková J, Lamb A, Linton J, et al. Six new Orchidantha species (Lowiaceae) from Borneo[J]. Gardens' Bulletin Singapore, 2021, 73: 179-202.
709. Lestari DA, Azrianingsih R, Hendrian H. Taxonomical position of Annonaceae species from East Java, Indonesia: Collections of Purwodadi Botanic Garden based on morphological character[J]. Biodiversitas, 2017, 18: 1067-1076.

710. Lestari DA, Darmayanti AS. Plants Flowering and Fruiting Behaviour in Alas Purwo National Park, Banyuwangi, East Java[J]. Journal of Tropical Biodiversity and Biotechnology, 2020, 5: 132-142.
711. Lestari DA, Santoso W. Inventory and habitat study of orchids species in Lamedai Nature Reserve, Kolaka, Southeast Sulawesi[J]. Biodiversitas, 2010, 12: 28-33.
712. Lestari WS, Adjie B. Studies on Fern of Lesser Sunda Islands I: Checklist of the Genus Adiantum (Pteridaceae)[J]. Jurnal Biodjati, 2020, 5: 107-114.
713. Lindstrom AJ, Hill KD, Stanberg LC. The genus Cycas (Cycadaceae) in Indonesia[J]. Telopea, 2009, 12: 385-418.
714. Lofthus Ø, Newman MF, Jimbo T, et al. The Pleuranthodium (Zingiberaceae) of Mount Wilhelm, Papua New Guinea[J]. Blumea, 2020, 65: 95-101.
715. Low YW, Wong KM. Two new species of Gardenia (Rubiaceae) from Borneo and notes on Gardenia Pterocalyx[J]. Edinburgh Journal of Botany, 2007, 64: 25-36.
716. Luong T-T, Hovenkamp PH, Sosef MSM. Revision of the fern genus Orthiopteris (Saccolomataceae) in Malesia and adjacent regions[J]. PhytoKeys, 2015, 53: 39-71.
717. M A, Zakaria R, Mansor M, et al. The flora composition of Sabang Island, Aceh, Indonesia[J]. Check List, 2012, 8: 600-609.
718. Maarif F, Rustiami H, Priyanti P. A Phenetic Analysis of Korthalsia spp. in Sumatra Based on Morphological Characters[J]. Jurnal Penelitian Hutan dan Konservasi Alam, 2021, 18: 67-82.
719. Macklin J, Parnell J. Dendromyza hiepkoana sp. nov. from Irian Jaya and D. staufferi sp. nov. from Papua New Guinea (Amphorogynaceae)[J]. Nordic Journal of Botany, 2016, 34: 169-173.
720. Nahdi MS, Kurniawan AP. The diversity and ethnobotanical study of medicinal plants in the southern slope of Mount Merapi, Yogyakarta, Indonesia[J]. Biodiversitas, 2019, 20: 2279-2287.
721. Nahdi MS, Kurniawan AP. Ethnobotanical study of medicinal plants in karst environment in Gunung Kidul, Yogyakarta, Indonesia[J]. Nusantara Bioscience, 2019, 11: 133-141.
722. Malcomber ST, Taylor CM. A Systematic Revision of Gaertnera (Rubiaceae, Gaertnereae)1[J]. Annals of the Missouri Botanical Garden, 2009, 96: 575-671.
723. Mambrasar YM, Damayanto IPGP, Atikah TD. Rediscovery Sumatran endemic flora: towards the establishment of data of biodiversity loss[J]. IOP Conference

Series: Earth and Environmental Science, 2019, 308: 012083.
724. Merrill ED, Arboretum A. A Brief Survey of the Present Status of Bornean Botany[J]. Webbia, 1950, 7: 309-324.
725. Middleton DJ. Three new species of Chilocarpus (Apocynaceae-Rauvolfioideae) from Malesia[J]. Edinburgh Journal of Botany, 2006, 63: 201-207.
726. Mildawati M, Sobir S, Sulistijorini S, et al. The diversity of pteridophytes in Siberut National Park, Mentawai Islands, West Sumatra, Indonesia[J]. Biodiversitas, 2020, 21: 3200-3208.
727. Mohamad S, Kalu M, Poulsen AD. A new species and a new combination of Sundamomum (Zingiberaceae) from Sarawak, Borneo[J]. Kew Bulletin, 2020, 75: 58.
728. Mols JB, Kessler PJA. The genus Miliusa (Annonaceae) in the Austro-Malesian area[J]. Blumea, 2003, 48: 421-462.
729. Mudiana D. Syzygium diversity in Gunung Baung, East Java, Indonesia[J]. Biodiversitas, 2016, 17: 733-740.
730. Mudiana D, Ariyanti EE. Field notes: terrestrial orchids in the Lappadata Forest, Bone, South Sulawesi[J]. IOP Conference Series: Earth and Environmental Science, 2020, 456: 012040.
731. Munawaroh E, Yuzammi. Species diversity of Orchids in Bukit Barisan Selatan National Park, Lampung, Indonesia[J]. Biodiversitas, 2018, 20: 343-349.
732. Munawaroh E, Yuzammi, Purwanto Y. The Euphorbiaceae (Spurge Family) in Bogor Botanic Gardens, Indonesia: Diversity, conservation and utilization[J]. Biodiversitas, 2020, 21: 5021-5031.
733. Mustaqim WA, Ardi WH. Ericaceae of Sulawesi: A new species of Diplycosia, a new variety of Vaccinium paludicolum and one rediscovery[J]. Telopea, 2019, 22: 193-204.
734. Mustaqim WA, Astuti IP. New and noteworthy orchid records from Buru Island, Maluku Archipelago[J]. Gardens' Bulletin Singapore, 2019, 71: 167-174.
735. Mustaqim WA, Astuti IP, Rahayu S. Hoya anulata (Apocynaceae: Asclepiadoideae) : a new record in Maluku, Indonesia[J]. Telopea, 2018, 21: 161-165.
736. Mustaqim WA, Low YW, Heatubun CD. A new species of Syzygium (Myrtaceae) from the Bird's Head Peninsula, western New Guinea[J]. Telopea, 2020, 23: 221-225.
737. Mustaqim WA, Putra HF. Thottea tapanuliensis (Aristolochiaceae): A new species

from Sumatra, Indonesia[J]. Telopea, 2020, 23: 163-168.
738. Mustaqim WA, Utteridge TMA, Heatubun CD. Diplycosia papuana (Ericaceae: Vaccinioideae: Gaultherieae): a new endemic species from central New Guinea[J]. Kew Bulletin, 2019, 74: 68.
739. Mustofa FI, Rahmawati N, Aminullah. Medicinal plants and practices of Rongkong Traditional Healers in South Sulawesi, Indonesia[J]. Biodiversitas, 2020, 21: 642-651.
740. Nahdi MS, Martiwi INA, Arsyah DC. The ethnobotany of medicinal plants in supporting the family health in Turgo, Yogyakarta, Indonesia[J]. Biodiversitas, 2016, 17: 900-906.
741. Naive MAK, Handoyo F, Ormerod P, et al. Dendrobium niveolabium (Orchidaceae, section Grastidium), a new Dendrobiinae species from Papua, Indonesia[J]. Phytotaxa, 2021, 490: 271-277.
742. Nasution T, Iskandar EAP, Ismaini L. Keragaman flora berpotensi dan komposisi vegetasi di Gunung Marapi, Sumatera Barat[J]. Prosiding Seminar Nasional Masyarakat Biodiversitas Indonesia, 2015, 1: 1334-1340.
743. Navia ZI, Suwardi AB, Nuraini. The importance of tropical edible fruit plants for tribal communities in East Aceh region, Indonesia[J]. IOP Conference Series: Earth and Environmental Science, 2021, 637: 012003.
744. Newman MF. A new species of Riedelia (Zingiberaceae) from Papua, Indonesia[J]. Edinburgh Journal of Botany, 2010, 67: 65-68.
745. Nishida S. Two new species of Beilschmiedia (Lauraceae) from Borneo[J]. Blumea, 2006, 51: 89-94.
746. Nishida S. Taxonomic revision of Beilschmiedia (Lauraceae) in Borneo[J]. Blumea, 2008, 53: 345-383.
747. Normasiwi S, Mutaqien Z, Noviady I, et al. Eksplorasi flora di kawasan hutan lindung Gunung Talamau, Sumatera Barat dan hutan lindung Gunung Sibuatan, Sumatera Utara untuk pengayaan koleksi Kebun Raya Cibodas[J]. Prosiding Seminar Nasional Masyarakat Biodiversitas Indonesia, 2015, 1: 501-508.
748. Wijana N, Rahmawati PI. Short Communication: Medicinal plants in Ubud Monkey Forest in Bali, Indonesia: Diversity, distribution, traditional use and tourism attractiveness[J]. Biodiversitas, 2020, 21: 2455-2461.
749. Ottens-Treurniet MAD, van Welzen PC. A revision of the Malesian genus Blumeodendron (Euphorbiaceae)[J]. Blumea, 2016, 61: 64-82.
750. Pandiangan D, Silalahi M, Dapas F, et al. Diversity of medicinal plants and their

uses by the Sanger tribe of Sangihe Islands, North Sulawesi, Indonesia[J]. Biodiversitas, 2019, 20: 611-621.

751. Perrie LR, Parris BS, Chen CW, et al. Asplenium alleniae (Aspleniaceae), a new fern species from Borneo and New Guinea[J]. Blumea, 2021: 219-223.
752. Petoe P, Heatubun CD, Baker WJ. A monograph of Hydriastele (Areceae, Arecaceae) in New Guinea and Australia[J]. Phytotaxa, 2018, 370: 1-92.
753. Pornpongrungrueng P, Gustafsson MHG, Borchsenius F, et al. Blumea (Compositae: Inuleae) in continental Southeast Asia[J]. Kew Bulletin, 2016, 71: 1.
754. Poulsen AD. One new name and new combinations of Malesian Zingiberaceae[J]. Blumea, 2003, 48: 523-527.
755. Poulsen AD, Leong-Škorničková J. Two new Orchidantha species (Lowiaceae) from Borneo[J]. Blumea, 2017, 62: 157-162.
756. Praptosuwiryo TN, Pribadi DO, Puspitaningtyas DM, et al. Inventorying the tree fern Genus Cibotium of Sumatra: Ecology, population size and distribution in North Sumatra[J]. Biodiversitas, 2011, 12: 204-211.
757. Nugroho NP, Octavia D. Inventarisasi Jenis Tanaman Penghasil Hasil Hutan Bukan Kayu Di Hutan Nagari Paru, Sijunjung, Sumatera Barat[J]. Jurnal Penelitian Hutan dan Konservasi Alam, 2020, 17: 21-33.
758. Purba EC, Nisyawati, Silalahi M. The ethnomedicine of the Batak Karo people of Merdeka sub-district, North Sumatra, Indonesia[J]. International Journal of Biological Research, 2016, 4: 181-189.
759. Puspitaningtyas DM. Orchid exploration in Mount Bintan Besar Protected Forest, Bintan Island, Riau Islands Province, Sumatra, Indonesia[J]. Biodiversitas, 2018, 19: 1081-1088.
760. Puspitaningtyas DM. Inventory and exploration of orchid in Polewali Mandar, West Sulawesi, Indonesia[J]. Biodiversitas, 2019, 20: 1887-1896.
761. Putri LSE, Dasumiati, Kristiyanto, et al. Ethnobotanical study of herbal medicine in Ranggawulung Urban Forest, Subang District, West Java, Indonesia[J]. Biodiversitas, 1970, 17: 172-176.
762. Qamariah N, Mulia DS, Fakhrizal D. Indigenous Knowledge of Medicinal Plants by Dayak Community in Mandomai Village, Central Kalimantan, Indonesia[J]. Pharmacognosy Journal, 2020, 12: 386-390.
763. Komara LL, Choesin DN, Syamsudin TS. Plant diversity after sixteen years post coal mining in East Kalimantan, Indonesia[J]. Biodiversitas, 2016, 17: 531-538.

764. Rahayu S, Rodda M. Hoya amicabilis sp. nov. (Apocynaceae, Asclepiadoideae), from Java discovered on Facebook[J]. Nordic Journal of Botany, 2019, 37: e02563.
765. Rahayu S, Rodda M. Hoya of Sumatra, an updated checklist, three new species, and a new subspecies[J]. European Journal of Taxonomy, 2019, 508: 1-23.
766. Rahayu SE, Oktapianti R, Matondang I. Ethnobotany survey of medicinal plants used for traditional maternal healthcare by Serawai tribe, Seluma district, Bengkulu-Indonesia[J]. Journal of Current Medical Research and Opinion, 2020, 3: 441-448.
767. Rahayu SM, Andini AS. Ethnobotanical Study on Medicinal Plants in Sesaot Forest, Narmada, West Lombok, Indonesia[J]. Biosaintifika: Journal of Biology & Biology Education, 2019, 11: 234-242.
768. Rahmawati N, Mustofa FI, Haryanti S. Diversity of medicinal plants utilized by To Manui ethnic of Central Sulawesi, Indonesia[J]. Biodiversitas, 2020, 21: 375-392.
769. Rahmawaty R, Samosir JB, Batubara R, et al. Diversity and distribution of medicinal plants in the Universitas Sumatera Utara Arboretum of Deli Serdang, North Sumatra, Indonesia[J]. Biodiversitas, 2019, 20: 1457-1465.
770. Nasution BR, Aththorick TA, Rahayu S. Medicinal plants used in the treatment of diabetes in Karo ethnic, North Sumatra, Indonesia[J]. IOP Conference Series: Earth and Environmental Science, 2018, 130: 012038.
771. Ramadhani S, Iskandar J, Partasasmita R, et al. Local knowledge of Sundanese village people on traditional medicine: A case study in Cibeurih Hamlet, Nagarawangi Village, Sumedang District, Indonesia[J]. Biodiversitas, 2021, 22: 2891-2898.
772. Rambey R, Susilowati A, Rangkuti AB, et al. Plant diversity, structure and composition of vegetation around Barumun Watershed, North Sumatra, Indonesia[J]. Biodiversitas, 2021, 22: 3250-3256.
773. Randi A, Wijedasa LS, Boyce PC, et al. Two new species of Hanguana (Hanguanaceae) from Kalimantan, Indonesia[J]. Blumea, 2021, 66: 101-105.
774. Ratnani DAS, Junitha IK, Kriswiyanti E, et al. The ethnobotany of Ngusaba ceremonial plant utilization by Tenganan Pegringsingan community in Karangasem, Bali, Indonesia[J]. Biodiversitas, 2021, 22: 2078-2087.
775. Akbarini D, Iskandar J, Partasasmita R. Collaborative planning for development of the Pelawan Biodiversity Park in Bangka, Indonesia[J]. Biodiversitas, 2017, 18:

1602-1610.

776. Rizki R, Nursyahra N, Fernando O. Study of Weeds as Traditional Medicinal Plants Used by Indigenous People of West Pasaman, Indonesia[J]. Journal of Tropical Horticulture, 2019, 2: 81-85.
777. Robiansyah I. Diversity and biomass of tree species in Tambrauw, West Papua, Indonesia[J]. Biodiversitas, 2018, 19: 377-386.
778. Rodda M, Rahayu S. A revision of the Hoya uncinata complex (Apocynaceae, Asclepiadoideae), with description of a new species[J]. Phytotaxa, 2018, 383: 252-258.
779. Rogers ZS. Phaleria stevensiana: A Distinctive New Species of Thymelaeaceae Endemic to Sulawesi, Indonesia[J]. Novon, 2017, 25: 473-481.
780. Rohmat S, Nisyawati, Rahayu SE. Diversity of medicinal plants for pregnancy and postpartum care of Dayak Ngaju tribe in Mantangai sub-district, Kapuas regency, Central Kalimantan[J]. Journal of Physics: Conference Series, 2019, 1317: 012088.
781. Roosita K, Kusharto CM, Sekiyama M, et al. Medicinal plants used by the villagers of a Sundanese community in West Java, Indonesia[J]. Journal of Ethnopharmacology, 2008, 115: 72-81.
782. Rujehan R, Matius P. Potential and management strategy of floral biodiversity in the coastal areas in East Kalimantan, Indonesia[J]. Biodiversitas, 2018, 19: 1130-1137.
783. Sagun VG, Levin GA. Four new species of Acalypha (Euphorbiaceae) from Malesia[J]. Blumea, 2007, 52: 351-359.
784. Sagun VG, Levin GA, van Welzen PC. Revision and phylogeny of Acalypha (Euphorbiaceae) in Malesia[J]. Blumea, 2010, 55: 21-60.
785. Siregar HM. The conservation of native, lowland Indonesian Begonia species (Begoniaceae) in Bogor Botanic Gardens[J]. Biodiversitas, 2017, 18: 326-333.
786. Santhyami S, Sulistyawati E. Medicinal Knowledge of Traditional Community in Kampung Dukuh, Garut Regency, West Java[J]. Al-Kauniyah: Jurnal Biologi, 2021, 14: 162-183.
787. Santi DM, Mulyaningsih T, Aryanti E. Identifikasi Bambu Di Sempadan Sungai Keremit Resort Joben Taman Nasional Gunung Rinjani Lombok[J]. Jurnal Biologi Tropis, 2019, 19: 239-249.
788. Saputra R. Orchids From Five Districts in Fakfak Regency, West Papua: Diversity and Distribution[J]. Jurnal Penelitian Hutan dan Konservasi Alam, 2021,

18: 29-38.
789. Sari RP, Yusro F, Mariani Y. Medicinal Plants Used by Dayak Kanayatn Traditional Healers in Tonang Village Sengah Temila District Landak Regency[J]. Jurnal Biologi Tropis, 2021, 21: 324-335.
790. Schuiteman A, de Vogel EF. New names and combinations in Orchidaceae from the Philippines and New Guinea[J]. Blumea, 2003, 48: 507-514.
791. Searle RJ, Newman MF. Myxochlamys amphiloxa (Zingiberaceae): A new species from Central Kalimantan, Indonesia[J]. Edinburgh Journal of Botany, 2010, 67: 347-352.
792. Setiawati T, Mutaqin AZ, Irawan B, et al. Species diversity and utilization of bamboo to support life's the community of Karangwangi Village, Cidaun Sub-District of Cianjur, Indonesia[J]. Biodiversitas, 2017, 18: 58-64.
793. Setyadi G, Pribadi R, Wijayanti DP, et al. Mangrove diversity and community structure of Mimika District, Papua, Indonesia[J]. Biodiversitas, 2021, 22: 3562-3570.
794. Setyawan AD, Sugiyarto, Susilowati A, et al. Diversity and distribution of Selaginella in the Province of Yogyakarta Special Region[J]. Prosiding Seminar Nasional Masyarakat Biodiversitas Indonesia, 2015, 1: 987-992.
795. Setyawan AD, Sugiyarto, Widiastuti A. Species diversity of Selaginella in the Dieng Plateau, Central Java[J]. Prosiding Seminar Nasional Masyarakat Biodiversitas Indonesia, 2015, 1: 980-986.
796. Shabdin Z, Culham A, Simpson DA, et al. Mapania multiflora, a distinctive new species of Cyperaceae (Mapanioideae) from Borneo[J]. Kew Bulletin, 2013, 68: 673-678.
797. Shahimi S, Conejero M, Prychid CJ, et al. A taxonomic revision of the myrmecophilous species of the rattan genus Korthalsia (Arecaceae)[J]. Kew Bulletin, 2019, 74: 69.
798. Sierra SEC, Aparicio M, Kulju KKM, et al. Re-shaping Mallotus [Part 1]: Expanded circumscription and revision of the genus Cordemoya (Euphorbiaceae)[J]. Blumea, 2006, 51: 519-540.
799. Sierra SEC, van Welzen PC. A taxonomic revision of Mallotus section Mallotus (Euphorbiaceae) in Malesia[J]. Blumea, 2005, 50: 249-274.
800. Sierra SEC, van Welzen PC, Slik JWF. A taxonomic revision of Mallotus section Philippinenses (former section Rottlera - Euphorbiaceae) in Malesia and Thailand[J]. Blumea, 2005, 50: 221-248.

801. Silalahi M. Diversity of medicinal plants in homegardens in Tanjung Julu village, North Sumatra, Indonesia[J]. International Journal of Biological Research, 2016, 4: 78-82.
802. Silalahi M, Khairiah A, Nisyawati N. Ethnomedicinal plants and practices related to pregnancy, childbirth, and postpartum healthcare of Minangkabau ethnic group, West Sumatra, Indonesia[J]. Biodiversitas, 2020, 21: 4597-4605.
803. Silalahi M, Nisyawati. The diversity of beneficial plants in the home-owned gardens of the Lingga Village, The Karo District, North Sumatra, Indonesia[J]. IOP Conference Series: Earth and Environmental Science, 2018, 203: 012006.
804. Silalahi M, Nisyawati N. The ethnobotanical study of edible and medicinal plants in the home garden of Batak Karo sub-ethnic in North Sumatra, Indonesia[J]. Biodiversitas, 2018, 19: 229-238.
805. Silalahi M, Nisyawati, Pandiangan D. Medicinal plants used by the Batak Toba Tribe in Peadundung Village, North Sumatra, Indonesia[J]. Biodiversitas, 2019, 20: 510-525.
806. Silalahi M, Supriatna J, Walujo EB, et al. Local knowledge of medicinal plants in sub-ethnic Batak Simalungun of North Sumatra, Indonesia[J]. Biodiversitas, 2014, 16: 44-54.
807. Simões AR, Silva H, Silveira P. The Convolvulaceae of Timor with special reference to East Timor[J]. Blumea, 2011, 56: 49-72.
808. Siswanto D, Batoro J. Ethnomedicinal survey of plants used by local society in Poncokusumo district, Malang, East Java Province, Indonesia[J]. Asian Journal of Medical and Biological Research, 2017, 3: 158-167.
809. Azis S, Zubaidah S, Mahanal S, et al. Local knowledge of traditional medicinal plants use and education system on their young of Ammatoa Kajang tribe in South Sulawesi, Indonesia[J]. Biodiversitas, 2020, 21: 3989-4002.
810. Sofiyanti N, Isda MN, Juliantari E, et al. The inventory and spore morphology of ferns from Bengkalis Island, Riau Province, Indonesia[J]. Biodiversitas, 2019, 20: 3223-3236.
811. Sofiyanti N, Marpaung AA, Suriatno R, et al. Jenis-Jenis Tumbuhan Paku Di Pulau Rangsang, Kepulauan Meranti, Riau Dan Karakteristik Morfologi-Palinologi[J]. Jurnal Biologi Tropis, 2020, 20: 102-110.
812. Soják J. Argentina recognita (Rosaceae, Potentilleae), a new species from New Guinea, with a key to the species known from the island[J]. Willdenowia, 2012, 42: 89-93.

813. Soják J. Potentilla L. (Rosaceae) and related genera in Asia (excluding the former USSR), Africa and New Guinea - Notes on Potentilla XXVIII[J]. Plant Diversity and Evolution, 2012, 130: 7-157.
814. Stoops E, van Welzen PC. A revision of Ptychopyxis (Euphorbiaceae) in southeast Asia[J]. Nordic Journal of Botany, 2013, 31: 094-112.
815. Subiakto A, Rachmat HH. Exploration, collection and conservation of dipterocarps in Riau Islands[J]. Prosiding Seminar Nasional Masyarakat Biodiversitas Indonesia, 2015: 1, 428-433.
816. Sudarmono S. Biodiversity of Medicinal Plants at Sambas Botanical Garden, West Kalimantan, Indonesia[J]. Journal of Tropical Life Science, 2018, 8: 116-122.
817. Sujarwo W, Keim AP, Savo V, et al. Ethnobotanical study of Loloh: Traditional herbal drinks from Bali (Indonesia)[J]. Journal of Ethnopharmacology, 2015, 169: 34-48.
818. Sukenti K, Hakim L, Indriyani S, et al. Ethnobotanical study on local cuisine of the Sasak tribe in Lombok Island, Indonesia[J]. Journal of Ethnic Foods, 2016, 3: 189-200.
819. Sulistiarini D, Arifiani D, Santika Y. New records of Orchidaceae from Bali, Indonesia[J]. Gardens' Bulletin Singapore, 2016, 68: 87-95.
820. Puspitaningtyas DM. Orchid inventory in Bantimurung-Bulusaraung National Park, South Sulawesi, Indonesia[J]. Biodiversitas, 2017, 18: 341-350.
821. Purwayantie S, Suryadi UE. Plant diversity and nutrient substances of native edible plant: Case study in Suka Maju and Tamao Villages, Kapuas Hulu District, West Kalimantan, Indonesia[J]. Biodiversitas, 2020, 21: 842-852.
822. Sumanon P, Eiserhardt WL, Balslev H, et al. Maesa brevipedicellata (Primulaceae), a new species from Papua New Guinea[J]. Blumea, 2020, 65: 83-85.
823. Sunarno B. Revision of the genus Labisia (Myrsinaceae)[J]. Blumea, 2005, 50: 579-597.
824. Sunarti S. Persebaran Syzygium endemik Jawa[J]. Prosiding Seminar Nasional Masyarakat Biodiversitas Indonesia, 2015, 1: 1093-1098.
825. Supiandi MI, Leliavia L, Syafruddin D, et al. Plant fruits used as food by the Dayak community of Tamambaloh in Labian Ira'ang Village, Kapuas Hulu District, Indonesia[J]. Biodiversitas, 2019, 20: 1827-1832.
826. Supiandi MI, Mahanal S, Zubaidah S, et al. Ethnobotany of traditional medicinal

plants used by Dayak Desa Community in Sintang, West Kalimantan, Indonesia[J]. Biodiversitas, 2019, 20: 1264-1270.
827. Suratman. Two new species of Morinda (Rubiaceae) from Sumatra and Borneo[J]. Blumea, 2011, 56: 24-27.
828. Suratman. The genus Gynochthodes (Rubiaceae) in Sumatra[J]. Blumea, 2018, 62: 230-239.
829. Susanti R, Zuhud EAM. Traditional ecological knowledge and biodiversity conservation: the medicinal plants of the Dayak Krayan people in Kayan Mentarang National Park, Indonesia[J]. Biodiversitas, 2019, 20: 2764-2779.
830. Susiarti S, Rahayu M, Rugayah. Diversity of Indonesian Medicinal Plant in The lowland Forest, Bodogol and Its Surrounding of Mount Gede-Pangrango National Park, West Java[J]. IOP Conference Series: Earth and Environmental Science, 2018, 166: 012021.
831. Susiarti S, Sambas EN. Local Knowledge on Medicinal Plants of Batak Mandailing and Nias Communities in Batang Toru, North Sumatra, Indonesia[J]. IOP Conference Series: Earth and Environmental Science, 2018, 197: 012008.
832. Sutomo S, Darma IDP, Priyadi A, et al. Short Communication: Trees species diversity and indicator species in Bedugul forest ecosystem, Bali, Indonesia[J]. Biodiversitas, 2018, 19: 2213-2218.
833. Suwardi AB, Mardudi, Navia ZI, et al. Documentation of medicinal plants used by Aneuk Jamee tribe in Kota Bahagia Sub-district, South Aceh, Indonesia[J]. Biodiversitas, 2020, 22: 6-15.
834. Suwardi AB, Navia ZI, Harmawan T. Ethnobotany and conservation of indigenous edible fruit plants in South Aceh, Indonesia[J]. Biodiversitas, 2020, 21: 1850-1860.
835. Taek MM, Prajogo BE, Agil M. Plants used in traditional medicine for treatment of malaria by Tetun ethnic people in West Timor Indonesia[J]. Asian Pacific Journal of Tropical Medicine, 2018, 11: 630-637.
836. Takeuchi W. A new fern and two floristic records from the Karius limestone of Papua New Guinea[J]. Edinburgh Journal of Botany, 2007, 64: 7-15.
837. Takeuchi W. Additions to the flora of the Kaijende Highlands, Papua New Guinea: Glochidion welzenii (Euphorbiaceae), a new species from the Paiela Limestone District[J]. Blumea, 2008, 53: 399-406.
838. Takeuchi W. Saurauia taylorii (Actinidiaceae), a distinctive new species from the Kaijende Highlands of Papua New Guinea[J]. Blumea, 2008, 53: 335-340.

839. Takeuchi W. Dysoxylum middletonianum (Meliaceae), a distinctive new species from the Southern Fold Mountains of Papua New Guinea[J]. Edinburgh Journal of Botany, 2009, 66: 347-353.
840. Takeuchi W, Pipoly J. Nomenclatural transfers in east Malesian Myrsine (Myrsinaceae), and the description of M. Warrae, a distinctive new species from ultrabasic environments in Papua New Guinea[J]. Edinburgh Journal of Botany, 2009, 66: 459-467.
841. Tallei TE, Nangoy MJ, Koneri R, et al. Biodiversity Assessment of Mt. Tumpa Forest Park, North Sulawesi, Indonesia[J]. Asian Journal of Biodiversity, 2015, 6: 1-21.
842. Thomas DC, Ardi WH, Hartutiningsih MS, et al. Two new species of Begonia (Begoniaceae) from South Sulawesi, Indonesia[J]. Edinburgh Journal of Botany, 2009, 66: 229-238.
843. Thomas DC, Ardi WH, Hughes M. Two new species of Begonia (Begoniaceae) from Central Sulawesi, Indonesia[J]. Edinburgh Journal of Botany, 2009, 66: 103-114.
844. Thomas DC, Ardi WH, Hughes M. Nine new species of Begonia (Begoniaceae) from South and West Sulawesi, Indonesia[J]. Edinburgh Journal of Botany, 2011, 68: 225-255.
845. Thomas DC, Bour A, Ardi WH. Begonia of the Matarombeo karst, Southeast Sulawesi, Indonesia, including two new species[J]. Gardens' Bulletin Singapore, 2018, 70: 163-176.
846. Thomas DC, Hughes M. Begonia varipeltata (Begoniaceae): A new peltate species from Sulawesi, Indonesia[J]. Edinburgh Journal of Botany, 2008, 65: 369-374.
847. Tjitrosoedirdjo SS, Zakaria R, Nurainas. Notes on Aeschynanthus (Gesneriaceae) of Sumatra, Indonesia[J]. Blumea, 2009, 54: 278-279.
848. Trimanto, Hapsari L. Botanical survey in thirteen montane forests of Bawean Island Nature Reserve, East Java Indonesia: Flora diversity, conservation status, and bioprospecting[J]. Biodiversitas, 2016, 17: 832-846.
849. Trimanto T, Shofiah F. Exploration of Flora Diversity and Recommending Species for Reclamation of Coal Mining with Biodiversity Concept in Besiq Bermai Forest, East Borneo[J]. Journal of Tropical Life Science, 2018, 8: 97-107.
850. Trimanto T, Siahaan F. Botanical Survey in Moyo Island, West Nusa Tenggara, Indonesia: Inventory of Flora Collection at Forest[J]. Journal of Tropical Life

Science, 2017, 7: 158-166.
851. Turner IM. Annonaceae of the Asia-Pacific region: names, types and distributions[J]. Gardens’ Bulletin Singapore, 2018, 70: 409-744.
852. Undaharta NKE, Ardaka IM, Kurniawan A, et al. Begonia bimaensis, a new species of Begonia from Sumbawa Island, Indonesia[J]. Gardens’ Bulletin Singapore, 2015, 67: 95-99.
853. Undaharta NKE, Ardi WH. Studies on Begonia (Begoniaceae) of the Moluccas III: A new Begonia from Seram, Indonesia[J]. Gardens’ Bulletin Singapore, 2016, 68: 279-285.
854. Utami N. Two new species of Impatiens (Balsaminaceae) from Batang Gadis National Park, North Sumatra, Indonesia[J]. Blumea, 2005, 50: 443-446.
855. Utami N. Impatiens marroninus, a new species of Impatiens (Balsaminaceae) from Sumatra, Indonesia[J]. Blumea, 2020, 65: 10-11.
856. Utaminingrum W, Nofrianti N, Hartanti D. Ethnomedicinal survey of traditional antidiabetic plants in Baturraden and Sumbang[J]. Medisains, 2020, 18: 43-51.
857. Utina R, Katili AS, Lapolo N, et al. Short Communication: The composition of mangrove species in coastal area of Banggai District, Central Sulawesi, Indonesia[J]. Biodiversitas, 2019, 20: 840-846.
858. Utteridge TMA. A revised circumscription of Maesa ruficaulis S. Moore (Primulaceae-Maesoideae). Contributions to the Flora of Mt Jaya, XVIII[J]. Kew Bulletin, 2013, 68: 683-686.
859. van Sam H, van Welzen PC. Revision of Annesijoa, Elateriospermum and the introduced species of Hevea in Malesia (Euphorbiaceae)[J]. Blumea, 2004, 49: 425-440.
860. van Welzen PC. Revision of the Malesian and Thai species of Sauropus (Euphorbiaceae: Phyllanthoideae)[J]. Blumea, 2003, 48: 319-391.
861. van Welzen PC. Revision of the Asian Genus Koilodepas (Euphorbiaceae)1[J]. Annals of the Missouri Botanical Garden, 2010, 97: 218-234.
862. van Welzen PC. Revision of Dicoelia (Phyllanthaceae; Euphorbiaceae s.l.)[J]. Blumea, 2011, 56: 209-213.
863. van Welzen PC. Bischofia and Hymenocardia (Phyllanthaceae) in Malesia[J]. Blumea, 2016, 61: 272-279.
864. van Welzen PC. The genus Baliospermum (Euphorbiaceae) in Malesia[J]. Blumea, 2018, 63: 125-129.
865. van Welzen PC, Arias Guerrero S, Arifiani D, et al. Weda, a new genus with two

new species of Euphorbiaceae-Crotonoideae from Halmahera (North Maluku, Indonesia) and phylogenetic relationships of the Australasian tribe Ricinocarpeae[J]. Journal of Systematics and Evolution, 2020, 59: 1000-1017.

866. van Welzen PC, Forster PI. A revision of Malesian Austrobuxus (Picrodendraceae/Euphorbiaceae s.l. subfam. Oldfieldioideae)[J]. Nordic Journal of Botany, 2010, 28: 189-195.
867. van Welzen PC, Kulju KKM, Sierra SEC, et al. Key to the Malesian species of Mallotus (Euphorbiaceae)[J]. Blumea, 2010, 55: 285-290.
868. van Welzen PC, Sierra SEC. The Mallotus wrayi complex (Euphorbiaceae)[J]. Blumea, 2006, 51: 373-388.
869. van Welzen PC, van Oostrum AF. Revision of the Malesian species of Dimorphocalyx (Euphorbiaceae)[J]. Blumea, 2015, 59: 191-201.
870. van Welzen PC, Winkel E. A revision of Ostodes (Euphorbiaceae) in Malesia[J]. Blumea, 2015, 59: 185-190.
871. Veldkamp JF. A revision of Dimeria (Gramineae-Dimeriinae) in Malesia with a note on Cymbachne[J]. Blumea, 2016, 61: 207-214.
872. Veldkamp JF, van den Boogaart MEB, Heidweiller J, et al. A revision of Mnesithea (Gramineae-Rottboelliinae) in Malesia and Thailand[J]. Blumea, 2013, 58: 277-292.
873. Vermeulen JJ. New species of Bulbophyllum from eastern Malesia (Orchidaceae)[J]. Nordic Journal of Botany, 2009, 26: 129-195.
874. Vermeulen JJ, de Vogel EF, Vogel APTM. Preliminary results of an orchid survey of New Britain, Papua New Guinea: five new species of Bulbophyllum (Orchidaceae)[J]. Blumea, 2010, 55: 278-284.
875. Victoriano M. A New Species of Nepenthes (Nepenthaceae) and Its Natural Hybrids from Aceh, Sumatra, Indonesia[J]. Reinwardtia, 2021, 20: 17-26.
876. Vink W. The Winteraceae of the Old World. VIII. Some Zygogynum species from New Guinea[J]. Blumea, 2016, 61: 41-50.
877. Waluyo J, Wahyuni D, Pujiastuti, et al. Exploration and Identification of Spermatophyta Plants Division that are potentially can be used for Medicine at Evergreen Forest taman Nasional Baluran Indonesia[J]. International Journal of Environment, Agriculture and Biotechnology, 2017, 2: 2303-2308.
878. Wearn JA, Darbyshire I. Hulemacanthus species (Acanthaceae: Barlerieae) in New Guinea[J]. Blumea, 2013, 57: 215-216.
879. Wearn JA, Mabberley DJ. Clerodendrum (Lamiaceae) in Borneo[J]. Systematic

Botany, 2011, 36: 1050-1061.

880. Wei ZY, Xia ZQ, Shu JP, et al. Phylogeny and Taxonomy on Cryptic Species of Forked Ferns of Asia[J]. Frontiers in Plant Science, 2021, 12: 748562.

881. Widodo P, Chikmawati T. Six new species of Syzygium (Myrtaceae) from Sumatra[J]. Edinburgh Journal of Botany, 2016, 73: 277-289.

882. Widodo P, Lucas E. Two new species of Syzygium (Myrtaceae) from North and West Sumatra[J]. Kew Bulletin, 2018, 73: 47.

883. Wijaya IMS, Defiani MR. Diversity and distribution of figs (Ficus: Moraceae) in Gianyar District, Bali, Indonesia[J]. Biodiversitas, 2020, 22: 233-246.

884. Wilkie P. A new species of Scaphium (Sterculioideae, Malvaceae / Sterculiaceae) from Borneo[J]. Edinburgh Journal of Botany, 2008, 65: 475-481.

885. Wilkie P, Argent GCG. A new species of Diplycosia (Ericaceae) from South Kalimantan, Indonesia[J]. Edinburgh Journal of Botany, 2016, 73: 139-142.

886. Wilmot-Dear CM, Friis I. Pouzolzia floresiana (Urticaceae), a new species from Flores, Nusa Tenggara Timur (Lesser Sunda Islands), Indonesia[J]. Edinburgh Journal of Botany, 2012, 69: 293-299.

887. Wilson HP, Jimbo T, Hagwood A, et al. Three new species of Begonia sect. Petermannia (Begoniaceae) from Sandaun Province, Papua New Guinea[J]. Gardens' Bulletin Singapore, 2020, 72: 275-284.

888. Wilson HP, Paul O, Hughes M. Begonia maguniana (Begoniaceae, Begonia sect. Oligandrae), a new species from New Guinea[J]. Edinburgh Journal of Botany, 2019, 77: 119-125.

889. Wiriadinata H, Ohashi H, Adema F. Notes on Malesian Fabaceae (Leguminosae-Papilionoideae). 16. The genus Mucuna[J]. Blumea, 2016, 61:
90-124.

890. Nurfadilah S, Hapsari L, Abywijaya IK. Species richness, conservation status, and potential uses of plants in Segara Anakan Area of Sempu Island, East Java, Indonesia[J]. Biodiversitas, 2017, 18: 1568-1588.

891. Wiryono, Mersyah R, Tarantona M. Flora of Danau Dusun Besar conservation forest in Bengkulu Province, Indonesia[J]. Biodiversitas, 2020, 21: 5640-5649.

892. Wiryono, Puteri VNU, Senoaji G. The diversity of plant species, the types of plant uses and the estimate of carbon stock in agroforestry system in Harapan Makmur Village, Bengkulu, Indonesia[J]. Biodiversitas, 2016, 17: 249-255.

893. Wongso S, Bastmeijer JD, Budianto H, et al. Six new Cryptocorynetaxa (Araceae) from Kalimantan, Borneo[J]. Willdenowia, 2017, 47: 325-339.

894. Wongso S, Hendrik, Jensen KR, et al. A new Cryptocoryne species (Araceae) from the Schwaner mountains, West Kalimantan, Indonesia[J]. Nordic Journal of Botany, 2020, 38: e02716.
895. Wongso S, Ipor IB, Tawan CS, et al. Cryptocoryne aura (Araceae), a new species from West Kalimantan, Indonesia[J]. Willdenowia, 2016, 46: 275-282.
896. Wulandari I, Hendrawan R, Husodo T, et al. Vegetation structure and composition in Ciletuh Geopark, Sukabumi, Indonesia[J]. Asian Journal of Forestry, 2018, 2: 54-61.
897. Wuu-Kuang S. Taxonomic revision of Cinnamomum (Lauraceae) in Borneo[J]. Blumea, 2011, 56: 241-264.
898. Yao TL. Three new species of Loxocarpus (Gesneriaceae) from Sarawak, Borneo[J]. Gardens' Bulletin Singapore, 2015, 67: 289-296.
899. Yeng WS. Studies on Schismatoglottideae (Araceae) of Borneo XXI-Two new species of the Schismatoglottis Calyptrata Group: Schismatoglottis heterodoxa and S. ranchanensis[J]. Willdenowia, 2012, 42: 255-260.
900. Yeng WS, Boyce PC. Schismatoglottideae (Araceae) of Borneo XIX-Piptospatha pileata, a remarkable new species from Kalimantan Timur, Indonesian Borneo[J]. Willdenowia, 2012, 42: 247-253.
901. Yeng WS, Boyce PC, Ling LS. Studies on Schismatoglottideae (Araceae) of Borneo XXIV-Two new species of Aridarum from Kalimantan, and notes on the Aridarum Burttii Complex[J]. Willdenowia, 2012, 42: 261-268.
902. Yu RY, van Welzen PC. A taxonomic revision of Trigonostemon (Euphorbiaceae) in Malesia[J]. Blumea, 2018, 62: 179-229.
903. Yu TY, Turner IM, Cheek M. Revision of Chassalia (Rubiaceae-Rubioideae-Palicoureeae) in Borneo, with 14 new species[J]. European Journal of Taxonomy, 2021, 738: 1-60.
904. Yudaputra A, Rahardjo P. Short Communication: Plant species richness and diversity in Karangsambung-Karangbolong National Geopark, Indonesia[J]. Biodiversitas, 2020, 21: 1735-1742.
905. Yulia ND, Budiharta S. Epiphytic orchids and host trees diversity at Gunung Manyutan Forest Reserve, Wilis Mountain, Ponorogo, East Java[J]. Biodiversitas, 2010, 12: 22-27.
906. Yuliana E, Hewindati YT, Winata ADI, et al. Diversity and characteristics of mangrove vegetation in Pulau Rimau Protection Forest, Banyuasin District, South Sumatra, Indonesia[J]. Biodiversitas, 2019, 20: 1215-1221.

907. Yusro F, Hardiansyah G, Erianto E, et al. Biodiversity of Medicinal Plants in Tawang Serimbak Forest, Ensaid Panjang Village, Sintang Regency[J]. Jurnal Biologi Tropis, 2020, 20: 245-255.
908. Yusro F, Pranaka R, Budiastutik I, et al. Diversity of Medicinal Plants Used by Traditional Healers of Dayak Desa Tribe in the Villages of Kebong and Merpak, Sintang Regency[J]. Jurnal Biologi Tropis, 2020, 20: 329-339.
909. Yuzammi. The diversity of aroids (Araceae) in Bogor Botanic Gardens, Indonesia: Collection, conservation and utilization[J]. Biodiversitas, 2018, 19: 140-152.
910. Zhu H, Roos MC, Ridsdale CE. A taxonomic revision of the Malesian species of Lasianthus (Rubiaceae)[J]. Blumea, 2012, 57: 1-102.
911. Brambach F, Leuschner C, Tjoa A, et al. Diversity, endemism, and composition of tropical mountain forest communities in Sulawesi, Indonesia, in relation to elevation and soil properties[J]. Perspectives in Plant Ecology, Evolution and Systematics, 2017, 27: 68-79.
912. Hansen B. Taxonomic revision of the S. E. Asian species of Isoglossa (Acanthaceae)[J]. Nordic Journal of Botany, 2008, 5: 1-13.
913. Hidayat S, Wightman G. The medicinal value of lalap (raw vegetable) in Sundanese society at Bogor, West Java, Indonesia[J]. The Beagle: Records of the Museums and Art Galleries of the Northern Territory, 2001, 17: 7-11.
914. Larsen K, Larsen SS. New taxa and nomenclatural combinations in Malesian Bauhinia (Leguminosae-Caesalpinioideae)[J]. Nordic Journal of Botany, 2008, 13: 657-665.
915. Ormerod P. A synopsis of Eria Lindl. section Cylindrolobus (Blume) Lindl. (Orchidaceae: Eriinae) in Malesia[J]. Harvard Papers in Botany, 2014, 19: 77-95.
916. Tange C. Cyanoneuron (Rubiaceae), a new genus from Borneo and Sulawesi[J]. Nordic Journal of Botany, 2008, 18: 147-158.
917. Versteegh C. An anatomical study of some woody plants of the mountain flora in the Tropics (Indonesia)[J]. Acta Botanica Neerlandica, 1968, 17: 151-159.
918. Wilmot-Dear CM, Friis I. The Old World species of Pouzolzia (Urticaceae, tribus Boehmerieae). A taxonomic revision[J]. Nordic Journal of Botany, 2008, 24: 5-111.
919. Henderson A. A revision of Calamus (Arecaceae, Calamoideae, Calameae, Calaminae)[J]. Phytotaxa, 2020, 445: 1-656.
