# Supplementary material for: Mapping Asia Plants: Plant Diversity and a Checklist of Vascular Plants in Indonesia
Source: Plants (Basel). 2024 Aug 16;13(16):2281. doi: 10.3390/plants13162281 (PMC11360604; doi:10.3390/plants13162281)
Supplement: Supplementary file 1 [file plants-13-02281-s001.zip › Document S1. Figures S1¿CS3 and Table S1.pdf]

Supplemental information

Mapping Asia Plants: Plant diversity and a checklist of vascular plants in Indonesia

Jing Sun, Bo Liu, Himmah Rustiami, Huiyun Xiao, Xiaoli Shen, and Keping Ma

Table S1. Similarity and statistics analysis of species in seven regions. Above the diagonal: number of shared species between two regions. Below the diagonal: Sørensen Index.

Region	Sumatra	Kalimantan	Java	Lesser Sunda Islands	Sulawesi	Maluku	Indonesian New Guinea
Sumatra	**10,902**	4,851	5,378	2,825	3,552	2,589	2,271
Kalimantan	0.48	**9,191**	3,458	1,955	2,905	2,121	1,864
Java	0.53	0.37	**9,289**	3,432	3,658	2,797	2,386
Lesser Sunda Islands	0.37	0.29	0.5	**4,514**	2,613	2,179	1,812
Sulawesi	0.4	0.36	0.45	0.45	**7,048**	3,033	2,553
Maluku	0.32	0.29	0.38	0.44	0.49	**5,294**	2,831
Indonesian New Guinea	0.22	0.19	0.25	0.25	0.3	0.37	**9,935**

.

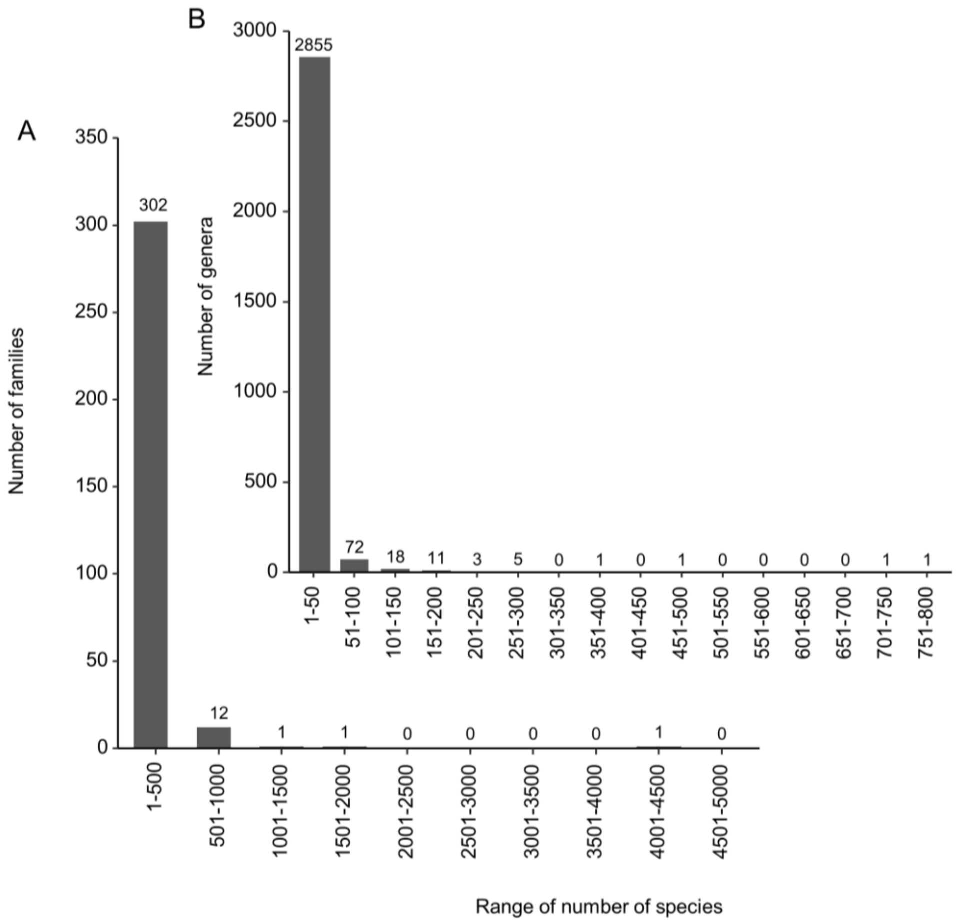

Figure S1. Number of families and genera versus number of species. (A) Number of families versus range of number of species. (B) Number of genera versus range of number of species.

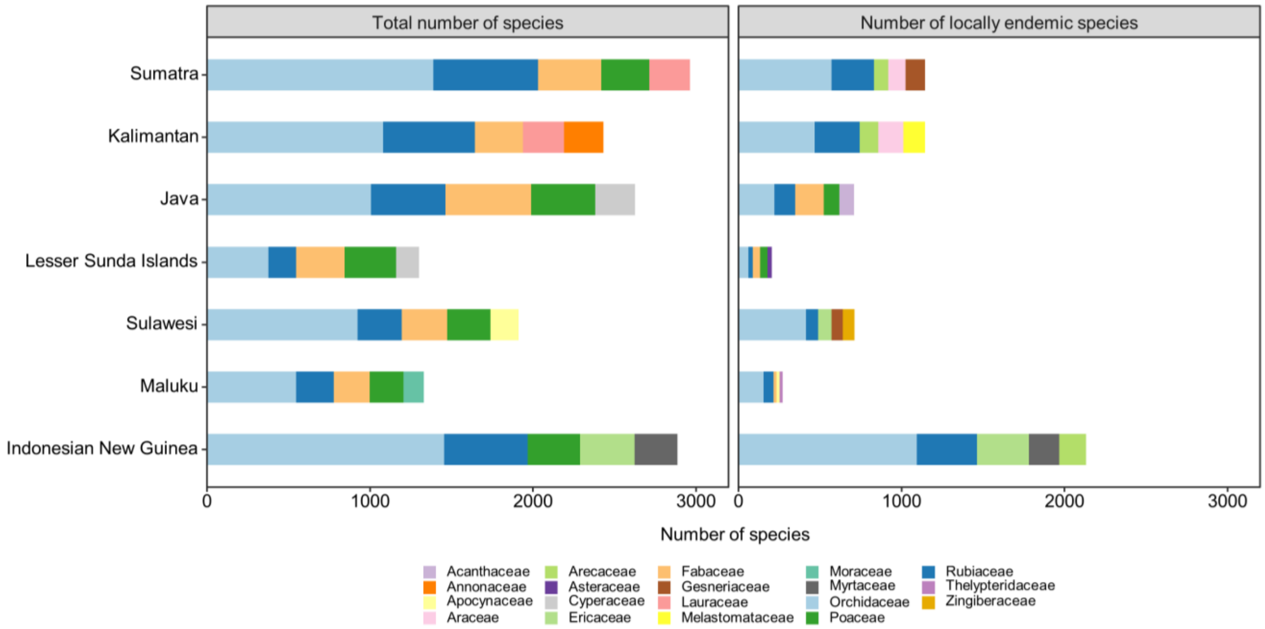

Figure S2. The top five families of total number of species and number of locally endemic species in each region.

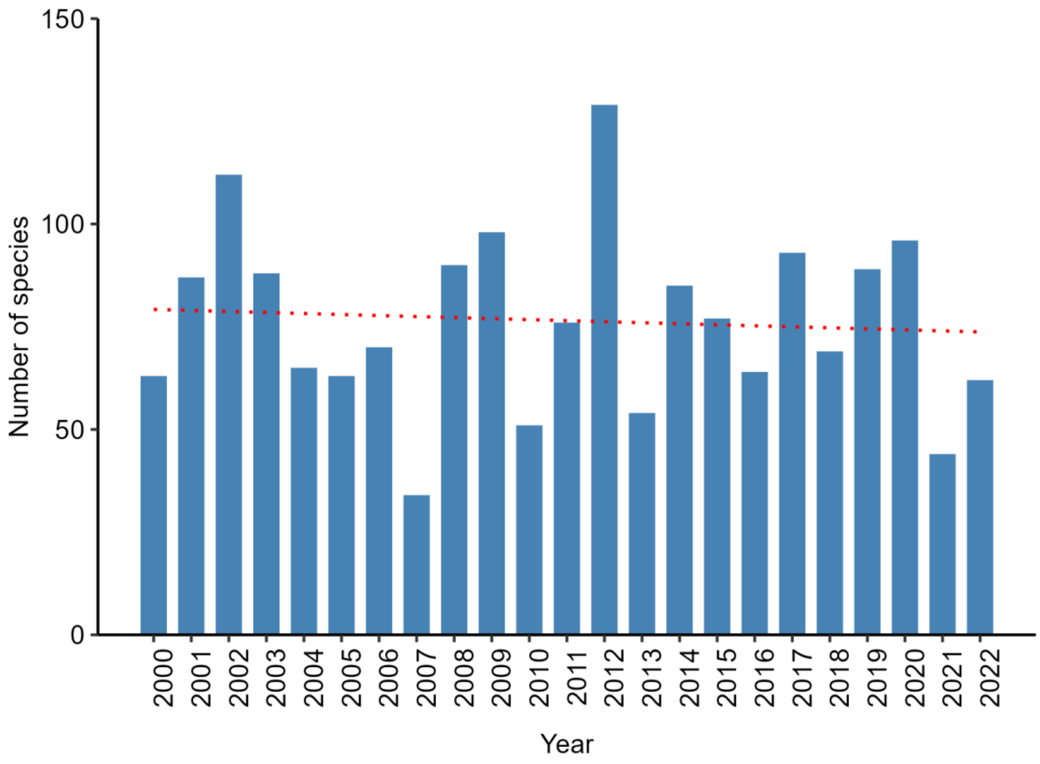

Figure S3. Increase in the number of new species of vascular plants published in Indonesia between 2000 and 2022. The dotted line is the trend. The data from International Plant Names Index (https://www.ipni.org/, accessed: July, 2023).
